# Relationships between professional value and workplace violence among healthcare workers: an empiricle study from China

**DOI:** 10.3389/fmed.2026.1768667

**Published:** 2026-04-21

**Authors:** Hongyu Luo, Shasha Li, Yanmei Chen, Xiumei Tang, Huairong Tang, Zhoufeng Wang

**Affiliations:** 1 Ruian People’s Hospital, The Third Affiliated Hospital of Wenzhou Medical University, Wenzhou, China; 2 Health Management Center, General Practice Medical Center, West China Hospital, Sichuan University, Chengdu, China; 3 Health Management Center, West China Tianfu Hospital, Sichuan University, Chengdu, China; 4 Institute of Hospital Management, West China Hospital, Sichuan University, Chengdu, China; 5 Institute of Respiratory Health, Frontiers Science Center for Disease-related Molecular Network, West China Hospital, Sichuan University, Chengdu, China

**Keywords:** cross sectional analysis, healthcare worker, professional values, survey, workplace violence

## Abstract

**Background:**

Workplace violence (WPV) against healthcare workers (HCWs) is a critical global public health issue that undermines staff safety, psychological well-being, and care quality. In China, HCWs experience substantial levels of WPV, yet evidence on modifiable factors negatively and positively associated with WPV, particularly those related to professional values remains limited.

**Methods:**

We conducted a multi-center cross-sectional survey among HCWs in 25 hospitals across China. A total of 4,267 respondents completed validated questionnaires assessing demographic and professional characteristics, professional values (intrinsic, extrinsic, social, altruistic, and leisure dimensions), and exposure to five types of WPV (physical violence, psychological abuse, threats, verbal sexual harassment, and physical sexual harassment) during the preceding 12 months. We used binary logistic regression to identify factors associated with any WPV exposure and ordered logistic models to examine correlates of increasing violence frequency.

**Results:**

More than half of participants (53.77%) reported psychological abuse in the previous year, 30.40% experienced threats, and 20.15% reported physical violence. Verbal and physical sexual harassment were less frequent but non-negligible (11.31 and 6.91%, respectively). Male HCWs reported higher overall WPV exposure than females in terms of violence frequency (OR = 0.686 for females in ordered logistic regression, *p* < 0.001). Higher monthly income (with OR reaching 2.376 for those earning >8,000 CNY versus <2,000 CNY; *p* < 0.001), and higher scores on intrinsic value dimension (OR = 1.037, *p* = 0.010) and altruistic value dimension (OR = 1.065, *p* = 0.015). Factors negatively associated with WPV included older age (>50 years; OR = 0.612, 95% CI 0.395–0.946; *p* < 0.05), holding an administrative position (OR = 0.530, 95% CI 0.377–0.746; *p* < 0.001), working outside general medicine (e.g., general surgery: OR = 0.786; auxiliary departments: OR = 0.719; both *p* < 0.05), being a formally employed staff member (OR = 0.555, 95% CI 0.322–0.960; *p* < 0.05), absence of night shift work (OR = 0.744, 95% CI 0.635–0.871; *p* < 0.001), no teaching responsibilities (OR = 0.798, 95% CI 0.679–0.939; *p* < 0.001), employment in a tertiary C hospital (OR = 0.111, 95% CI 0.013–0.979; *p* < 0.05), perceived alignment between workload and income (OR = 0.686, 95% CI 0.495–0.951; *p* < 0.05), and higher external, social, and leisure value scores. Ordered logistic models produced generally consistent patterns, indicating that these factors were also associated with the frequency and escalation of WPV.

**Conclusion:**

WPV against HCWs in China is highly prevalent and multifactorial, arising from the interaction of individual, professional, organizational, economic, and value-related factors. Our findings challenge conventional risk profiles—such as the assumption that female staff are universally at higher risk—and highlight the complex role of professional values, whereby strong intrinsic and altruistic orientations may paradoxically increase vulnerability. Targeted, multi-level prevention strategies should prioritize high-risk groups such as nurses, younger staff, and those working night shifts; enhance organizational safeguards and staffing; improve alignment between workload and compensation; and integrate staff well-being and professional value orientation into comprehensive violence prevention and support programs.

## Highlights

- Unlike most previous studies that focused primarily on objective factors (e.g., department, seniority, hospital level), this study systematically incorporates and quantifies multi-dimensional professional values (intrinsic, extrinsic, social, altruistic, and leisure values). It reveals their complex associations with both the occurrence and frequency of WPV, thereby expanding the theoretical framework of WPV research.
- The findings show that higher intrinsic and altruistic value scores are associated with increased exposure to WPV, whereas higher extrinsic, social, and leisure values are associated with decreased exposure to WPV. This challenges the conventional assumption that a stronger sense of professional mission necessarily reduces violence risk and provides a new explanatory lens for understanding emotional labor and doctor–patient interactions.
- In the Chinese hospital context, this study finds that male healthcare workers actually report a higher overall exposure to WPV than females. This highlights the context-dependent nature of gender-related risk and corrects the oversimplified notion that women are always the high-risk group, providing an empirical basis for developing gender-sensitive prevention strategies.
- Establishing a multi-level, operational framework for identifying high-risk groups. Using multivariable binary and ordered logistic regression models, the study integrates individual, professional, and organizational factors, including age, occupation (e.g., nurses), income level, department type, night-shift work, teaching responsibilities, employment status, hospital grade, and perceived workload, income balance, to construct a comprehensive risk, protection profile. This framework enables hospital managers to identify high-risk populations and critical scenarios more accurately and to allocate prevention resources more precisely.
- Beyond documenting the high prevalence and multi-type pattern of WPV among Chinese healthcare workers, the study underscores that prevention should not be limited to physical security measures and staffing adjustments. It emphasizes the importance of aligning professional value orientations with organizational arrangements (e.g., shift scheduling, remuneration, role assignment) and supports integrating value assessment into psychological support, training, and job design. These findings offer direct and practical guidance for policy-makers and hospital administrators in designing comprehensive WPV prevention and support programs.

## Background

Workplace violence (WPV) refers to incidents where staff is abused, threatened, or assaulted in circumstances related to their work (physical and psychological abuse), including from patients, relatives or other members of the public, involving an explicit or implicit challenge to their safety, well-being or health’. The definition varies from study to study since incivility, assault, aggressive, disruptive behavior and abuse may all be used in combination to define “violence” (1). Violence can manifest as physical assault, homicide, verbal abuse, bullying/mobbing, sexual and racial harassment, and psychological stress. WPV against health-care professionals is a significant problem around the world with prevalence ranging from 45.6 to 90%, with particularly concerning rates in emergency departments, psychiatric settings, and geriatric care units (2). China’s healthcare system faces similarly alarming statistics, with nationwide studies reporting that 65.8% of HCWs experience some form of workplace violence each year, compared with 70% in the US and 15% in UK (3–10). The consequences of WPV extend far beyond the immediate physical or psychological trauma experienced by individual HCWs. Substantial evidence demonstrates that exposure to WPV correlates with increased burnout, reduced job satisfaction, heightened turnover intention, and elevated rates of depression and anxiety among healthcare professionals (11–15). These individual-level impacts cascade into system-level consequences, including compromised quality of care, increased medical errors, reduced patient satisfaction, and significant economic costs through absenteeism, decreased productivity, and recruitment challenges.

Existing research has identified numerous factors positively associated with WPV in healthcare settings, though findings vary considerably across studies and contexts. Patient-related factors consistently associated with increased violence risk include mental illness; substance use disorders, dementia, and history of violent behavior (16–18). Healthcare worker characteristics linked to higher violence exposure include younger age, less experience, female gender (for sexual harassment), and direct patient care roles (19–21). Organizational factors significantly influence violence risk, with inadequate staffing, long wait times, overcrowding, poor security measures, and lack of violence prevention policies consistently associated with increased incidents (22, 23). Work-related factors such as night shifts, working alone, and high patient-to-staff ratios also correlate with higher violence rates (24, 25). Factors negatively associated with WPV have received comparatively less research attention but appear to include adequate staffing levels, comprehensive violence prevention training, clear reporting mechanisms, supportive leadership, and organizational justice. Recent studies suggest that positive workplace culture, team cohesion, and effective communication skills may buffer against violence risk (19, 20, 23, 26–28).

Professional values represent a particularly intriguing yet understudied domain in WPV research. The recent literature shows that WPV is consistently associated with erosion of healthcare workers’ professional values, attitudes and value-laden capacities (e.g., empathy, professional identity, perceived ethical climate), with these in turn mediating effects on health, burnout, and engagement, but almost no studies directly test how pre-existing professional values shape the occurrence, escalation, or reporting of violence (29–34). However, none of these quantitative studies examine whether baseline professional values predict risk of being targeted, escalation patterns, or likelihood of reporting violence, or distinguish value domains (e.g., caring vs. justice vs. integrity) to see which are most strongly implicated in violence dynamics. Furthermore, the relationship between professional values and WPV remains poorly understood, particularly in the Chinese healthcare context. Drawing upon the Job Demands-Resources (JD-R) model and emotional labor theory, we hypothesize that professional values may function as a “double-edged sword” in relation to WPV exposure (35). On one hand, strong intrinsic motivation and altruistic orientation, traditionally viewed as positive professional attributes, may increase HCWs’ vulnerability to violence through several mechanisms: (i) greater emotional investment in patient outcomes leading to boundary-blurring and over-engagement; (ii) reluctance to set firm limits with aggressive patients due to perceived professional duty; and (iii) higher exposure time through extended patient interactions driven by dedication (33, 36, 37). On the other hand, external value orientation (focus on compensation and status), social value orientation (emphasis on interpersonal relationships), and leisure value orientation (prioritization of work-life balance) may serve protective functions by: (i) facilitating psychological detachment that buffers against emotional escalation; (ii) promoting resource conservation that preserves capacity for de-escalation; and (iii) fostering social support networks that provide collective protection (38–40). This dual mechanism is particularly relevant in the Chinese healthcare context, where cultural values of professional dedication and patient-centeredness are deeply ingrained, potentially amplifying the paradoxical effects of professional values on WPV vulnerability. Unlike Western healthcare systems where individual boundaries are more culturally accepted, Chinese HCWs may face stronger normative pressures to demonstrate unlimited dedication, making the “double-edged sword” effect more pronounced. The role of professional values and orientations in WPV exposure represents an emerging area of inquiry (37, 41).

Despite the growing body of literature on WPV in healthcare, several critical knowledge gaps persist. First, most studies focus exclusively on nurses or physicians, neglecting the experiences of other healthcare professionals such as pharmacists, technicians, and administrative staff who also face violence risk. Second, research has predominantly examined physical violence, with less attention to psychological abuse, threats, and sexual harassment, despite evidence suggesting these may be more prevalent and similarly harmful. Third, few studies have employed comprehensive theoretical frameworks to examine the complex interplay between individual, organizational, and societal factors in WPV. Fourth, research on factors negatively associated with WPV remains limited compared to factors positively associated with WPV, hampering the development of strength-based prevention approaches. Finally, the relationship between HCWs’ professional values, work orientations, and violence exposure represents a significant knowledge gap despite its potential implications for prevention and intervention. Our study addresses critical knowledge gaps regarding workplace violence against HCWs in China through innovative methodological approaches and conceptual frameworks. This study assessed five types of WPV: physical violence, psychological abuse (behaviors that degrade, humiliate, or verbally insult), threats, verbal sexual harassment, and physical sexual harassment.

## Methods

### Study design and setting

Cross-sectional surveys were administered to staff at 25 hospitals across China between October 2022 and March 2023. We adhered to the Strengthening the Reporting of Observational studies in Epidemiology (STROBE) reporting guidelines (42).

### Study sample

The following types of staff were enrolled in this survey: (i) senior consultants, registrars, residents, and interns; (ii) registered nurses, specialist nurses, nurse practitioners, and nurse unit managers; (iii) dietitians, occupational therapists, pharmacists, physiotherapists, podiatrists, speech pathologists; and (iv) clinical assistants, patient service assistants, porters, volunteers, and ward clerks. After obtaining the approval of the administrations at each hospital, an email invitation to participate in the study was sent to the professional. Before accessing the questionnaires, respondents had to provide written informed consent to participate in the study. Respondents were ensured that their responses would remain anonymous, that their participation was voluntary, and that they could withdraw from the study anytime without repercussions. After providing consent, respondents were given access to the questionnaires (see below), which were expected to take approximately 15 min to complete, based on pilot tests with HCWs not involved in the full study. Detailed sample size calculation methods, sampling strategy and recruitment, eligibility criteria, participant flow, response rate, variable definition and category, detailed prevalence of each subtype of WPV can be seen in Supplementary files. This study was approved by the Ethics Committee of West China Hospital (No. 2023822).

### Theoretical framework

**Figure 1:**
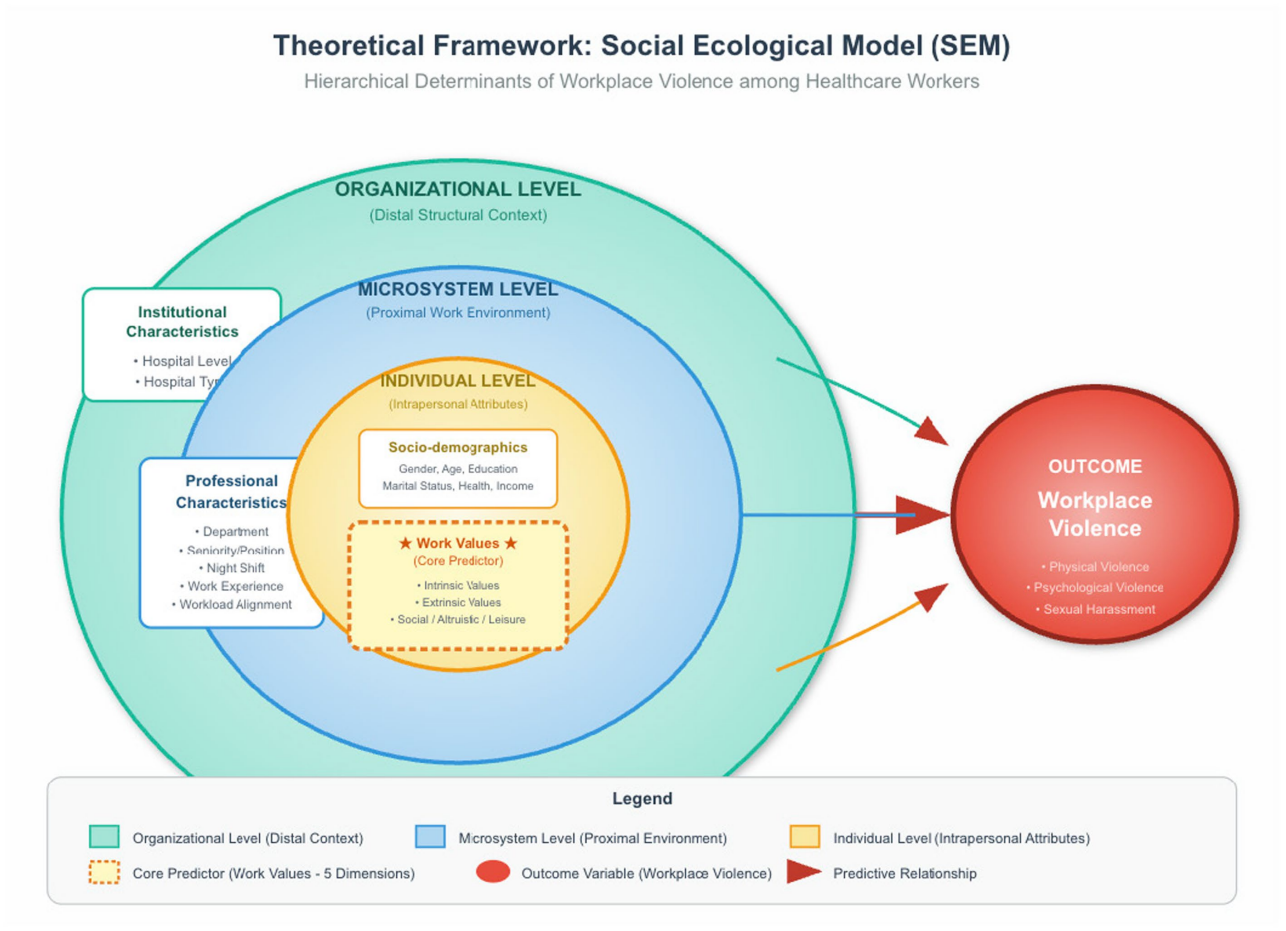
Theoretical framework based on the Social Ecological Model (SEM) for examining determinants of workplace violence among HCWs.

The study utilized the Social Ecological Model (SEM) to systematically examine the determinants of WPV. The framework conceptualizes WPV not merely as an isolated incident, but as a complex outcome resulting from the interplay between the individual professional and their multi-layered environment (43). Consequently, the explanatory variables were stratified into three hierarchical levels. At the individual level is the demographic and psychological attributes. Socio-demographic characteristics included variables such as gender, age, and education served as the biological and social baseline to control for individual vulnerability. Professional value is the psychological determinants. A key innovation of this study is the inclusion of professional values as intrapersonal cognitive drivers. Comprising five dimensions: intrinsic, extrinsic, social, altruistic, and leisure, and these values shape the HCWs’ professional identity and behavioral expectations (44). Within the SEM framework, these values are hypothesized to influence how professionals perceive and manage patient interactions, potentially acting as either WPV buffers or stimulators for conflict escalation. At the microsystem level is the professional characteristics. This level represents the proximal work environment where violence occurs. Variables such as seniority, department, night shift status, and workload-income alignment reflect the specific job demands (35). These factors define the intensity of patient contact and the situational stressors (e.g., fatigue, high-pressure settings) that directly trigger aggressive incidents. At the organizational level is the institutional characteristics. Hospital level and type constitute the distal structural context. These factors determine patient volume, case acuity, and the broader organizational safety climate, creating the background conditions that facilitate or inhibit the occurrence of violence. The Theoretical Framework can be seen in Figure 1.

### Survey instrument

#### Questionnaire to obtain primary demographic data

A custom-designed questionnaire of 25 items collected data on gender, age, marital status, education, average monthly income, health condition, and type of healthcare professional, as well as details about the respondent’s employment, including department, seniority, current position, working experience, type of employee, night shift work, teaching duty, perceived alignment between workload and income, type of hospital, and level of hospital (see in Supplementary files).

#### Questionnaire to determine exposure to workplace violence

Frequencies of different types of workplace violence were assessed using the Workplace Violence Scale (45). Respondents were asked how often they had experienced each of the following forms of workplace violence during the previous year: (i) physical attack, including grabbing, slapping, pushing, kicking, hitting, beating, biting, stabbing, or shooting; (ii) psychological abuse, including any behaviors that degrades, humiliates, irritates, alarms, or verbally insults; (iii) threat or intimidation in oral, written, physical, or other forms; (iv) verbal sexual harassment, including sexual comments, being asked about one’s sexual fantasies or preferences or history, being asked repeatedly on a date after having already declined, hearing rumors about another’s sex life or sexual preferences; and (v) physical, sexual harassment, such as receiving an unsolicited massage around the neck or shoulders, being touched in one’s hair or body, or being hugged, kissed, patted, touched or rubbed against sexually. Each item is scored on a 4-point scale reflecting respondents’ frequency of exposure to workplace violence (0 = 0 time, 1 = 1 time, 2 = 2–3 times and 3 = ≥4 times). The total possible score ranges from 0 to 27, with a higher total score indicating a higher frequency of exposure to workplace violence. The Workplace Violence Scale has demonstrated excellent psychometric properties across multiple studies (46–49). Internal consistency reliability was robust, with Cronbach’s alpha coefficients ranging from 0.89 to 0.93 for the overall scale. The five subscales also showed strong internal consistency: physical attack (*α* = 0.85–0.88), psychological abuse (α = 0.87–0.91), threat or intimidation (α = 0.83–0.86), verbal sexual harassment (α = 0.88–0.92), and physical sexual harassment (α = 0.86–0.90). The scale also exhibited strong validity across multiple dimensions. Content validity was established through expert review, with content validity indices (CVI) ranging from 0.89 to 0.95, demonstrating excellent agreement among experts regarding item relevance and representativeness.

*Questionnaire to assess professional values.* The 37-item Professional Value Questionnaire for Medical Staff (50), based on the Work Value Questionnaire (51) and Minnesota Satisfaction Questionnaire (52), was used to assess the strength of professional values across five dimensions. The *intrinsic dimension* (items 1–6) includes motivating factors arising from the work itself rather than from external factors or material incentives. These factors may include personal influence within the team, clarity of work goals, responsibilities, feedback from superiors and colleagues, accountability, anxiety, and interest. The *external dimension* (items 8–13) refers to motivational benefits, including salary, social status, prestige, wealth, opportunities to participate in decision-making, resources, promotion, and compensation. The *social dimension* (items 14–24) refers to incentives arising within the hospital, including working relationships, professional recognition, respect, fairness, human resource support, vocational training, family support, and a sense of security. The *altruistic dimension* (items 25–27) refers to incentives arising through participation in social services, including contributions to society, help to others, and satisfaction from serving others. The *leisure dimension* (items 28–37) refers to work-like balance and may include employee benefits, autonomy, personal freedom, working style, working environment, stability, and convenience of travel between home and work. Respondents responded to all items on a five-point Likert scale from 1 (“strongly disagree”) to 5 (“strongly agree”). Scores for each dimension and for the entire questionnaire were obtained through simple summation. Higher scores indicated the respondents had a more substantial commitment (adherence) to professional values. The 37-item Professional Value Questionnaire for Medical Staff has demonstrated strong psychometric properties in assessing HCWs’ professional values (53–57). The scale exhibited excellent internal consistency reliability, with Cronbach’s alpha coefficients ranging from 0.91 to 0.94 for the overall questionnaire. The five subscales also showed satisfactory to good internal consistency: intrinsic dimension (*α* = 0.82–0.87), external dimension (α = 0.79–0.85), social dimension (α = 0.88–0.92), altruistic dimension (α = 0.76–0.81), and leisure dimension (α = 0.84–0.89). Content validity was established through expert review, with CVI ranging from 0.87 to 0.93 at the scale level and 0.83 to 0.98 at the item level.

### Statistical analysis

Data were tabulated using EpiData Analysis software version V2.2.2.182 (EpiData Odense, Denmark). Statistical analyses, as well as all correlation and regression analyses, were performed in SPSS for Windows 22.0 (IBM, Chicago, IL, USA). Descriptive statistics, including number (n), percentages (%), mean and standard deviation (SD), were calculated for the demographic variables. The ordered Logit model and partial proportional odds model were performed to explore related factors negatively and positively associated with WPV.

#### Model selection: binary vs. ordered logistic regression

We employed a two-tiered modeling strategy, utilizing binary logistic regression for initial identification of factors associated with WPV and ordered logistic regression for a more granular analysis of WPV frequency. The binary logistic model served as our foundational analytical tool to identify key demographic, professional, and institutional factors associated with the occurrence versus non-occurrence of WPV. By dichotomizing WPV into an “ever experienced” versus “never experienced” outcome, we were able to identify high-risk groups. Ordered logistic regression represents a significant methodological advancement by analyzing WPV as an ordinal variable (e.g., Never < Once < 2–3 times < ≥4 times). The combined use of these two models provides a comprehensive understanding of WPV. The binary logistic regression establishes the broad landscape of risk, while the ordered logistic regression delves into the terrain, revealing critical nuances about the drivers of chronic and escalating aggression. This dual approach not only enhances the scientific rigor of our findings but also offers more targeted and actionable insights for developing effective prevention strategies tailored to the complex realities of healthcare environments.

## Results

### Participants

**Figure 2:**
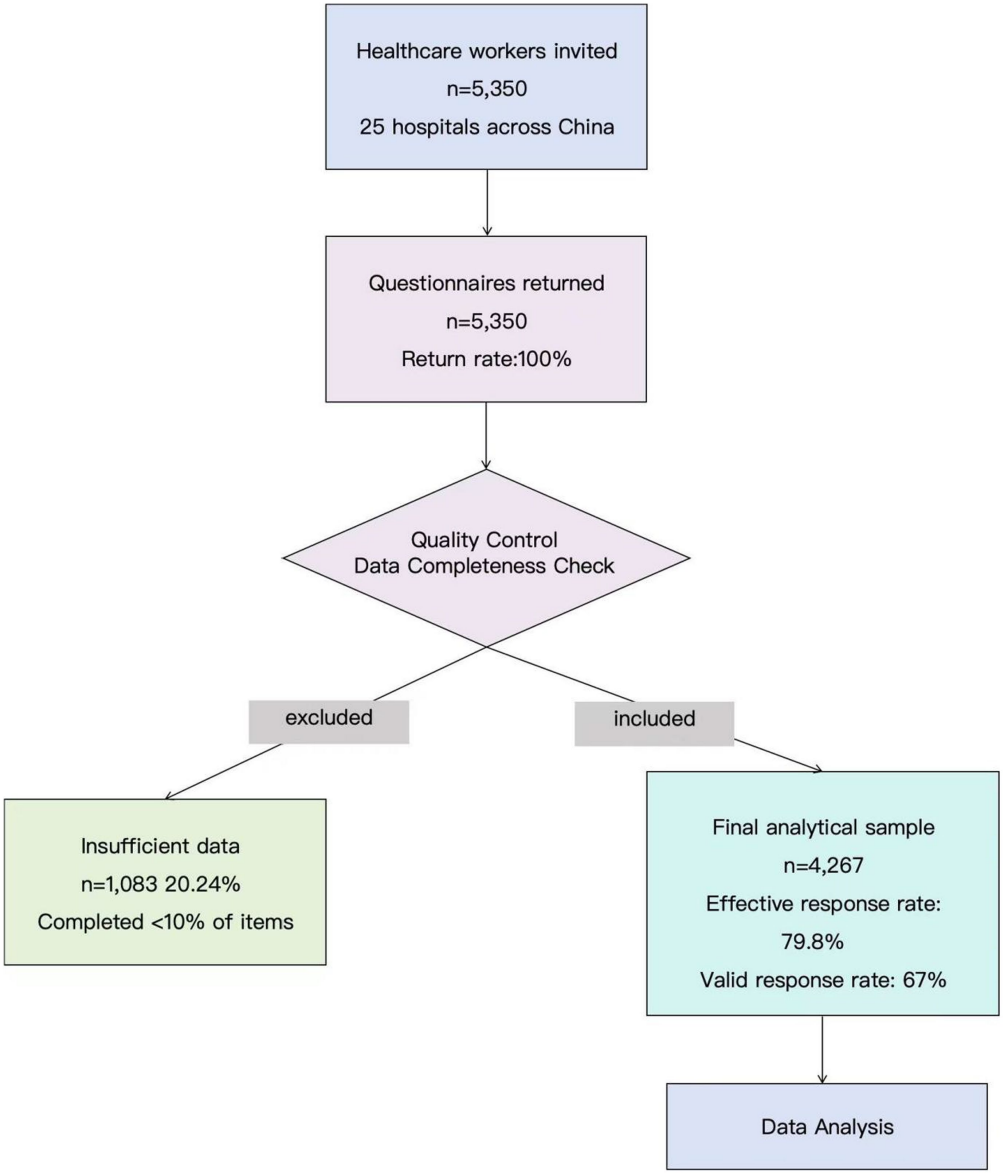
Research flow diagram.

We invited 5,350 HCWs at 25 hospitals across China to participate in the study. Of 5,350 respondents who returned questionnaires, we included 4,267 respondents in the analysis, corresponding to a response rate of 67%. The detailed process of participants recruitment can be seen in Supplementary files. The research flow diagram can be seen in Figure 2.

### Demographic characteristics of included HCWs

The sample comprised 3,168 women (74.2%), with a mean age predominantly in the 30–40 years range (44.5%, *n* = 1,898). The majority were married (78.6%, *n* = 3,352) and held undergraduate degrees (59.5%, *n* = 2,538). Nurses and midwives constituted the largest professional group (44.5%, *n* = 1,899), followed by physicians (32.4%, *n* = 1,382), allied health professionals (9.0%, *n* = 385), administrative staff (7.9%, *n* = 338), and pharmacists (2.7%, *n* = 117). Participants were distributed across general medicine (31.7%, *n* = 1,352), general surgery (26.9%, *n* = 1,147), medical auxiliary departments (15.5%, *n* = 663), and other specialties (25.9%, *n* = 1,105). Regarding seniority, 31.7% (*n* = 1,352) held senior titles, the majority were frontline employees (75.8%, *n* = 3,236), with 20.6% (*n* = 878) in department management positions. Over half of participants (50.9%, *n* = 2,171) had more than 10 years of clinical experience, while 27.4% (*n* = 1,171) had 6–10 years. Night shift work was common (63.4%, *n* = 2,707), and nearly half (49.9%, *n* = 2,128) held teaching responsibilities. Employment types included contract employees (51.2%, *n* = 2,183) and labor contract employees (46.7%, *n* = 1,991). Most participants worked in general hospitals (84.3%, *n* = 3,597) rather than specialized facilities (15.7%, *n* = 670). Hospital levels included tertiary-level A (45.5%, *n* = 1,943), tertiary-level B (23.5%, *n* = 1,002), secondary-level A (22.7%, *n* = 968), and community health centers or private hospitals (5.5%, *n* = 233), reflecting the Chinese hospital classification system (Table 1).

**Table 1:** Demographic and professional characteristics of the study sample (*N* = 4,267).

Characteristic	*n* (%)
Socio-demographic characteristics	
Gender	
Female	3,168 (74.2)
Male	1,099 (25.8)
Age, yr	
< 30	1,249 (29.3)
30–40	1,898 (44.5)
40–50	878 (20.6)
> 50	242 (5.7)
Marital status	
Single/living with partner	783 (18.4)
Married	3,352 (78.6)
Separated/divorced	121 (2.8)
Widowed	11 (0.3)
Education	
2-year associate’s degree	128 (3)
3-year bachelor’s degree	1,181 (27.7)
Undergraduate diploma	2,538 (59.5)
Master’s degree	357 (8.4)
Doctoral degree	63 (1.5)
Average monthly income, CNY	
< 2000	129 (3)
2000–4,000	702 (16.5)
4,000–6,000	1,248 (29.2)
6,000–8,000	1,061 (24.9)
> 8,000	1,127 (26.4)
Health condition (rate 0–10)	
Poor (0–2)	55 (1.3)
Bad (3–4)	463 (10.9)
Normal (5–6)	1,573 (36.9)
Healthy (7–8)	1,907 (44.7)
Good (9–10)	269 (6.3)
Professional and job-related characteristics	
Type of healthcare professional	
Physician	1,382 (32.4)
Nurse/midwife	1,899 (44.5)
Pharmacist	117 (2.7)
Allied health professional (therapist/radiographer/assistant)	385 (9)
Administrative or clerical worker	338 (7.9)
Other	146 (3.4)
Department	
General medicine	1,352 (31.7)
General surgery	1,147 (26.9)
Medical auxiliary/ancillary	663 (15.5)
Other	1,105 (25.9)
Seniority	
Senior	1,352 (31.7)
Deputy senior	1,147 (26.9)
Intermediate	663 (15.5)
Junior	1,105 (25.9)
Not reported	372 (8.7)
Current position	
Hospital manager	83 (1.9)
Department manager	878 (20.6)
Employee	3,236 (75.8)
Intern/student/trainee	70 (1.6)
Working experience, yr	
< 1	183 (4.3)
1–5	742 (17.4)
6–10	1,171 (27.4)
> 10	2,171 (50.9)
Type of employee	
Employee under labor contract	1,991 (46.7)
Employee under contract	2,183 (51.2)
Temporary worker	93 (2.2)
Night shift work	
Yes	2,707 (63.4)
No	1,560 (36.6)
Teaching duty	
Yes	2,128 (49.9)
No	2,139 (50.1)
Alignment between workload and income	
Very poor	351 (8.2)
Poor	1,224 (28.7)
Normal	1,617 (37.9)
Aligned	995 (23.3)
Well	80 (1.9)
Institutional characteristics	
Type of hospital	
General	3,597 (84.3)
Specialized	670 (15.7)
Level of hospital	
Tertiary A	1,943 (45.5)
Tertiary B	1,002 (23.5)
Tertiary C	7 (0.2)
Secondary A	968 (22.7)
Secondary B	109 (2.6)
Secondary C	5 (0.1)
Health center/private hospital	233 (5.5)
Professional values	
Intrinsic value	23.34 (4.031)
External value	20.26 (4.73)
Social value	35.70 (6.83)
Altruism value	11.54 (2.15)
Leisure value	28.10 (6.77)
Total scores	118.95 (21.68)

“1 CNY = 0.14 USD”. Level of hospital refers to the Chinese hospital classification.

### Prevalence of overall workplace violence in different populations

The overall prevalence of WPV in the preceding 12 months was 57.6% (2,458/4,267). Significant disparities in WPV prevalence were observed across demographic, professional, and institutional characteristics (Table 2). Regarding demographic, male HCWs (60.5%), those in the 30–40 age group (60.7%), being separated/divorced (61.2%), and those with master’s degree (65.5%) reported highest incidence of WPV. And a clear gradient was observed regarding income, alignment between workload, and health condition: HCWs with higher income, poorer alignment between workload, and poorer health condition, reported higher encounters of WPV. In terms of professional or job-related characteristics, physicians reported the highest incidence of WPV (65.8%), followed significantly by nurses/midwives (59.0%) and allied health professionals (47.5%), while administrative staff reported the lowest incidence (38.8%). Similarly, those working in the general medicine had the highest rate of WPV (65.8%), then general surgery (61.8%), and medical auxiliary departments (50.2%). When comes to seniority and experience, those who working as mid-level administrators in hospitals (such as department heads), having medical professionals with intermediate titles had the highest incidence of WPV: with deputy senior titles (68.5%) and being department managers (61.8%). Working experience also shows a gradient effect: longer working experience, higher incidence of WPV (60.3%). Employee under labor contract had highest incidence of WPV (60.6%), and those with night-shift work (62.5%) and teaching duty (65.2%). As for institutional characteristics, HCWs working in general hospitals (58.6%) and Tertiary A hospitals (62.5%) had the highest incidence of WPV.

**Table 2:** Frequency of workplace violence among participants stratified by demographic or professional characteristics.

Frequency of overall workplace violence in the preceding 12 months, no. participants (%)				
Characteristic	No	Yes	χ2	*p*
Socio-demographic characteristics				
Gender			4.96	0.026
Female	1,375 (43.4)	1793 (56.6)		
Male	434 (39.5)	665 (60.5)		
Age, yr			22.65	< 0.001
< 30	593 (47.5)	656 (52.5)		
30–40	746 (39.3)	1,152 (60.7)		
40–50	359 (40.9)	519 (59.1)		
> 50	111 (45.9)	131 (54.1)		
Marital status			10.48	0.015
Single/living with partner	370 (47.3)	413 (52.7)		
Married	1,389 (41.4)	1963 (58.6)		
Separated/divorced	47 (38.8)	74 (61.2)		
Widowed	3 (27.3)	8 (72.7)		
Education			45.05	< 0.001
2-year associate’s degree	75 (58.6)	53 (41.4)		
3-year bachelor’s degree	569 (48.2)	612 (51.8)		
Undergraduate diploma	1,018 (40.1)	1,520 (59.9)		
Master’s degree	123 (34.5)	234 (65.5)		
Doctoral degree	24 (38.1)	39 (61.9)		
Average monthly income, CNY			72.97	< 0.001
< 2000	69 (53.5)	60 (46.5)		
2000–4,000	362 (51.6)	340 (48.4)		
4,000–6,000	579 (46.4)	669 (53.6)		
6,000–8,000	408 (38.5)	653 (61.5)		
> 8,000	391 (34.7)	736 (65.3)		
Health condition (rate 0–10)			192.36	< 0.001
Poor (0–2)	14 (25.5)	41 (74.5)		
Bad (3–4)	111 (24.0)	352 (76.0)		
Normal (5–6)	557 (35.4)	1,016 (64.6)		
Healthy (7–8)	962 (50.4)	945 (49.6)		
Good (9–10)	165 (61.3)	104 (38.7)		
Professional and job-related characteristics				
Type of healthcare professional			129.45	< 0.001
Physician	472 (34.2)	910 (65.8)		
Nurse/midwife	779 (41.0)	1,120 (59.0)		
Pharmacist	60 (51.3)	57 (48.7)		
Allied health professional (therapist/radiographer/assistant)	202 (52.5)	183 (47.5)		
Administrative or clerical worker	207 (61.2)	131 (38.8)		
Other	89 (61.0)	57 (39.0)		
Department			104.33	< 0.001
General medicine	463 (34.2)	889 (65.8)		
General surgery	438 (38.2)	709 (61.8)		
Medical auxiliary/ancillary	330 (49.8)	333 (50.2)		
Other	578 (52.3)	527 (47.7)		
Seniority			102.38	< 0.001
Senior	37 (34.3)	71 (65.7)		
Deputy senior	204 (31.5)	443 (68.5)		
Intermediate	551 (39.6)	840 (60.4)		
Junior	786 (44.9)	963 (55.1)		
Not reported	231 (62.1)	141 (37.9)		
Current position			10.75	0.013
Hospital manager	35 (42.2)	48 (57.8)		
Department manager	335 (38.2)	543 (61.8)		
Employee	1,402 (43.3)	1834 (56.7)		
Intern/student/trainee	37 (52.9)	33 (47.1)		
Working experience, yr			56.74	< 0.001
< 1	116 (63.4)	67 (36.6)		
1–5	365 (49.2)	377 (50.8)		
6–10	467 (39.9)	704 (60.1)		
> 10	861 (39.7)	1,310 (60.3)		
Type of employee			15.81	< 0.001
Employee under labor contract	785 (39.4)	1,206 (60.6)		
Employee under contract	975 (44.7)	1,208 (55.3)		
Temporary worker	49 (52.7)	44 (47.3)		
Night shift work			71.15	< 0.001
Yes	1,016 (37.5)	1,691 (62.5)		
No	793 (50.8)	767 (49.2)		
Teaching duty			100.33	< 0.001
Yes	740 (34.8)	1,388 (65.2)		
No	1,069 (50.0)	1,070 (50.0)		
Alignment between workload and income			174.36	< 0.001
Very poor	97 (27.6)	254 (72.4)		
Poor	385 (31.5)	839 (68.5)		
Normal	728 (45.0)	889 (55.0)		
Aligned	552 (55.5)	443 (44.5)		
Well	47 (58.8)	33 (41.2)		
Institutional characteristics				
Type of hospital			9.63	0.002
General	1,488 (41.4)	2,109 (58.6)		
Specialized	321 (47.9)	349 (52.1)		
Level of hospital			46.3	< 0.001
Tertiary A	728 (37.5)	1,215 (62.5)		
Tertiary B	436 (43.5)	566 (56.5)		
Tertiary C	6 (85.7)	1 (14.3)		
Secondary A	473 (48.9)	495 (51.1)		
Secondary B	54 (49.5)	55 (50.5)		
Secondary C	2 (40.0)	3 (60.0)		
Health center/private hospital	110 (47.2)	123 (52.8)		

Data are presented as No. (row, %).

Calculated using Pearson chi-square test or Fisher’s exact test where appropriate.

### Prevalence of overall workplace violence and its 5 subtypes in different populations

Among the five types of WPV examined, emotional abuse was the most prevalent (54.7%, *n* = 2,294), followed by threats and intimidation (30.5%, *n* = 1,303), physical assault (20.2%, *n* = 860), verbal sexual harassment (11.5%, *n* = 489), and physical sexual harassment (7.0%, *n* = 297). Across all violence types, significant gender disparities were observed, with males reporting higher rates of physical assault (25.8% vs. 18.2%, *p* < 0.001), threats and intimidation (37.6% vs. 27.9%, *p* < 0.001), and both verbal (15.8% vs. 9.8%, *p* < 0.001) and physical sexual harassment (8.0% vs. 6.5%, *p* = 0.033) compared to females. Self-rated health status demonstrated a strong dose–response relationship with all violence types, with those rating health as poor (0–2) experiencing substantially higher rates: physical assault (47.3%), emotional abuse (72.7%), threats and intimidation (58.2%), verbal sexual harassment (25.5%), and physical sexual harassment (16.4%) compared to those with good health (all *p* < 0.001) (Supplementary Table 1). Professional characteristics showed consistent patterns, with physicians and nurses/midwives experiencing the highest rates across all violence types (all *p* < 0.001). Work-related factors including night shift work (all p < 0.001), teaching responsibilities (all *p* < 0.001), and perceived poor alignment between workload and income (all *p* < 0.001) were significantly associated with increased exposure to all violence types. Notably, the frequency of repeated exposure was concerning, with 9.4% experiencing physical assault more than 2 times, 37.7% experiencing emotional abuse more than 2 times, and 18.9% experiencing threats and intimidation more than 2 times in the preceding 12 months. The detailed analysis of five subtypes of WPV can be seen in Supplementary files.

### Binary logistic regression to identify factors negatively and positively associated with WPV

**Figure 3:**
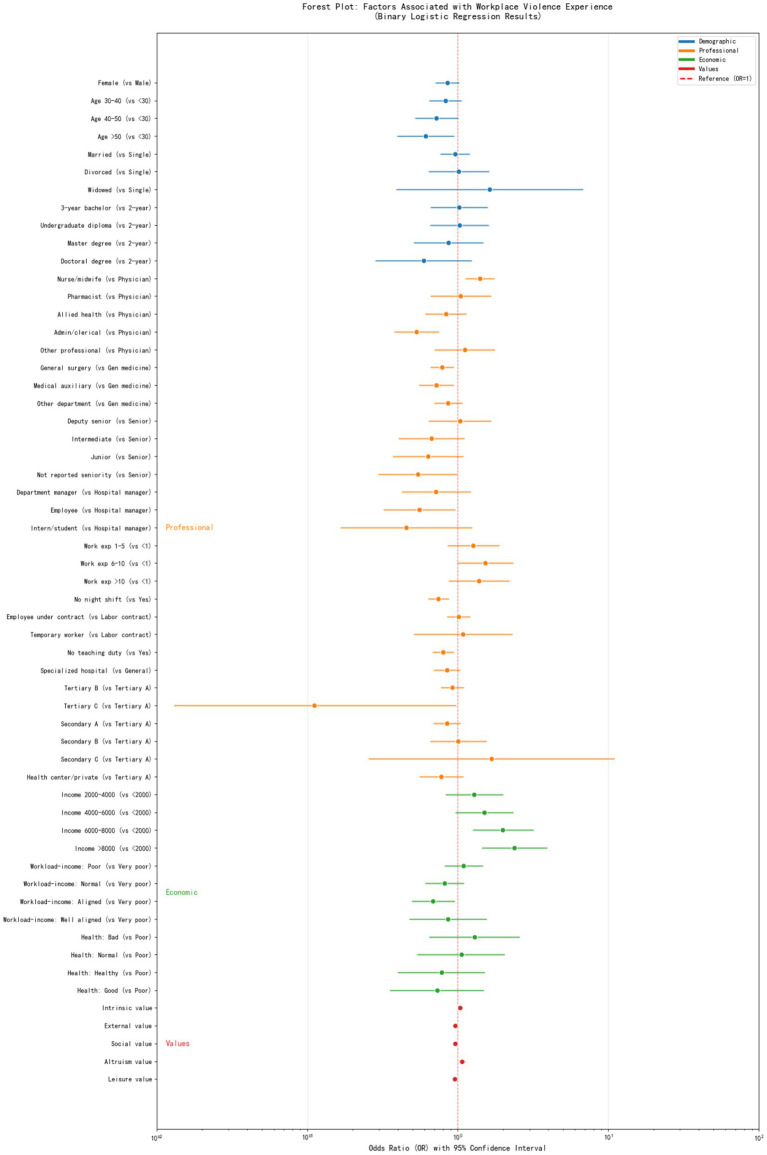
Factors negatively and positively associated with overall WPV (binary).

Table 3 and Figure 3 show the results of binary logistic regression, we coded health professionals who experienced at least one-time WPV event as 1, otherwise 0, and explore the influencers. Binary logistic regression analysis identified several significant independent predictors of WPV among healthcare professionals. After adjusting for all covariates, nurses and midwives had 40.5% higher odds of experiencing workplace violence compared to physicians (OR = 1.405, 95% CI: 1.127–1.751, *p* = 0.003), while administrative or clerical workers had 47.0% lower odds (OR = 0.530, 95% CI: 0.377–0.746, *p* < 0.001). HCWs aged over 50 years had significantly lower odds of violence exposure compared to those under 30 (OR = 0.612, 95% CI: 0.395–0.946, *p* = 0.027). Departmental differences were evident, with general surgery (OR = 0.786, 95% CI: 0.657–0.940, *p* = 0.008) and medical auxiliary departments (OR = 0.719, 95% CI: 0.551–0.938, *p* = 0.015) showing lower violence risk compared to general medicine. Work-related factors negatively associated with WPV included absence of night shift work (OR = 0.744, 95% CI: 0.635–0.871, *p* < 0.001) and no teaching responsibilities (OR = 0.798, 95% CI: 0.679–0.939, *p* = 0.007). Higher monthly income demonstrated a dose–response relationship with increased violence risk: compared to those earning <2,000 CNY, HCWs earning 6,000–8,000 CNY had nearly double the odds (OR = 1.992, 95% CI: 1.251–3.193, *p* = 0.004), while those earning >8,000 CNY had 2.4-fold higher odds (OR = 2.376, 95% CI: 1.450–3.921, *p* < 0.001). Perceived alignment between workload and income showed that those reporting good alignment had 31.4% lower odds of violence compared to those reporting very poor alignment (OR = 0.686, 95% CI: 0.495–0.951, *p* = 0.024). Regarding occupational values, intrinsic value (*β* = 0.036, *p* = 0.010) and altruism value (*β* = 0.063, *p* = 0.015) were associated with increased violence risk, while external value (*β* = −0.041, *p* = 0.009), social value (*β* = −0.039, *p* = 0.002), and leisure value (*β* = −0.05, *p* < 0.001) were factors negatively associated with WPV.

**Table 3:** Binary logistic regression on whether experienced WPV among healthcare workers.

Variables	Workplace violence (0 or 1)				
	β coefficient	SE (standard coefficient)	Odds ratio	95% CI	*p*-value
Gender (ref = Male)					
Female	−0.16	0.093	0.853	0.710–1.023	0.0865
Age (ref = <30)					
30–40	−0.189	0.126	0.828	0.646–1.060	0.1348
40–50	−0.326	0.169	0.722	0.518–1.006	0.0546
> 50	−0.492	0.223	0.612^**^	0.395–0.946	0.0273
Marriage (ref = Single)					
Married	−0.042	0.115	0.959	0.765–1.201	0.7138
Divorced	0.014	0.236	1.015	0.639–1.610	0.953
Widowed	0.484	0.73	1.625	0.388–6.787	0.5076
Education (ref = 2-year associate’s degree)					
3-year bachelor’s degree	0.021	0.222	1.023	0.661–1.579	0.9229
Undergraduate diploma	0.024	0.23	1.027	0.653–1.608	0.9154
Master’s degree	−0.143	0.272	0.869	0.508–1.478	0.5994
Doctoral degree	−0.526	0.376	0.593	0.283–1.236	0.1622
Professional (ref = Physician)					
Nurse/midwife	0.34	0.112	1.405^***^	1.127–1.751	0.0025
Pharmacist	0.043	0.236	1.044	0.658–1.658	0.8542
Allied health professional (therapist/radiographer/assistant)	−0.182	0.16	0.834	0.609–1.141	0.2562
Administrative or clerical worker	−0.634	0.174	0.530^***^	0.377–0.746	0.0003
Other	0.107	0.236	1.114	0.701–1.769	0.6492
Department (ref = General medicine)					
General surgery	−0.241	0.091	0.786^***^	0.657–0.940	0.0084
Medical auxiliary/ancillary	−0.33	0.136	0.719^**^	0.551–0.938	0.015
Other	−0.148	0.112	0.863	0.693–1.074	0.1858
Seniority (ref = Senior)					
Deputy senior	0.032	0.245	1.033	0.639–1.668	0.8973
Intermediate	−0.401	0.256	0.670	0.405–1.105	0.1166
Junior	−0.456	0.276	0.635	0.369–1.088	0.0983
Not reported	−0.613	0.308	0.543^**^	0.296–0.991	0.0467
Position (ref = Hospital manager)					
Department manager	−0.331	0.268	0.717	0.424–1.215	0.2169
Employee	−0.587	0.278	0.555^**^	0.322–0.960	0.0350
Intern/student/trainee	−0.787	0.513	0.455	0.166–1.246	0.1255
Working experience (ref = < 1)					
1–5	0.237	0.201	1.267	0.855–1.88	0.238
6–10	0.418	0.219	1.518	0.989–2.332	0.0563
> 10	0.323	0.239	1.380	0.864–2.206	0.1772
Night shift (ref = Yes)					
No	−0.296	0.081	0.744^***^	0.635–0.871	0.0002
Employee type (ref = Employee under labor contract)					
Employee under contract	0.011	0.09	1.011	0.848–1.206	0.9022
Temporary worker	0.081	0.385	1.079	0.510–2.305	0.8334
Teaching duty (ref = Yes)					
No	−0.225	0.083	0.798^***^	0.679–0.939	0.0065
Type of hospital (ref = General)					
Specialized	−0.167	0.104	0.846	0.69–1.038	0.1084
Level of hospital (ref = Tertiary A)					
Tertiary B	−0.086	0.09	0.918	0.769–1.096	0.3441
Tertiary C	−2.179	1.101	0.111^**^	0.013–0.979	0.0478
Secondary A	−0.165	0.106	0.848	0.689–1.044	0.12
Secondary B	0.008	0.223	1.008	0.652–1.559	0.9714
Secondary C	0.516	0.96	1.677	0.255–11.001	0.5906
Health center/private hospital	−0.25	0.171	0.778	0.556–1.089	0.1439
Average monthly income (ref = < 2000)					
2000–4,000	0.255	0.224	1.287	0.831–2.003	0.2558
4,000–6,000	0.402	0.227	1.494	0.958–2.332	0.0767
6,000–8,000	0.692	0.239	1.992***	1.251–3.193	0.0038
> 8,000	0.869	0.254	2.376***	1.450–3.921	0.0006
Alignment between workload and income (ref = Very poor)					
Poor	0.089	0.15	1.093	0.815–1.466	0.5516
Normal	−0.2	0.151	0.819	0.609–1.101	0.1855
Aligned	−0.377	0.167	0.686**	0.495–0.951	0.0236
Well	−0.151	0.304	0.86	0.474–1.560	0.6192
Health condition (ref = Poor)					
Bad (3–4)	0.25	0.354	1.295	0.642–2.567	0.4801
Normal (5–6)	0.047	0.341	1.057	0.537–2.046	0.8897
Healthy (7–8)	−0.254	0.342	0.782	0.397–1.516	0.4581
Good (9–10)	−0.321	0.367	0.732	0.354–1.488	0.3814
Intrinsic value	0.036	0.014	1.037**	1.009–1.066	0.01
External value	−0.041	0.016	0.960***	0.930–0.990	0.0089
Social value	−0.039	0.012	0.962**	0.940–0.985	0.0015
Altruism value	0.063	0.026	1.065**	1.012–1.121	0.0151
Leisure value	−0.05	0.008	0.952***	0.936–0.967	< 0.0001
N	4267.000				
R^2^	0.627				

^*^p < 0.1, ^**^p < 0.05, ^***^p < 0.01.

### Ordered logit model regression to identify relative factors negatively and positively associated with WPV in overall and each subtype

**Figure 4:**
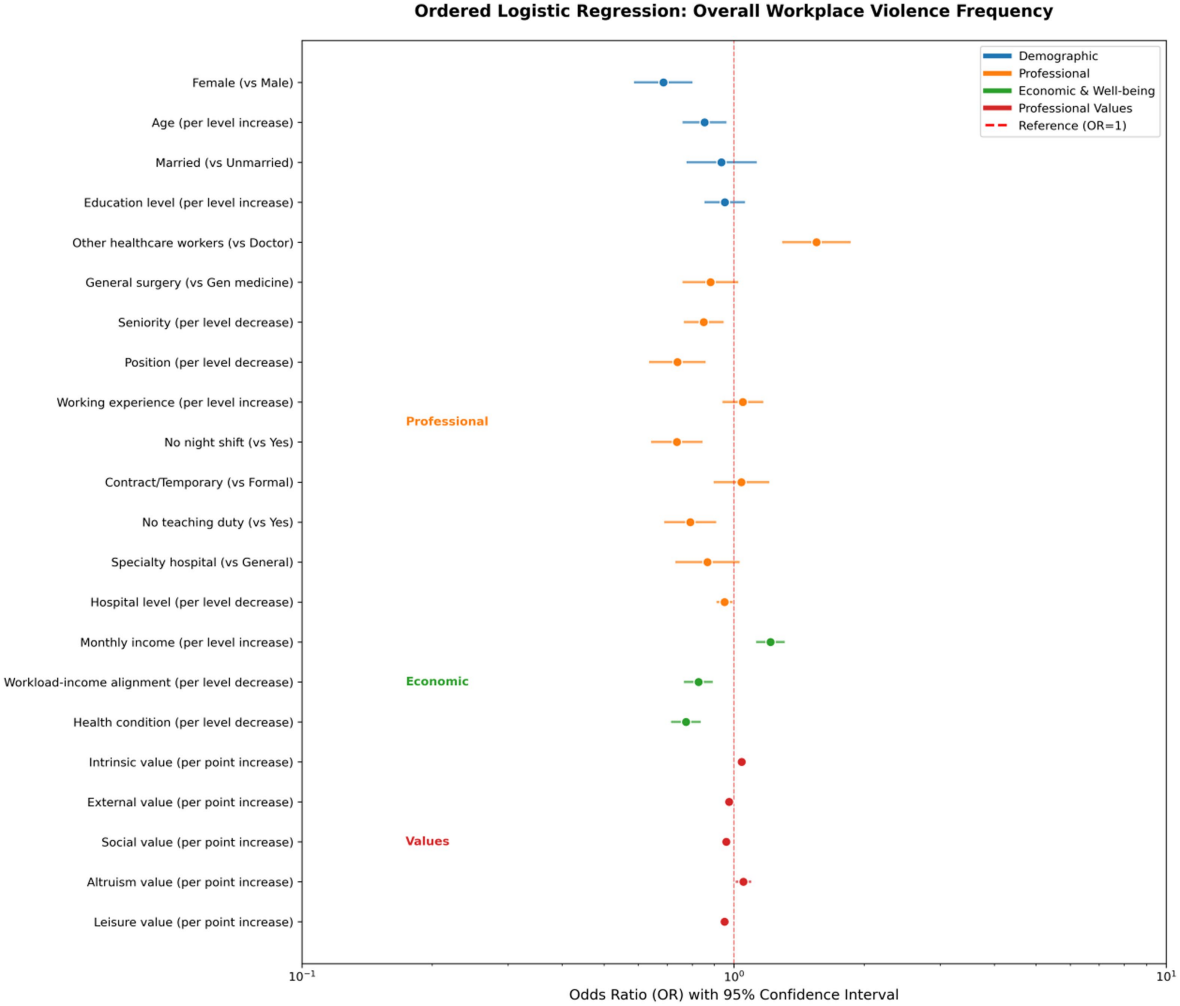
Factors negatively and positively associated with overall WPV (ordered).

The ordered logistic regression analysis presented in Supplementary Table 2 provides valuable insights into various factors associated with WPV experienced by HCWs across five categories: overall workplace violence (Figure 4), physical assault, emotional abuse, threats/intimidation, verbal sexual harassment and physical sexual harassment (Supplementary files).

### Socio-demographic characteristics

Female gender was found to be a significant factor negatively associated with WPV in the ordered logistic models. Across all violence types, with females having 31.9% lower odds of overall WPV (OR = 0.686, 95% CI: 0.587–0.802, *p* < 0.001), 51.9% lower odds of physical assault (OR = 0.481, 95% CI: 0.390–0.594, *p* < 0.001), 21.9% lower odds of emotional abuse (OR = 0.781, 95% CI: 0.668–0.914, *p* < 0.002), 36.2% lower odds of threats intimidation (OR = 0.445, 95% CI: 0.341–0.580, *p* < 0.001), 55.5% lower odds of verbal sexual harassment (OR = 0.445, 95% CI: 0.341–0.580, *p* < 0.001), and 39.3% lower odds of physical sexual harassment (OR = 0.607, 95% CI: 0.434–0.849, *p* = 0.004), compared to males.

Age demonstrated as a factor negatively associated with WPV effect, particularly regarding psychological abuse; for each incremental increase in age level, the odds of experiencing frequent overall WPV decreased by 14.6% (OR = 0.854, 95% CI: 0.760–0.960, *p* = 0.008) and emotional abuse by 16.9% (OR = 0.831, 95% CI: 0.739–0.935, *p* = 0.002).

In contrast, marriage status and education level were not significant predictors for the frequency of any type of workplace violence (*p* > 0.05 for all comparisons).

### Professional and job-related characteristics

Non-physician HCWs demonstrated significantly higher odds across all violence types, having 74.5% higher odds of physical assault (OR = 1.745, 95% CI: 1.356–2.246, *p* < 0.001), 60.6% higher odds of emotional abuse (OR = 1.606, 95% CI: 1.335–1.931, *p* < 0.001), 38.5% higher odds of threats intimidation (OR = 1.385, 95% CI: 1.118–1.719, *p* = 0.003), 63.7% higher odds of verbal sexual harassment (OR = 1.637, 95% CI: 1.189–2.255, *p* = 0.003), and 67.7% higher odds of physical sexual harassment (OR = 1.677, 95% CI: 1.129–2.488, *p* = 0.010), indicating a systemic vulnerability among nursing and support staff.

HCWs in general surgery departments demonstrated a trend toward lower odds of experiencing more frequent WPV compared with their counterparts in general medicine, although this association did not reach conventional statistical significance. Subtype-specific analyses revealed differential patterns across WPV categories. For physical assault, general surgery was associated with 15.3% lower odds of experiencing higher frequency events compared with general medicine (OR = 0.847; 95% CI: 0.697–1.028; *p* = 0.093). Similarly, marginally significant negatively association were observed for emotional abuse (OR = 0.875; 95% CI: 0.755–1.015; *p* = 0.079).

A decrease in seniority level (transitioning towards more junior titles) was independently associated with reduced odds of experiencing frequent violence. The factors are negatively associated with overall WPV (OR = 0.851, 95% CI: 0.766–0.946, *p* = 0.003), physical assault (OR = 0.817, 95% CI: 0.708–0.943, *p* = 0.006), and threats and intimidation (OR = 0.789, 95% CI: 0.697–0.893, *p* < 0.001).

Lower administrative positions were associated with a markedly lower frequency of violence compared to managerial roles. For each level decrease in position (moving away from management), the odds of overall WPV decreased by 26.1% (OR = 0.739, 95% CI: 0.636–0.860, *p* = 0.001). This pattern was robust across physical assault (OR = 0.755, *p* = 0.005), emotional abuse (OR = 0.783, *p* = 0.002), and threats (OR = 0.723, *p* < 0.001). Notably, the strongest factor negatively associated with verbal sexual harassment (OR = 0.687, 95% CI: 0.537–0.878, *p* = 0.003) is lower administrative status, indicating that individuals in higher management positions may face a disproportionately higher burden of aggressive encounters.

For overall WPV frequency, working experience demonstrated no statistically significant association. Subtype-specific analyses revealed heterogeneous patterns across violence categories. Notably, the association between working experience and verbal sexual harassment reached statistical significance (OR = 1.266; 95% CI: 1.043–1.538; *p* = 0.017). This finding indicates that for each incremental level of working experience, healthcare workers had 26.6% higher cumulative odds of reporting more frequent verbal sexual harassment episodes.

HCWs who are at absence of night shift works having reduced odds of overall workplace violence by 26.3% (OR = 0.737, 95% CI: 0.643–0.845, *p* < 0.001), physical assault by 28.6% (OR = 0.714, 95% CI: 0.589–0.865, *p* < 0.001), emotional abuse by 23.1% (OR = 0.769, 95% CI: 0.669–0.882, *p* < 0.001), threats intimidation by 30.8% (OR = 0.692, 95% CI: 0.586–0.816, *p* < 0.001), verbal sexual harassment by 29.8% (OR = 0.702, 95% CI: 0.550–0.897, *p* = 0.005), and physical sexual harassment by 26.6% (OR = 0.734, 95% CI: 0.541–0.997, *p* = 0.0048).

Employment stability, defined by contract status (formal vs. contract/temporary), showing no significant differences between formal employees and contract/temporary workers regarding the odds of overall WPV, physical assault, emotional abuse, or threats (*p* > 0.05). However, a specific vulnerability was identified regarding physical sexual harassment. Contract and temporary workers had significantly higher odds of experiencing frequent physical sexual harassment compared to formal employees (OR = 1.401, 95% CI: 1.019–1.927, *p* = 0.038). This finding highlights a potential safeguarding gap where staff with less secure employment status may be disproportionately targeted for physical sexual misconduct.

Similarly, absence of teaching duties was factors negatively associated with WPV across all violence types except verbal sexual harassment and physical sexual harassment. No teaching duty reduce the odds of overall workplace violence by 20.8% (OR = 0.792, 95% CI: 0.689–0.910, *p* < 0.001), physical assault by 24.8% (OR = 0.752, 95% CI: 0.621–0.911, *p* = 0.004), emotional abuse by 19.2% (OR = 0.808, 95% CI: 0.702–0.929, *p* = 0.003), and threats intimidation by 24.7% (OR = 0.753, 95% CI: 0.638–0.888, *p* < 0.001). These findings suggest that the increased workload and extended hours associated with night shifts and academic responsibilities may compound the risk of exposure to aggression.

The institutional tier of the healthcare facility was identified as a significant environmental predictor for specific types of aggression. A decrease in hospital level (transitioning from tertiary-level centers to secondary or community hospitals) was associated with a modest but significant reduction in the frequency of violence. Specifically, lower hospital levels correlated with reduced odds of overall WPV (OR = 0.951, 95% CI: 0.911–0.992, *p* = 0.019), physical assault (OR = 0.936, *p* = 0.030), and threats and intimidation (OR = 0.922, *p* = 0.002). No significant association was found between hospital level and the frequency of emotional abuse or sexual harassment, suggesting that while physical safety risks may be concentrated in high-level tertiary centers, psychological and sexual aggression risks are more uniformly distributed across the healthcare system. When comparing facility types, the distinction between general and specialized hospitals showed limited prognostic value for most forms of violence. There were no statistically significant differences between general and specialized hospitals regarding the frequency of overall WPV, physical assault, threats, or sexual harassment. The notable exception was emotional abuse; staff working in specialized hospitals had 16.5% lower odds of experiencing frequent emotional abuse compared to their counterparts in general hospitals (OR = 0.835, 95% CI: 0.702–0.994, *p* = 0.042). This suggests that the broader patient mix and high-stress environment of general hospitals may predispose staff to higher levels of verbal and psychological abuse.

### Economic and well-being factors

Higher average monthly income was significantly associated with increased odds of frequent emotional abuse (OR = 1.284, 95% CI: 1.189–1.386, *p* < 0.001) and threats/intimidation (OR = 1.139, 95% CI: 1.042–1.244, *p* = 0.004). However, income level showed no significant association with the frequency of physical assault or either form of sexual harassment.

In contrast to absolute income, the perceived fairness of compensation, measured as the alignment between workload and income, served as a universal factor negatively associated with WPV. Specifically, for each level of improvement in perceived income alignment, the odds of experiencing overall WPV decreased by 17.3% (OR = 0.827, 95% CI: 0.766–0.893, *p* < 0.001), physical assault by 14.4% (OR = 0.856, 95% CI: 0.773–0.947, *p* = 0.003), emotional abuse by 16% (OR = 0.840, 95% CI: 0.779–0.907, *p* < 0.001), threats intimidation by 18.2% (OR = 0.818, 95% CI: 0.749–0.893, p < 0.001), verbal sexual harassment by 24.2% (OR = 0.758, 95% CI: 0.668–0.860, *p* = 0.005), and physical sexual harassment by 18% (OR = 0.820, 95% CI: 0.701–0.960, *p* = 0.013). This finding underscores that the perception of equitable compensation may be more critical for workforce safety than income magnitude alone.

Self-rated health condition demonstrated strong, consistent dose–response relationships with all forms of WPV. Poorer health conditions were significantly associated with increased violence frequency. Specifically, the model indicated that for every unit decrease in health status, the odds of experiencing higher frequencies of violence increased significantly, for overall WPV (OR = 0.774, 95% CI: 0.715–0.838, *p* < 0.001), physical assault (OR = 0.739, 95% CI: 0.667–0.818, *p* < 0.001), emotional abuse (OR = 0.795, 95% CI: 0.734–0.860, *p* < 0.001), threats intimidation (OR = 0.753, 95% CI: 0.687–0.824, *p* < 0.001), verbal sexual harassment (OR = 0.804, 95% CI: 0.708–0.913, *p* < 0.001), and physical sexual harassment (OR = 0.751, 95% CI: 0.641–0.880, *p* < 0.001).

### Professional values

Intrinsic value demonstrated a significant positive association with overall WPV frequency (OR = 1.042; 95% CI: 1.018–1.066; *p* < 0.001), indicating that each one-point increase in intrinsic value score was associated with 4.2% higher odds of experiencing more frequent violence. This association was most pronounced for emotional abuse (OR = 1.054; 95% CI: 1.030–1.080; *p* < 0.001) and threats/intimidation (OR = 1.033; 95% CI: 1.005–1.060; *p* = 0.020).

External value exhibited a marginally significant inverse association with overall WPV frequency (OR = 0.974; 95% CI: 0.950–1.000; *p* = 0.054). Higher external value scores are significantly negatively associated with emotional abuse. Each one-point increase in external value corresponded to 4.7% lower odds of more frequent emotional abuse (OR = 0.953; 95% CI: 0.928–0.978; p < 0.001). Conversely, external value demonstrated significant positive associations with both verbal sexual harassment (OR = 1.063; 95% CI: 1.018–1.111; *p* = 0.006) and physical sexual harassment (OR = 1.065; 95% CI: 1.008–1.125; *p* = 0.025), representing 6.3 and 6.5% increased odds per unit increment, respectively.

Social value is consistently negatively associated with WPV across subtypes. For overall WPV, each one-point increase in social value was associated with 4.1% lower odds of experiencing higher-frequency violence (OR = 0.959; 95% CI: 0.940–0.978; *p* < 0.001). Significant factors negatively associated with WPV effects were similarly observed for emotional abuse (OR = 0.958; 95% CI: 0.938–0.977; *p* < 0.001) and threats/intimidation (OR = 0.976; 95% CI: 0.954–0.999; *p* = 0.039).

Altruism value was significantly associated with increased frequency of overall WPV (OR = 1.051; 95% CI: 1.008–1.096; *p* = 0.020) and emotional abuse (OR = 1.080; 95% CI: 1.035–1.126; *p* < 0.001). The latter association indicated that each one-point increase in altruism value corresponded to 8.0% higher cumulative odds of reporting more frequent emotional abuse episodes.

Leisure value is negatively associated with WPV across all subtypes. Each one-point increase in leisure value was associated with significantly lower odds of experiencing more frequent overall WPV (OR = 0.951; 95% CI: 0.938–0.965; *p* < 0.001), physical assault (OR = 0.970; 95% CI: 0.953–0.989; *p* = 0.002), emotional abuse (OR = 0.949; 95% CI: 0.936–0.963; *p* < 0.001), threats/intimidation (OR = 0.957; 95% CI: 0.941–0.973; *p* < 0.001), and verbal sexual harassment (OR = 0.976; 95% CI: 0.954–0.999; *p* = 0.041). Only physical sexual harassment showed no significant association with leisure value.

## Discussion

### Summary of key findings

Our binary logistic regression analysis identified several factors significantly positively associated with overall WPV, including nursing profession, higher income levels, higher scores of intrinsic value and altruism values. Factors negatively associated with WPV included older age (>50 years), administrative positions, working in general surgery or medical auxiliary departments, being employee, no night shift work, no teaching duty, working in Tertiary C hospitals, perceived alignment between workload and income, and higher scores of external values, social value, as well as leisure value. The ordered logistic regression analysis extends the understanding of WPV beyond binary occurrence by examining factors associated with violence frequency across a gradient of exposure (never, once, 2–3 times, ≥4 times). This analytical approach permits identification of factors that not only predict WPV occurrence but also predict escalation toward repeated victimization, a critical distinction for targeted intervention development. Furthermore, the subtype specific analyses reveal distinct risk profiles for physical assault, emotional abuse, threats intimidation, verbal sexual harassment, and physical sexual harassment, highlighting that WPV represents a heterogeneous phenomenon requiring differentiated prevention strategies. Better health status, better workload-income alignment, absence of night shifts, and female gender demonstrated consistent protection across most or all violence types, suggesting these represent fundamental determinants of WPV vulnerability requiring priority intervention. Several factors (age, income, intrinsic value, altruism value) specifically affected verbal violence (emotional abuse, threats) without significant effects on physical violence, suggesting distinct pathways for these violence types. The ordered logistic regression also found that intrinsic motivation and altruism, traditionally valued professional attributes, are associated with increased WPV represents a critical insight for occupational health interventions. While these values should continue to be cultivated, training programs should simultaneously equip HCWs with boundary-setting skills and sustainable compassion practices. External value orientation emerging as the factor solely positively associated with sexual harassment, suggesting it may operate through distinct mechanisms requiring specialized prevention approaches.

### The burden of WPV among Chinese healthcare workers

Regardless of types, among the 4,267 healthcare professionals surveyed, the prevalence of WPV in our study is 57.6%, which is higher to most reported rates in other large-scale studies (25 to 50%) investigating WPV against HCWs (28, 58). Compared to study in China, our results reveal that WPV prevalence was decreased by nearly 10% (59). Among the five types of WPV, emotional abuse was the most prevalent form, affecting 54.7% (n = 2,294) of participants, then threats and intimidation (30.5%, n = 1,303), physical assault (20.2%, *n* = 860), verbal sexual harassment (11.5%, *n* = 489), and physical sexual harassment (7.0%, *n* = 297). Our findings both align with and diverge from previous research (60, 61). The frequencies of different types of WPV in our study were also higher than those in previous surveys of HCWs, and previous studies reporting a wide range in prevalence of WPV against HCWs, owing to significant population and methodological differences (15, 48, 62–65). Although data synthesis was complicated by heterogeneity and subjective reporting, oral violence was the main trend, however, we found that emotional abuse was the most prevalent type of WPV.

Notably, repeated WPV was common, with 35.0% (*n* = 1,495) of participants experiencing emotional abuse two or more times, 14.48% (*n* = 618) experiencing threats and intimidation multiple times, and 9.4% (*n* = 401) experiencing physical assault on multiple occasions, 5.3% (*n* = 227) of participants experiencing verbally sexual harassment two or more times, and 2.7% (*n* = 115) experiencing physically sexual harassment sexual harassment two or more times. Our results revealing that not just the occurrence but also the repeated harms persist in the medical setting (66–68). Therefore, comparing frequencies of WPV across studies must be done with care.

We conclude, at the very least, that violence in medical workplaces was in a decreased trend in China but is still at the moderate level, and HCWs are not only exposed to single incidents of WPV but also face a severe prevalence of recurrent WPV incidents in medical settings.

### The paradox of professional engagement

Perhaps the most intriguing finding of this study concerns the paradoxical relationship between professional values and WPV risk. Intrinsic value orientation and altruism value orientation were both independently associated with increased WPV exposure. HCWs with strong intrinsic motivation and altruistic values may demonstrate greater patient engagement, spending more time in direct patient interaction, investing more emotional energy in patient care, and going beyond standard protocols to address patient needs (69, 70). While these behaviors reflect professional excellence, they paradoxically increase exposure opportunities to potentially violent individuals. Highly altruistic HCWs may have difficulty establishing firm professional boundaries, potentially allowing inappropriate patient behaviors to escalate before implementing necessary limits (71, 72). Their commitment to patient welfare may lead them to tolerate initial aggressive behaviors, inadvertently reinforcing such conduct. Intrinsically motivated HCWs engage in substantial emotional labor, the effortful regulation of emotions to meet professional expectations. Sustained emotional labor depletes psychological resources, potentially compromising their capacity for effective de-escalation during tense encounters and increasing their vulnerability to perceiving situations as violent.

In striking contrast to the risk-conferring effects of intrinsic and altruistic values, external value orientation, social value orientation, and leisure value orientation were all independently factors negatively associated with WPV. HCWs with external, social, and leisure value orientations may maintain clearer psychological boundaries between their professional and personal identities. This separation may enable more objective responses to patient aggression, reducing emotional reactivity and conflict escalation (73). While potentially viewed negatively from a humanistic care perspective, some degree of emotional detachment may serve a factor negatively associated with WPV function (74). HCWs who view their work instrumentally (external values) or prioritize life domains outside work (leisure values) may be less emotionally invested in individual patient encounters, reducing vulnerability to violent incidents and moderating their reporting of such incidents. Social support networks are also important in previous studies: HCWs with strong social value orientations may have developed more robust collegial relationships and social support networks, providing resources for managing occupational stress and potentially creating environments where colleagues intervene to prevent violence escalation (75). Work-life balance is a significant factor negatively associated with WPV. HCWs who prioritize rest and recreation may experience less cumulative fatigue and burnout, preserving psychological resources for effective patient management (76). Our findings align with and extend the Conservation of Resources (COR) theory, HCWs with strong intrinsic and altruistic values may invest greater personal resources in patient care, depleting their resource reservoirs and increasing vulnerability to WPV (77). Conversely, workers with external, social, and leisure orientations may practice more effective resource conservation, maintaining reserves that buffer against occupational stressors including violence (78). This paradox can be understood factors that increase HCWs’ clinical engagement simultaneously increase their exposure to potentially violent situations. The most dedicated, accomplished, and centrally positioned HCWs face the greatest WPV risk precisely because their commitment places them at the interface of healthcare delivery where patient distress, family anxiety, and system failures converge. Our study contributes new insights into how intrinsic psychological traits influence safety. We observed that professionals driven by altruism and intrinsic values were more likely to experience more frequent violence. This counterintuitive finding suggests that highly empathetic and self-sacrificing individuals may be more emotionally invested in patient outcomes, potentially leading to boundary-blurring or over-engagement that, paradoxically, can escalate into conflict when patient demands are unmet. In contrast, those valuing leisure and social aspects showed reduced risk. A focus on work-life balance may foster better stress management and emotional detachment, acting as WPV buffer against the toxicity of aggressive encounters (79).

### Economic factors: the divergence between income and fairness

The regression analysis also revealed a striking dose–response relationship between income and WPV risk. HCWs earning 6,000–8,000 CNY monthly faced nearly double the WPV risk compared to those earning <2,000 CNY, while those earning >8,000 CNY demonstrated even higher risk. Higher-earning HCWs may manage more complex cases, perform higher-risk procedures, or bear greater responsibility for adverse outcomes, situations inherently associated with patient and family dissatisfaction. Higher-income HCWs in the Chinese context often practice at prestigious tertiary hospitals where patients harbor elevated expectations for treatment outcomes (26). The psychological phenomenon of relative deprivation suggests that patients investing more resources (financial, temporal, emotional) in their healthcare may experience greater frustration when outcomes fail to meet expectations, potentially manifesting as violence.

HCWs perceiving good alignment between workload and income demonstrated significantly lower WPV risk compared to those perceiving very poor alignment. Perceived fairness in compensation contributes to a sense of organizational justice, which serves as a psychological resource buffering against occupational stressors. HCWs who feel fairly compensated may approach patient interactions with greater psychological resilience (80). And the perceived fairness protects against burnout, preserving HCWs’ emotional resources for effective patient management (81). Ordered logistic results show that better perceived alignment between workload and income demonstrated significant negatively association with WPV and across all WPV types. This finding extends the organizational justice literature to WPV outcomes.

A striking and novel finding of this study is the divergence between absolute income and perceived compensation fairness. Paradoxically, higher absolute monthly income was strongly associated with an increased risk of WPV. This likely reflects the high-pressure environments occupied by high earning professionals (often senior clinicians handling complex, high stakes cases) where patient expectations and consequent frustrations are elevated. However, our finding regarding higher income as a factor positively associated with WPV diverges from most literature. Zhao et al. found that lower income was associated with higher violence exposure among HCWs (26). This discrepancy may reflect unique aspects, where higher-paid professionals may have greater patient contact or responsibility in contentious situations. However, this risk appears mitigatable by organizational justice (82). We found that the perception of alignment between workload and income served as a significant factor negatively associated with WPV. This suggests that when professionals feel their efforts are rewarded, they may exhibit greater job satisfaction and psychological resilience, potentially influencing their interpersonal dynamics with patients or their threshold for reporting aggression (83, 84). This underscores that WPV prevention requires not just security measures, but also transparent and equitable compensation policies.

### The vulnerability of high workload healthcare workers

Our study also found that HCWs without night shift duties demonstrated significantly lower WPV risk. Possible reasons are that night shifts typically operate with reduced staffing levels, limiting both the capacity to manage aggressive patients and the availability of supervisory support during escalating situations (27). Both patients and HCWs may experience circadian rhythm disruption during nighttime hours, potentially affecting judgment, impulse control, and communication effectiveness on both sides of the patient-provider relationship (85). HCWs without teaching duties demonstrated reduced WPV risk. Teaching hospitals in China are predominantly tertiary institutions with high patient volumes and complex cases, and teaching responsibilities may mark positions within particularly demanding clinical environments (86). Patients may perceive care involving trainees as lower quality or may feel uncomfortable being “practice cases,” potentially increasing dissatisfaction and conflict risk. Besides, teaching responsibilities create competing demands on HCWs’ time and attention. The cognitive load of simultaneously providing clinical care, supervising trainees, and meeting educational obligations may compromise the quality of patient communication and reduce capacity for effective conflict management (86). The negative association between “no night shifts”, “no teaching duties” and WPV highlight the compounding impact of fatigue and role conflict, and the results are similar to studies in Hong Kong and South Korea (87, 88). Night shifts often involve reduced staffing and heightened patient anxiety, creating a perfect storm for aggression, while teaching duties add cognitive load, potentially reducing the emotional bandwidth available for de-escalating patient conflicts. Our identification of night shifts as a factor positively associated with WPV aligns with another study of Chinese hospitals, which found that working night shifts increased violence risk by 49% (27). Similar to binary results, the absence of night shift work emerged as a factors negatively associated with WPV and across all WPV types. While the absence of teaching duties is negatively associated with all types of WPV except verbal sexual harassment and physical sexual harassment.

### Sociodemographic risk profiles: challenging conventional assumptions

In binary regression analysis, gender differences were not statistically significant, suggesting that gender does not influence the likelihood of experiencing WPV, and our results was significant higher than previous meta-analysis (reporting a pooled prevalence of 50.8% in a miscellaneous of HCWs) (48). However, ordered logistic regression revealed a more nuanced pattern: female gender emerged as a consistent factor negatively associated with repeated WPV exposure, with subtype-specific effect magnitudes showing substantial variation. This discrepancy indicates that while male and female HCWs have similar probabilities of any WPV exposure, male HCWs face higher risk of experiencing repeated or more frequent violent incidents. For overall WPV, female HCWs demonstrated 31.4% lower odds of experiencing more frequent violence compared to males. This negatively association was most pronounced for physical assault, representing a 51.9% reduction in odds of higher-frequency physical violence. Similarly strong negatively association were observed for verbal sexual harassment and threats/intimidation. More modest but significant protection was evident for emotional abuse and physical sexual harassment. This finding presents a notable departure from the Western literature, where female HCWs are typically identified as being at higher risk for repeated WPV, particularly sexual harassment (89). In Chinese healthcare settings, male HCWs are disproportionately assigned to high-acuity clinical positions, emergency departments, and situations requiring physical patient management (90). These assignments mechanically increase their exposure to potentially violent patients. Moreover, disparity in WPV subtypes revealing that perpetrators of WPV may perceive male HCWs as more acceptable targets for physical aggression due to cultural norms discouraging violence against women (91, 92). This selection effect would explain the particularly strong negative association between female gender and physical assault compared to emotional abuse. The divergence between binary and ordered regression results suggests that gender influences not whether WPV occurs, but rather its frequency and escalation patterns. Male HCWs may be more likely to experience repeated incidents and more severe forms of violence.

In binary regression, HCWs aged >50 years demonstrated significantly reduced WPV risk compared to those <30 years. Developmental psychology research consistently demonstrates improved emotional regulation with advancing age, a phenomenon termed the “positivity effect” (93). Older HCWs may be better equipped to manage their emotional responses during tense patient encounters, preventing escalation. By accumulating broader life experience, their interpersonal skills, including recognizing early warning signs of escalating aggression and implementing proactive de-escalation strategies may also increase (94), reflecting that younger HCWs lack effective communication skills, placing them at greater risk of verbal abuse or bullying (95). Selective survival effect also can explain these negative association, those who suffered WPV may have left the profession or transitioned to lower-risk positions, creating a survivor bias in the older cohort. Moreover, our results show that advancing age is a factor significant negatively associated with overall WPV frequency and emotional abuse in ordered logistic regression. The OR of 0.831 indicates that for each one-unit increase in age category, HCWs have 16.9% lower odds of experiencing higher-frequency WPV events. This factor is significant negatively associated with WPV, specifically for emotional abuse, while no significant associations were observed for physical assault or sexual harassment. This pattern suggests that the negative association of age and WPV operates primarily through mechanisms relevant to verbal and emotional violence rather than physical or sexual violence. The negative association between emotional abuse and age aligns with findings demonstrated that older HCWs possessed more sophisticated emotional regulation capabilities and de-escalation skills (96). However, we found that age does not protect against physical violence contrasts with the meta-analysis which identified age as protective factor across all violence types (23). This discrepancy may reflect context-specific factors in Chinese healthcare settings. Older HCWs have developed refined communication skills through decades of patient interaction, these skills are particularly relevant for preventing emotional abuse, which typically evolves through a verbal escalation pathway. The contrast between binary and ordered regression results provides important nuance: while older age reduces the likelihood of any WPV exposure (binary model), its negative association with WPV in the frequency of violence (ordered model) is primarily confined to emotional abuse. This suggests that age-related competencies, particularly emotional regulation and verbal de-escalation, are effective for preventing repeated or escalating emotional violence but offer limited protection against physical or sexual violence subtypes.

### Health status: cause or consequence

The ordered logistic regression analysis revealed that self-reported health condition was one of the most robust and consistent predictors of WPV frequency across all violence subtypes. For each level improvement in health status, HCWs demonstrated significantly reduced odds of experiencing more frequent overall WPV. This negative association was remarkably consistent across all violence subtypes: physical assault, emotional abuse, threats and intimidation, verbal sexual harassment, and physical sexual harassment. Notably, the negative association was strongest for physical assault and physical sexual harassment, suggesting that health status may be particularly relevant for preventing physical forms of violence. However, the relationship between health condition and WPV likely operates through bidirectional mechanisms (97). First, healthier HCWs possess greater physical and psychological resources for managing challenging patient encounters. Adequate physical stamina enables sustained attention and patience during prolonged difficult interactions, while psychological resilience facilitates effective de-escalation before situations progress to violence (98). This interpretation aligns with the Conservation of Resources theory, which posits that individuals with depleted resource reservoirs are more vulnerable to occupational stressors, including interpersonal conflict (99). Second, the cross-sectional nature of this study cannot exclude reverse causation, whereby repeated WPV exposure directly impairs healthcare worker health. This bidirectional relationship creates a potentially reinforcing cycle wherein initial health vulnerability increases WPV exposure, which further compromises health, perpetuating increased vulnerability.

### Professional and occupational vulnerability

Our study reveals the professional and job-related characteristics vulnerability in the background of China hospitals. After controlling for confounding variables, nurses and midwives demonstrated significantly elevated WPV risk compared to physicians. This finding diverges from the crude prevalence analysis, where physicians showed higher raw WPV rates (65.8% vs. 59.0%). Our results were similar to previous studies, highlighting the severe victim position of nurses and physicians (100, 101). Several mechanisms may explain this elevated risk: nurses typically spend substantially more time in direct patient contact than physicians, providing continuous bedside care, administering medications, and assisting with activities of daily living. This sustained proximity increases exposure opportunities to potentially violent patients and family members; the nursing profession in China remains predominantly female, and research suggests that female HCWs may be perceived as more approachable targets for aggression (30). Additionally, nurses may occupy positions of lower perceived authority compared to physicians, potentially emboldening aggressive behavior from patients who perceive power imbalances (2); nurses often serve as communication intermediaries between physicians and patients, sometimes required to explain or defend medical decisions they did not make, placing them in vulnerable positions when patients are dissatisfied with their care (19); and they frequently serve as frontline enforcers of hospital policies that patients may find frustrating, including visiting hour restrictions, dietary limitations, and medication schedules, exposing them to displaced patient anger. Administrative and clerical workers demonstrated dramatically reduced WPV risk compared to physicians. Similarly, employees in non-managerial positions showed lower risk than hospital managers. Administrative workers have substantially less direct patient contact, mechanically reducing their exposure to potentially violent patients (102). Physical and organizational barriers also help administrative personnel, areas physically separated them from clinical spaces, with access controlled by organizational protocols, providing structural protection from violent encounters. In ordered logistic regression, other HCWs (nurses, pharmacists, allied health professionals, and administrative staff combined) demonstrated significantly elevated odds of experiencing more frequent WPV across all violence types compared to physicians. For overall WPV, non-physician HCWs showed 55.1% higher odds of more frequent exposure. This elevated risk was most pronounced for physical assault, followed by physical sexual harassment, verbal sexual harassment, emotional abuse, and threats/intimidation. Our subtype-specific analysis adds important nuances, demonstrating that the nursing profession’s vulnerability is particularly pronounced for physical forms of violence (physical assault OR = 1.745; physical sexual harassment OR = 1.677). Nurses and allied health professionals spend substantially more time in close physical proximity to patients than physicians. Nursing activities, bathing, toileting, repositioning, wound care, require intimate physical contact that provides opportunities for both intentional and disinhibited violent behavior, explaining the particularly elevated risk for physical assault and sexual harassment subtypes.

General surgery and medical auxiliary departments demonstrated significantly lower cumulative WPV risk compared to general medicine departments. Internal medicine frequently manages chronic, progressive, and terminal conditions where cure is not possible (23). Patients and families facing such diagnoses may direct their frustration, grief, and anger toward available HCWs. Surgical patients often have more discrete, treatable conditions with clearer outcome expectations (103). While medical auxiliary departments typically involve single, brief encounters for specific procedures, limiting relationship development but also limiting exposure time (104). Internal medicine, conversely, often involves prolonged hospitalizations with repeated interactions throughout disease progression.

Lower professional seniority demonstrated significant negative association with overall WPV, physical assault, threats/intimidation, verbal sexual harassment, and physical sexual harassment. Notably, seniority was not significantly associated with emotional abuse frequency. This finding indicates that junior HCWs experience less frequent WPV than senior colleagues after adjusting for other variables. The lack of association with emotional abuse suggests that verbal conflict is distributed more equally across seniority levels, while physical violence and sexual harassment preferentially target senior staff.

The finding that ordinary employees are safer than managers is notable; it implies that in the Chinese healthcare context, hospital or department managers often bear the brunt of escalated disputes, serving as the final repository for unresolved patient grievances. These results are consistent with studies of workplace frequency in nurses around the world (25, 105). Front-line clinical workers such as physicians or nurses may be at greater risk of violence than allied health professionals or administrative staff because they lead and manage overall patient care (106). Lower position level demonstrated significant negative association in overall WPV, emotional abuse, threats/intimidation, physical assault, and verbal sexual harassment. Physical sexual harassment showed a similar trend but did not reach statistical significance. Managers are frequently called upon to resolve escalating patient conflicts, placing them in direct confrontation with already agitated individuals. Non-managerial employees may be able to defer to supervisors when situations escalate, transferring violence risk upward. And these results can be seen as robust evidence for seniority, since most managers usually have higher levels of seniority.

Based on the combined results of the binary logistic regression and ordered logistic regression analyses, the impact of working experience on workplace violence among healthcare workers showed a complex pattern: in the binary analysis of whether violence had been experienced, the group with 6–10 years of work experience exhibited a borderline significantly higher risk; in the ordered analysis of violence frequency, working experience had a significant positive effect only on the frequency of verbal sexual harassment. These findings suggest that workplace violence prevention strategies should be tailored according to healthcare workers’ career stages and the specific type of violence, with particular attention to the vulnerability of mid-career staff and the cumulative risk of exposure to sexual harassment.

### Institutional characteristics

The binary found both logistic regression analysis as well as ordered logistic regression results showing that marital status and education had no statistically significant association with WPV. Ordered logistic regression found that working in lower-level hospitals demonstrated negative associations with overall WPV, threats/intimidation, and physical assault. Tertiary hospitals concentrate the most complex cases, receiving referrals from lower-level facilities when local management fails. Patients presenting at tertiary hospitals often have exhausted other options and may harbor heightened frustration from prolonged illness or failed prior treatments (107). Patients and families may hold unrealistically high expectations for tertiary hospitals, perceiving them as institutions capable of solving any medical problem. When outcomes fail to meet these elevated expectations, the discrepancy generates frustration that may manifest as physical violence or threats.

## Management suggestions

Based on the empirical findings of this multi-center study, we propose evidence-based management recommendations stratified across individual, departmental, institutional, and policy levels. These suggestions aim to translate our research findings into actionable interventions for reducing WPV among HCWs in China.

### Targeted training programs for high-risk groups

Our findings identify nurses, night shift workers, internal medicine staff, department managers, and HCWs with teaching responsibilities as populations at elevated WPV risk. Healthcare institutions should implement prioritized, role-specific violence prevention training for these groups. Training curricula should encompass early recognition of escalating aggression, verbal de-escalation techniques, safe physical intervention strategies, and post-incident psychological first aid. Given that mid-career HCWs demonstrated borderline elevated risk, specialized training modules addressing the unique challenges faced during this career phase, including increased clinical autonomy without commensurate authority, should be developed. Furthermore, the significant association between working experience and verbal sexual harassment frequency necessitates the integration of sexual harassment prevention and response training throughout the professional lifespan.

### Workload optimization and schedule management

The consistent negative association between WPV and absence of night shift work and teaching duties underscore the critical importance of workload management. Hospital administrators should consider implementing evidence-based scheduling practices, including: (a) reducing mandatory night shift frequency through flexible roster systems; (b) ensuring adequate staffing levels during night hours to prevent situations where HCWs work alone; (c) implementing recovery time policies following night shifts to mitigate cumulative fatigue; and (d) providing protected non-clinical time for HCWs with teaching responsibilities to reduce role conflict. Additionally, tertiary hospitals, which demonstrated elevated WPV risk, should consider enhanced security protocols during high-risk periods, particularly night shifts and peak patient flow times.

### Compensation equity and organizational justice

A striking finding of this study is the divergent effects of absolute income (factors positively associated with WPV) and perceived workload-income alignment (factors negatively associated with WPV). This paradox suggests that WPV prevention requires not merely security measures but also transparent and equitable compensation policies. Healthcare institutions should: (a) conduct regular assessments of perceived compensation fairness across departments and professional groups; (b) implement transparent workload measurement systems that inform compensation decisions; (c) establish formal mechanisms for addressing compensation grievances; and (d) communicate clearly how workload translates to compensation. When HCWs perceive their efforts as fairly rewarded, they may exhibit greater psychological resilience and more positive interpersonal dynamics with patients, thereby reducing conflict escalation.

### Professional values integration and boundary training

Perhaps the most novel and counterintuitive finding concerns the double-edged sword effect of professional values. While intrinsic motivation and altruism are essential to healthcare excellence, they paradoxically increased WPV risk. Conversely, external values, social values, and leisure values demonstrated negative associations with WPV. Rather than discouraging dedication, training programs should equip HCWs with skills to practice sustainable compassion, maintaining empathetic patient care while establishing healthy psychological boundaries. Specific interventions should include: (a) boundary-setting skills training that enables HCWs to recognize when patient demands exceed professional scope; (b) emotional regulation workshops teaching cognitive reappraisal techniques for managing patient aggression without depleting psychological resources; (c) mindfulness-based stress reduction programs to prevent emotional exhaustion; and (d) peer support systems encouraging work-life balance without stigmatizing leisure orientation.

### Age-integrated mentorship programs

The significant negative association between WPV and older age likely reflects accumulated de-escalation expertise, emotional regulation skills, and interpersonal competencies. Healthcare institutions should leverage this expertise through formal mentorship programs pairing senior HCWs with younger colleagues. Such programs should facilitate: (a) structured observation of conflict management by experienced practitioners; (b) debriefing sessions following violent incidents where mentors guide reflection; (c) role-playing exercises for practicing de-escalation techniques; and (d) knowledge transfer regarding early warning sign recognition. This intergenerational approach transforms organizational experience into institutional knowledge, addressing the vulnerability of younger HCWs while preserving valuable expertise.

### Department-specific safety protocols

The elevated WPV risk in general medicine departments compared to surgical and auxiliary departments necessitates department-specific safety protocols. Internal medicine units should implement: (a) enhanced communication training addressing the emotional challenges of chronic, progressive, and terminal illness management; (b) family conference protocols for high-conflict situations; (c) environmental modifications (e.g., secure nursing stations, panic buttons, camera surveillance); and (d) multidisciplinary violence risk assessment tools integrated into admission procedures. Additionally, specialized hospitals demonstrated negative association with emotional abuse, suggesting that general hospitals with diverse patient populations require broader-spectrum violence prevention approaches.

### Staff wellness and health promotion

The robust negative association between better self-rated health condition and all WPV subtypes highlights the critical link between HCW wellness and workplace safety. Institutions should invest in comprehensive wellness programs including: (a) routine health surveillance with early intervention for declining health indicators; (b) mental health resources including confidential counseling services; (c) physical fitness facilities and programs; (d) nutritional support for shift workers; and (e) recovery resources following violent incidents. The bidirectional relationship between health and WPV exposure suggests that wellness promotion may interrupt a vicious cycle wherein poor health increases vulnerability, leading to WPV exposure, which further compromises health.

### Employment security and contractual protections

The elevated risk of physical sexual harassment among contract and temporary workers reveals a safeguarding gap requiring targeted intervention. Institutions should: (a) ensure equal access to violence prevention training regardless of employment status; (b) establish clear reporting mechanisms that protect non-permanent staff from retaliation; (c) review employment policies to reduce power imbalances that may enable predatory behavior; and (d) consider the long-term benefits of employment stability for workforce safety.

### Study limitations

Several limitations warrant consideration when interpreting our findings. First, the cross-sectional design precludes causal inference; the observed associations between professional values and WPV may be bidirectional, with violence exposure potentially influencing professional values over time. Longitudinal studies are needed to establish temporal precedence. Second, WPV was assessed through self-report, introducing potential recall bias and subjective interpretation of violent incidents. Cultural norms regarding violence reporting in China may have influenced disclosure patterns differently across demographic groups. Third, although we employed multi-stage stratified sampling, certain hospital strata (particularly Tertiary C and Secondary C hospitals) had limited representation, potentially affecting generalizability to these settings. Our findings should be interpreted with particular caution for these underrepresented categories. Fourth, the professional values scale, while validated in Chinese healthcare populations, may not fully capture culturally specific value dimensions relevant to WPV in the Chinese context. Fifth, we did not employ path analysis or structural equation modeling. Future research should utilize these methods to examine mediating pathways between professional values and WPV. Finally, our study focused on patient/visitor-perpetrated violence and did not assess lateral violence among colleagues, which represents another significant occupational hazard for HCWs.

## Conclusion

This study systematically reveals the occurrence patterns and influencing factors of WPV among Chinese HCWs by innovatively employing a dual analytical framework of binary logistic regression and ordinal logistic regression. The research finds that WPV is prevalent among Chinese HCWs, with nurses, young HCWs, night shift workers, and high-income earners being high-risk groups. Particularly noteworthy is that different dimensions of occupational values exhibit complex association patterns with WPV: intrinsic values and altruistic values manifest as factors positively associated with WPV, while extrinsic values, social values, and leisure values serve as factors negatively associated with WPV. These finding challenges conventional perspectives and reveals the double-edged sword effect of occupational values. The differences in results between the two regression methods further unveil the trigger-maintenance mechanism of WPV, providing a scientific basis for targeted interventions. This study not only holds significant theoretical value but also provides empirical support for healthcare institution management, healthcare worker training, and public policy formulation.

## Data Availability

The raw data supporting the conclusions of this article will be made available by the authors, without undue reservation.
