# Supplementary material for: Relationships between professional value and workplace violence among healthcare workers: an empiricle study from China
Source: Front Med (Lausanne). 2026 Apr 21;13:1768667. doi: 10.3389/fmed.2026.1768667 (PMC13140674; doi:10.3389/fmed.2026.1768667)
Supplement: Supplementary file 1 [file Supplementary_file_1.docx]

**Supplementary files**

Contents

[A. Questionaires 2](#_Toc2100343398)

[B. Sample Size Calculation 6](#_Toc135300800)

[C. Sampling Strategy and Recruitment 6](#_Toc1962847074)

[D. Eligibility Criteria 7](#_Toc2074471151)

[E. Participant Flow and Response Rate 8](#_Toc1239625812)

[F. Variable definition and category. 8](#_Toc1652162737)

[G. Associated with Physical Assault 9](#_Toc935565049)

[H. Emotional Abuse Patterns Across Workforce Segments 10](#_Toc166515209)

[I. Threats, Intimidation, and Sexual Harassment Patterns 11](#_Toc449925622)

[J. Supplementary figure 1. Protective and risk factors for physical assault (ordered). 12](#_Toc610007867)

[K. Supplementary figure 2. Protective and risk factors for emotional abuse (ordered). 14](#_Toc315289891)

[L. Supplementary figure 3. Protective and risk factors for threats/intimidation (ordered). 15](#_Toc1235040888)

[M. Supplementary figure 4. Protective and risk factors for verbal sexual harassment (ordered). 17](#_Toc1902756361)

[N. Supplementary figure 5. Protective and risk factors for physical sexual harassment (ordered). 19](#_Toc1447171850)

[O. Supplementary Table 1. Frequency of different types of workplace violence among participants stratified by demographic or professional characteristics 20](#_Toc217497028)

[P. Supplementary Table 2. Factors Associated with Workplace Violence Frequency: Ordered Logistic Regression Results. 40](#_Toc455382402)

# Questionaires

## **Survey on Workplace Violence and Healthcare Professional Values and Influencing Factors among Medical Personnel**

**Instructions:**
Dear Medical Professional,
Greetings. Thank you for taking time out of your busy schedule to participate in this survey. This study aims to understand the current status of work values among medical personnel and their influencing factors. This survey is anonymous, and the results will not affect your work or personal life. Please answer with confidence. We sincerely thank you again for your participation!

### **Part I. General Information**

**1. Gender:**
① Male ② Female

**2. Age:**
① < 30 years ② 30–40 years ③ 40–50 years ④ > 50 years

**3. Marital Status:**
① Single ② Married ③ Divorced ④ Widowed

**4. Education Level:**
① Secondary technical school or below ② Associate degree (Junior college) ③ Bachelor’s degree ④ Master’s degree ⑤ Doctoral degree

**5. Job Position:**
① Physician ② Nurse ③ Pharmacist ④ Medical Technician/Allied Health Professional ⑤ Administrative Staff ⑥ Other

**6. Department:**
① Internal Medicine ② Surgery ③ Medical Technology/Auxiliary ④ Other

**7. Professional Title (Seniority):**
① Senior (e.g., Chief Physician/Professor)
② Deputy Senior (e.g., Associate Chief Physician)
③ Intermediate (e.g., Attending Physician)
④ Junior (e.g., Resident)
⑤ None

**8. Administrative Position:**
① Senior Executive/Manager ② Department Manager/Director ③ Staff/Employee ④ Intern/Trainee/Student

**9. Years of Working Experience in Healthcare:**
① < 1 year ② 1–5 years ③ 6–10 years ④ ≥ 10 years

**10. Night Shift Work:**
① Yes ② No

**11. Type of Employment:**
① Permanent Staff (Bianzhi/Civil Servant equivalent)
② Contract Staff
③ Trainee/Resident (Standardized Training)

**12. Involved in Clinical Teaching:**
① Yes ② No

**13. Type of Hospital:**
① General Hospital ② Specialized Hospital (Please specify: __________)

**14. Level of Hospital:**
① Tertiary A ② Tertiary B ③ Tertiary C
④ Secondary A ⑤ Secondary B ⑥ Secondary C ⑦ Other

**15. Average Monthly Income (RMB):**
① < 2000 ② 2000–4000 ③ 4000–6000 ④ 6000–8000 ⑤ ≥ 8000

**16. Does your current salary align with your workload intensity?**
① Very aligned ② Comparatively aligned ③ Average ④ Comparatively misaligned ⑤ Very misaligned

**17. Self-rated Health Status:**
① Very healthy ② Healthy ③ Average ④ Unhealthy ⑤ Very unhealthy

### **Part II. Work Values Scale**

*Please rate the following items based on your agreement.*

| Item | Strongly Agree | Agree | Neutral | Disagree | Strongly Disagree |
| --- | --- | --- | --- | --- | --- |
| 1. Your work makes you feel like part of a team. | 1 | 2 | 3 | 4 | 5 |
| 2. Your work requires you to manage things in an organized way. |  |  |  |  |  |
| 3. You can utilize your abilities and knowledge in your work. |  |  |  |  |  |
| 4. You frequently receive feedback on your work results. |  |  |  |  |  |
| 5. You are encouraged to be responsible for work outcomes. |  |  |  |  |  |
| 6. You do work that you are personally interested in. |  |  |  |  |  |
| 7. Your work gives you a continuous sense of success. |  |  |  |  |  |
| 8. You can see the results of your hard work. |  |  |  |  |  |
| 9. Your work makes classmates and friends envy you. |  |  |  |  |  |
| 10. Your organization listens to staff opinions when formulating policies. |  |  |  |  |  |
| 11. You have access to various advanced equipment needed for work. |  |  |  |  |  |
| 12. You have a competitive salary linked to performance. |  |  |  |  |  |
| 13. There is a harmonious relationship between your department and others. |  |  |  |  |  |
| 14. You can establish good relationships with colleagues in your work. |  |  |  |  |  |
| 15. You have a reasonable and excellent leader. |  |  |  |  |  |
| 16. Your work is viewed as important by others. |  |  |  |  |  |
| 17. You receive respect from others in the work environment. |  |  |  |  |  |
| 18. No matter how you work, you get promoted and raises like most others. |  |  |  |  |  |
| 19. You have considerable opportunities for promotion. |  |  |  |  |  |
| 20. You frequently participate in professional training (medical, teaching, research). |  |  |  |  |  |
| 21. Your work is recognized by your family. |  |  |  |  |  |
| 22. You don’t have to worry about being scolded or fined financially because leaders are unsatisfied. |  |  |  |  |  |
| 23. Your work brings visible benefits to social welfare. |  |  |  |  |  |
| 24. Your work allows you to often help others. |  |  |  |  |  |
| 25. In your work, serving others gives them satisfaction. |  |  |  |  |  |
| 26. No one frequently disturbs you during your work. |  |  |  |  |  |
| 27. Your working hours (start/end times) are relatively flexible/free. |  |  |  |  |  |
| 28. You are able to enjoy statutory holidays. |  |  |  |  |  |
| 29. Your work provides many flexible benefits. |  |  |  |  |  |
| 30. Once you take this job, you won't be unexpectedly transferred to other units or roles. |  |  |  |  |  |
| 31. You have a comfortable and quiet working environment. |  |  |  |  |  |
| 32. Your current commute transportation is convenient. |  |  |  |  |  |
| 33. Your one-way commute time is moderate. |  |  |  |  |  |
| 34. Your work unit provides certain health guidance. |  |  |  |  |  |

**Definition of Work Value Dimensions:**
The medical personnel work values in this study include five dimensions: Intrinsic, Extrinsic, Social, Altruistic, and Leisure.

1. **Intrinsic Values:** Incentives brought by the work itself, such as influence within the team, role clarity, feedback, responsibility, and interest (Items 1-6).
2. **Extrinsic Values:** Factors that motivate better work, such as salary, material rewards, and status/prestige. Includes achievement, status, promotion, decision-making, resources, and pay (Items 7-12).
3. **Social Values:** Internal incentives gained from the organization, including relationships with colleagues/leaders, recognition, respect, fairness, HR support, training, family support, and security (Items 13-22).
4. **Altruistic Values:** Incentives gained from helping others or social service. Includes contribution to society, directly helping people, and satisfying service (Items 23-25).
5. **Leisure Values:** Factors balancing work and life. Includes basic/flexible benefits, autonomy, freedom, work style, environment, stability, and transportation (Items 26-34).

*(Note: The scale uses a 5-point Likert scoring method: Strongly Agree = 5, Agree = 4, Neutral = 3, Disagree = 2, Strongly Disagree = 1.)*

### **Part III. Workplace Violence Scale**

*During the past 12 months, have you experienced the following workplace violence?*

| Item | Never (0) | 1 time (1) | 2–3 times (2) | ≥ 4 times (3) |
| --- | --- | --- | --- | --- |
| **1. Physical Violence:** (e.g., spitting, biting, hitting, pushing) |  |  |  |  |
| **2. Psychological Violence - Emotional Abuse:** (e.g., swearing, humiliation, making noise/shouting) |  |  |  |  |
| **3. Psychological Violence - Threat/Intimidation:** (e.g., verbal, written, physical gestures, or weapon threats) |  |  |  |  |
| **4. Sexual Harassment - Verbal:** (e.g., repeated unwanted sexual comments) |  |  |  |  |
| **5. Sexual Harassment - Physical:** (e.g., unwanted touching or other forms) |  |  |  |  |

*Scoring: Never = 1 point; 1 time = 2 points; 2-3 times = 3 points; 4 times or more = 4 points.*

### **Part IV. Suggestions for Improvement**

**Comparing the status quo with your work values, which of the following aspects do you hope to see improved? (Multiple choice, select up to 5 items)**

(1) Improve the healthcare system reform and create a good development environment for the healthcare industry.
(2) Strengthen health legislation to protect the legitimate rights and interests of medical personnel.
(3) Improve the hierarchical training system for various health talents.
(4) Establish a compensation system that aligns with the characteristics of the healthcare industry.
(5) Improve the professional title evaluation system in the health industry.
(6) Strengthen the guidance of hospital culture.
(7) Improve hospital management system construction.
(8) Rationalize post settings and improve the post-employment system.
(9) Optimize the internal performance appraisal and distribution system of the hospital.
(10) Improve career development planning and education/training for employees.
(11) Give full play to the role of party and mass organizations to create a good internal environment.
(12) Other.

# Sample Size Calculation

The sample size was calculated based on the formula for estimating prevalence in cross-sectional studies:

$$\text{n = Z²×P×(1-P) / d²}$$

Where:

- Z = 1.96 (corresponding to a 95% confidence level)
- P = 0.50 (estimated prevalence of workplace violence, based on previous studies reporting rates ranging from 40% to 60% among Chinese healthcare workers)
- d = 0.015 (margin of error set at 1.5%)

The calculated minimum sample size was approximately 4,268 participants. Accounting for an anticipated non-response rate of 20-25% and potential incomplete questionnaires, we aimed to recruit approximately 5,350 healthcare workers.

# Sampling Strategy and Recruitment

We employed a multi-stage stratified cluster sampling approach to ensure geographic and institutional diversity:

1. Stage 1: Hospital Selection

Hospitals were stratified by geographic region (Eastern, Central, Western China), hospital level (tertiary, secondary), and hospital type (general, specialized);

Twenty-five hospitals were purposively selected to represent diverse healthcare settings across China;

Selection criteria for hospitals included: (1) willingness to participate, (2) minimum of 200 healthcare workers on staff, and (3) established electronic or paper-based survey distribution systems;

1. Stage 2: Participant Recruitment

Within each participating hospital, we aimed to recruit healthcare workers proportionally from different departments (internal medicine, surgery, emergency, pediatrics, obstetrics and gynecology, and other clinical departments);

Recruitment was facilitated through hospital human resources departments and department heads;

Eligible participants were invited via email, workplace announcements, or direct contact by research coordinators

# Eligibility Criteria

1. **Inclusion criteria:**
2. Currently employed as a healthcare worker (physician, nurse, or allied health professional) at one of the participating hospitals;
3. At least six months of work experience in the current position;
4. Direct patient care responsibilities or regular patient contact;
5. Willing to provide informed consent and complete the questionnaire voluntarily;
6. Able to read and understand Chinese;
7. **Exclusion criteria:**
8. Administrative staff without direct patient care responsibilities;
9. Temporary or contract workers with less than six months of employment;
10. Healthcare workers on extended leave (maternity leave, sick leave, or sabbatical) during the survey period;
11. Incomplete questionnaires with more than 90% of items unanswered across the three assessment instruments;

# **Participant Flow and Response Rate**

Of the 5,350 healthcare workers invited to participate, 5,350 returned questionnaires (initial return rate: 100%). However, quality control procedures identified 1,083 questionnaires (20.24%) that were excluded from analysis due to excessive missing data, defined as completion of less than 10% of items of the 2 assessment instruments (Workplace Violence Scale, Professional Value Questionnaire,). The final analytical sample comprised 4,267 participants from 25 hospitals, yielding an effective response rate of 79.8% (4,267/5,350) and a valid response rate of 67% relative to the initial recruitment target. **S Figure 1** presents a detailed flowchart of participant recruitment and inclusion according to STROBE guidelines.

To evaluate potential non-response bias, we compared the demographic characteristics (age, sex, professional role, hospital level) of respondents with available hospital workforce statistics. No significant differences were observed in age and sex distribution between respondents and the overall workforce at participating hospitals, suggesting minimal non-response bias.

For participants included in the final sample (n = 4,267), missing data on individual questionnaire items ranged from 0.5% to 3.2%. Missing data were handled using multiple imputation. Sensitivity analyses were conducted to assess the impact of missing data on the main findings.

# **Variable definition and category.**

| Variable | Category / Grouping |
| --- | --- |
| **Socio-demographic characteristics** |  |
| Gender | Female / Male |
| Age, yr | <30 / 30-40 / 40-50 / >50 |
| Marital status | Single / Married / Divorced / Widowed |
| Education | Associate / Bachelor / Undergraduate / Master / Doctoral |
| Average monthly income, RMB | <2000 ... >8000 |
| Health condition (rate 0-10) | Poor / Bad / Normal / Healthy / Good |
| **Professional characteristics** |  |
| Type of healthcare professional | Physician / Nurse / Pharmacist / Allied health / Admin / Other |
| Department | General medicine / Surgery / Auxiliary / Other |
| Seniority (Professional Title) | Senior / Deputy senior / Intermediate / Junior |
| Current position | Manager / Employee / Student |
| Working experience, yr | <1 / 1-5 / 6-10 / >10 |
| Type of employee | Labor contract / Contract (Personnel agency) / Temporary |
| Night shift work | Yes / No |
| Teaching duty | Yes / No |
| Alignment: workload & income | Very poor ... Well aligned |
| **Institutional characteristics** |  |
| Type of hospital | General / Specialized |
| Level of hospital | Tertiary A / Tertiary B / Secondary / Other |

# **Associated with Physical Assault**

Gender differences were pronounced in physical assault experiences (χ²=44.407, p<0.001), with male healthcare workers reporting higher rates of multiple incidents (8.9% for 2-3 times; 5.3% for >4 times) compared to females (5.2% and 2.5%, respectively). Educational attainment showed significant associations (χ²=22.218, p=0.035), though the relationship was non-linear. Self-reported health status demonstrated a strong inverse relationship with physical assault (F=155.57, p<0.001), with those rating their health as poor (0-2) experiencing the highest frequency of repeated assaults (10.9% for 2-3 times; 7.3% for >4 times) compared to those reporting good health (4.1% and 1.9%, respectively).

Professional role significantly influenced physical assault exposure (χ²=53.494, p<0.001). Physicians and nurses/midwives experienced comparable rates of repeated physical assault (7.6% and 6.2% for 2-3 times; 3.7% for both groups for >4 times), substantially higher than pharmacists (5.1% and 0.0%), allied health professionals (3.9% and 2.3%), and administrative workers (4.7% and 2.1%). Department-level analysis revealed that general medicine (7.2% for 2-3 times; 3.6% for >4 times) and general surgery departments (6.7% and 2.6%) experienced higher assault rates than medical auxiliary/ancillary services (4.4% and 1.2%).

Career seniority demonstrated a reverse gradient (χ²=62.776, p<0.001), with junior staff experiencing the highest rates of repeated physical assault (8.7% for 2-3 times; 4.2% for >4 times) compared to senior staff (3.8% and 1.6%). Night shift work significantly increased physical assault risk (χ²=29.804, p<0.001), with 11.0% of night shift workers experiencing multiple incidents compared to 6.5% of non-night shift workers. Teaching responsibilities also elevated risk (χ²=45.054, p<0.001), with 11.1% of those with teaching duties experiencing repeated assaults versus 7.7% without such duties.

The alignment between workload and income showed a strong protective gradient (χ²=101.842, p<0.001). Workers reporting very poor alignment experienced the highest rates of repeated physical assault (9.7% for 2-3 times; 8.0% for >4 times), while those reporting well-aligned conditions showed substantially lower rates (4.5% and 1.7%, respectively). Hospital-level characteristics also mattered: specialized hospitals reported higher rates of frequent physical assault (>4 times: 5.7%) compared to general hospitals (2.8%), and secondary-level hospitals showed elevated risks compared to tertiary facilities.

# **Emotional Abuse Patterns Across Workforce Segments**

Emotional abuse, the most prevalent form of WPV, exhibited distinct demographic patterns. Age showed significant associations (F=30.576, p<0.001), with middle-aged workers (30-40 years) experiencing the highest burden of repeated emotional abuse (19.4% once; 19.0% for 2-3 times; 18.8% for >4 times). Income level demonstrated a clear inverse relationship (χ²=83.737, p<0.001), with highest earners (>8,000 RMB monthly) reporting more frequent emotional abuse (21.2% for both 2-3 times and >4 times categories) compared to lowest earners (<2,000 RMB: 17.1% and 12.4%, respectively).

Health status showed the strongest association with emotional abuse (F=252.336, p<0.001). Workers rating their health as poor experienced alarming rates of frequent emotional abuse (40.0% for >4 times), compared to those reporting good health (9.7%). This gradient was consistent across all frequency categories, suggesting potential bidirectional relationships between health status and emotional abuse exposure.

Professional role differentiation was striking (χ²=147.052, p<0.001). Physicians experienced the highest rates of repeated emotional abuse (20.3% for 2-3 times; 19.0% for >4 times), followed closely by nurses/midwives (19.2% and 18.2%), while administrative workers showed substantially lower rates (10.9% and 11.8%). Departmental analysis revealed general medicine (21.2% for 2-3 times; 18.9% for >4 times) and general surgery (20.0% and 19.1%) as high-risk areas, significantly exceeding medical auxiliary/ancillary services (14.5% and 14.0%).

Career stage analysis showed junior staff vulnerability (χ²=111.451, p<0.001), with 22.6% experiencing emotional abuse 2-3 times and 20.6% experiencing it more than four times, compared to senior staff (11.6% for both categories). Working experience followed similar patterns (χ²=59.452, p<0.001), with those having 6-10 years of experience showing peak exposure rates. Night shift work (χ²=78.699, p<0.001) and teaching duties (χ²=108.251, p<0.001) both significantly elevated emotional abuse risk, with teaching staff experiencing particularly high rates of frequent abuse (19.5% for 2-3 times; 20.7% for >4 times).

The workload-income alignment gradient was exceptionally pronounced for emotional abuse (χ²=222.574, p<0.001). Those reporting very poor alignment experienced frequent emotional abuse at rates of 17.7% (2-3 times) and 32.2% (>4 times), compared to those reporting well-aligned conditions (12.5% and 11.3%, respectively).

# **Threats, Intimidation, and Sexual Harassment Patterns**

Threats and intimidation showed significant gender disparities (χ²=44.992, p<0.001), with males reporting higher rates of repeated incidents (10.4% for 2-3 times; 8.7% for >4 times) compared to females (8.2% and 4.7%). Educational attainment demonstrated a positive association (χ²=58.127, p<0.001), with master's degree holders experiencing higher rates (13.2% for 2-3 times; 6.7% for >4 times) compared to associate degree holders (3.9% and 4.7%).

Physicians faced the highest threat exposure (χ²=104.887, p<0.001), with 10.6% experiencing threats 2-3 times and 7.7% more than four times. Career seniority showed a strong inverse gradient (χ²=140.429, p<0.001), with junior staff experiencing 11.6% and 8.5% for these categories, compared to senior staff at 4.0% and 3.5%. The workload-income misalignment effect was substantial (χ²=159.827, p<0.001), with very poor alignment associated with 11.7% and 14.5% rates for repeated threats.

Verbal sexual harassment exhibited significant gender differences (χ²=38.95, p<0.001), with males reporting higher rates across all frequency categories. Health status showed strong associations (F=56.72, p<0.001), with poor health linked to 7.3% experiencing it 2-3 times and 5.5% more than four times. Professional role analysis (χ²=40.315, p<0.001) revealed physicians experiencing the highest rates (4.0% for 2-3 times; 2.5% for >4 times). The workload-income gradient was evident (χ²=73.97, p<0.001), with very poor alignment associated with 5.7% and 5.4% rates for repeated verbal sexual harassment.

Physical sexual harassment, though least prevalent, showed similar patterns. Gender differences were significant (χ²=8.737, p=0.033), with males reporting slightly higher rates. Health status associations were pronounced (F=55.657, p<0.001), and the workload-income alignment gradient persisted (χ²=34.318, p=0.001). Night shift work (χ²=16.691, p=0.001) and teaching duties (χ²=8.711, p=0.033) both elevated physical sexual harassment risk, highlighting occupational exposure factors beyond individual characteristics.

# **Supplementary figure 1. Protective and risk factors for physical assault (ordered).**


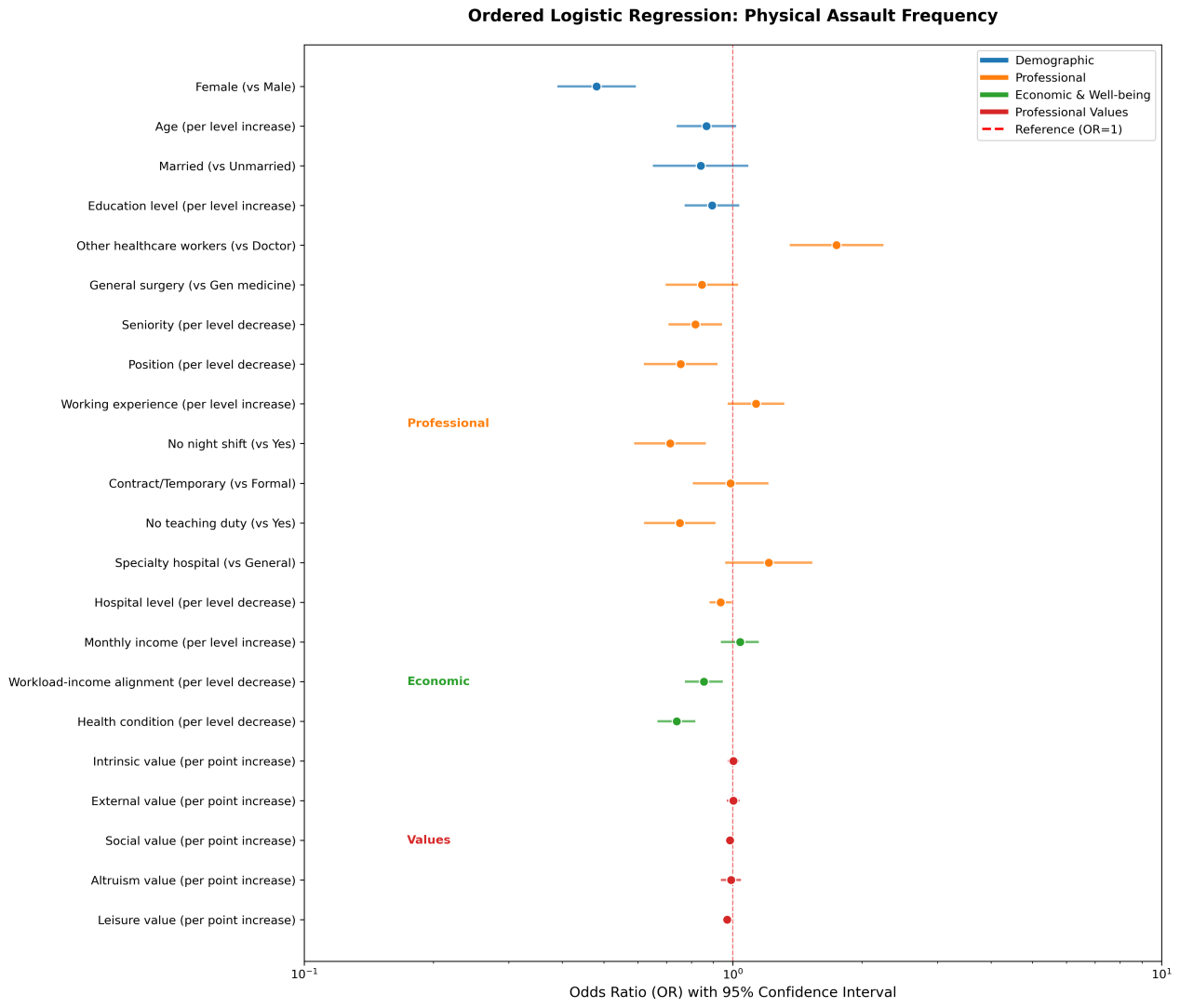


**Supplementary figure 1. Protective and risk factors for physical assault (ordered).**

# **Supplementary figure 2. Protective and risk factors for emotional abuse (ordered).**

**
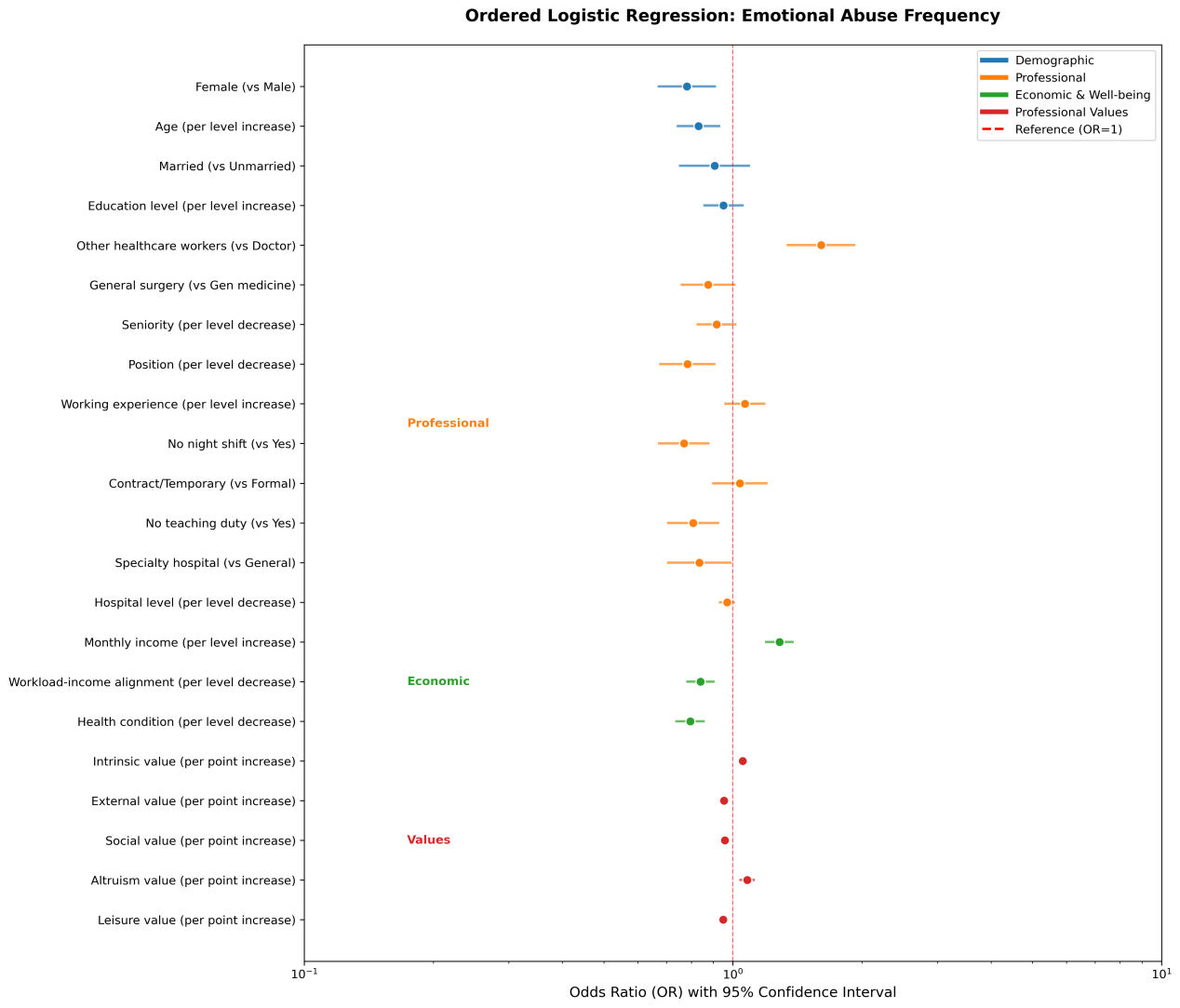
**

**Supplementary figure 2. Protective and risk factors for emotional abuse (ordered).**

# **Supplementary figure 3. Protective and risk factors for threats/intimidation (ordered).**

**
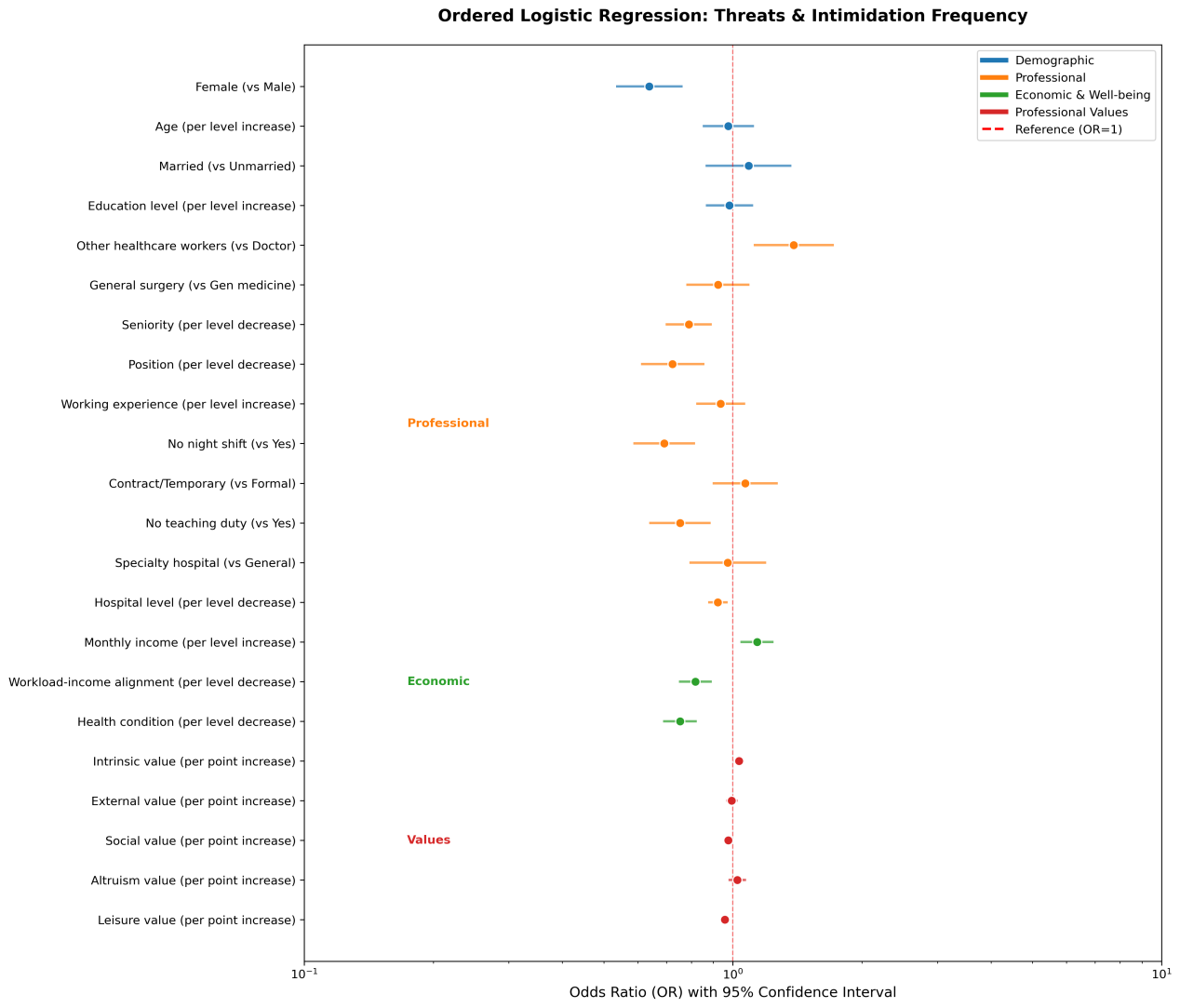
**

**Supplementary figure 3. Protective and risk factors for threats/intimidation (ordered).**

# **Supplementary figure 4. Protective and risk factors for verbal sexual harassment (ordered).**

**
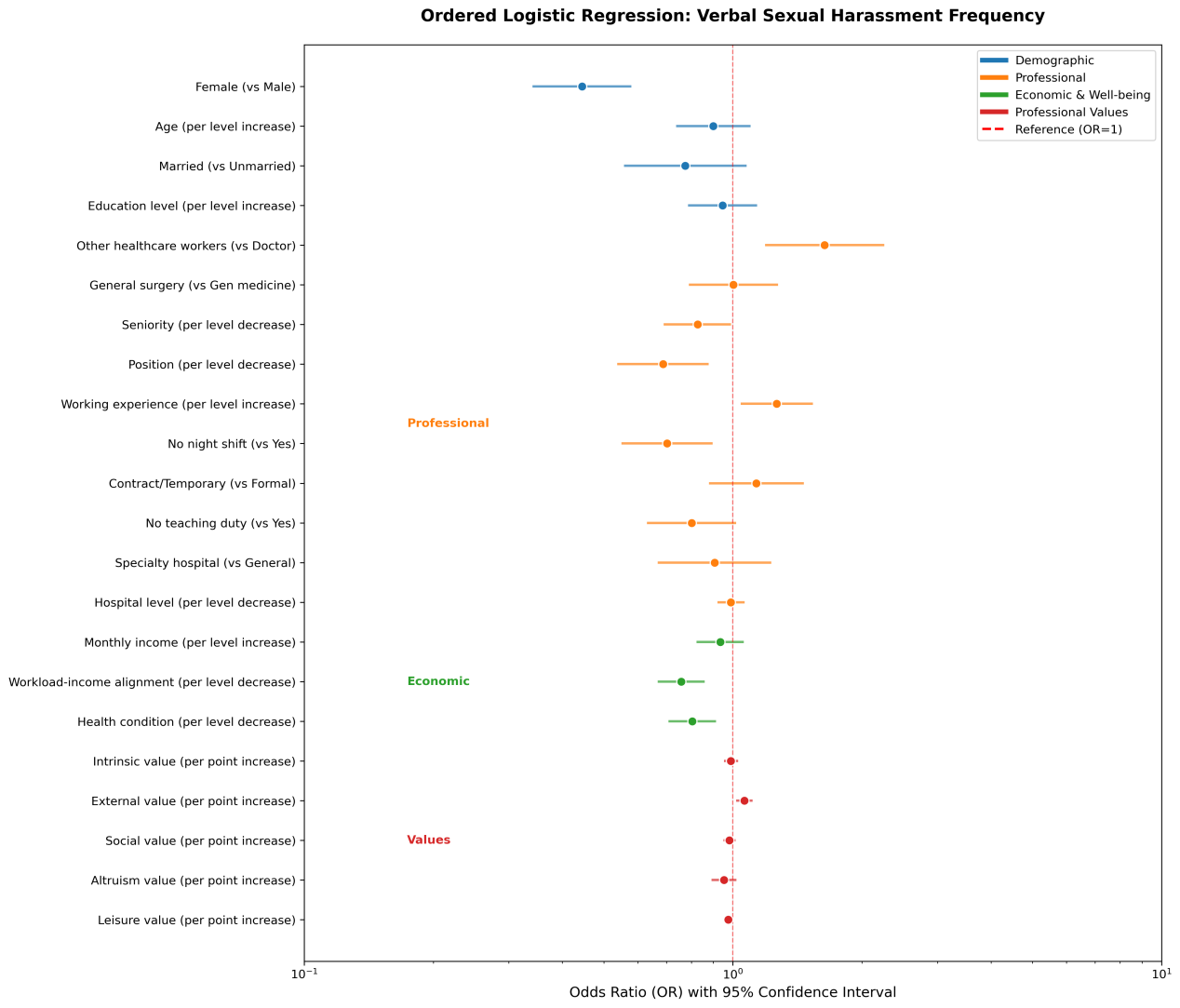
**

**Supplementary figure 4. Protective and risk factors for verbal sexual harassment (ordered).**

# **Supplementary figure 5. Protective and risk factors for physical sexual harassment (ordered).**

**
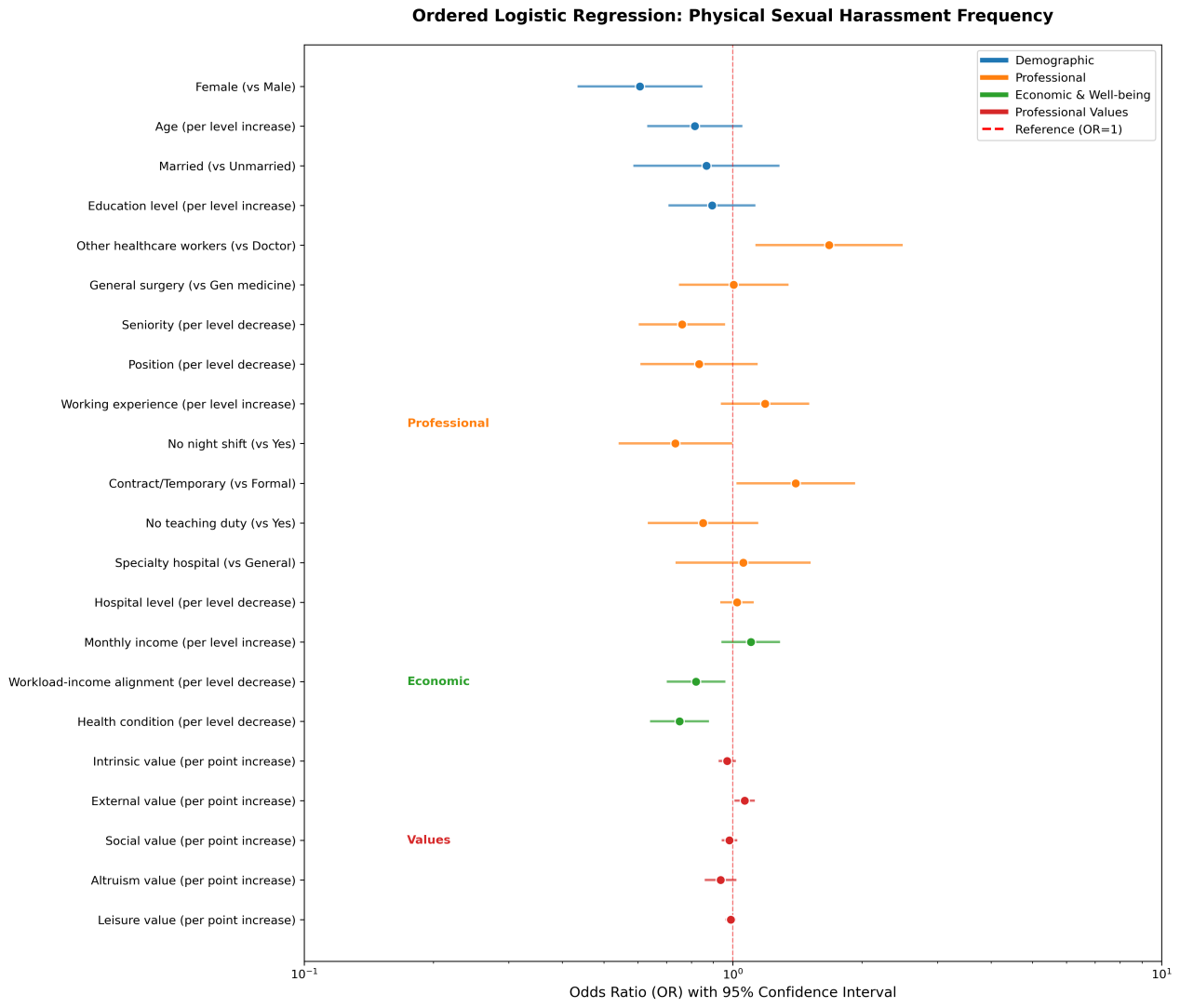
**

# **Supplementary Table 1. Frequency of different types of workplace violence among participants stratified by demographic or professional characteristics**

| **Characteristics** | **Frequency of workplace violence in the preceding 12 months, no. participants (%)** | | | | t/F/χ | **p** | **95% confidence interval** |
| --- | --- | --- | --- | --- | --- | --- | --- |
|  | **Never** | **Once** | **2-3 times** | **>4 times** |  |  |  |
| **I. Physical assault** | | | | | | | |
| **Socio-demographic characteristics** |  |  |  |  |  |  |  |
| **Gender** |  |  |  |  | 44.407 | < 0.001 |  |
| Female | 815 (74.2) | 128 (11.6) | 98 (8.9) | 58 (5.3) |  |  | 0.716-0.767 |
| Male | 2,592 (81.8) | 331 (10.4) | 165 (5.2) | 80 (2.5) |  |  | 0.805-0.832 |
| **Age, yr** |  |  |  |  | 13.319 | 0.149 |  |
| < 30 | 1,028 (82.3) | 108 (8.6) | 68 (5.4) | 45 (3.6) |  |  | 0.802-0.844 |
| 30-40 | 1,501 (79.1) | 217 (11.4) | 122 (6.4) | 58 (3.1) |  |  | 0.773-0.809 |
| 40-50 | 684 (77.9) | 111 (12.6) | 57 (6.5) | 26 (3.0) |  |  | 0.752-0.806 |
| > 50 | 194 (80.2) | 23 (9.5) | 16 (6.6) | 9 (3.7) |  |  | 0.751-0.852 |
| **Marital status** |  |  |  |  | 2.369 | 0.499 |  |
| Single/living with partner | 639 (81.6) | 78 (10.0) | 41 (5.2) | 25 (3.2) |  |  | 0.789-0.843 |
| Married | 2,663 (79.4) | 367 (10.9) | 215 (6.4) | 107 (3.2) |  |  | 0.781-0.808 |
| Separated/divorced | 96 (79.3) | 12 (9.9) | 7 (5.8) | 6 (5.0) |  |  | 0.721-0.866 |
| Widowed | 9 (81.8) | 2 (18.2) | 0 (0.0) | 0 (0.0) |  |  | 0.590-1.046 |
| **Education** |  |  |  |  | 16.708 | 0.010 |  |
| 2-year associate’s degree | 110 (85.9) | 9 (7.0) | 7 (5.5) | 2 (1.6) |  |  | 0.799-0.920 |
| 3-year bachelor’s degree | 977 (82.7) | 106 (9.0) | 63 (5.3) | 35 (3.0) |  |  | 0.806-0.849 |
| Undergraduate diploma | 1,985 (78.2) | 295 (11.6) | 163 (6.4) | 95 (3.7) |  |  | 0.766-0.798 |
| Master's degree | 289 (81.0) | 39 (10.9) | 24 (6.7) | 5 (1.4) |  |  | 0.769-0.850 |
| Doctoral degree | 46 (73.0) | 10 (15.9) | 6 (9.5) | 1 (1.6) |  |  | 0.621-0.840 |
| **Average monthly income, RMB** |  |  |  |  | 12.426 | 0.190 |  |
| < 2000 | 109 (84.5) | 11 (8.5) | 6 (4.7) | 3 (2.3) |  |  | 0.783-0.907 |
| 2000-4000 | 586 (83.5) | 61 (8.7) | 35 (5.0) | 20 (2.8) |  |  | 0.807-0.862 |
| 4000-6000 | 1,000 (80.1) | 131 (10.5) | 71 (5.7) | 46 (3.7) |  |  | 0.779-0.823 |
| 6000-8000 | 845 (79.6) | 112 (10.6) | 71 (6.7) | 33 (3.1) |  |  | 0.772-0.821 |
| > 8000 | 867 (76.9) | 144 (12.8) | 80 (7.1) | 36 (3.2) |  |  | 0.745-0.794 |
| **Health condition (rate 0-10)** |  |  |  |  | 114.463 | < 0.001 |  |
| Poor (0-2) | 29 (52.7) | 16 (29.1) | 6 (10.9) | 4 (7.3) |  |  | 0.395-0.659 |
| Bad (3-4) | 309 (66.7) | 65 (14.0) | 56 (12.1) | 33 (7.1) |  |  | 0.624-0.710 |
| Normal (5-6) | 1,208 (76.8) | 207 (13.2) | 116 (7.4) | 42 (2.7) |  |  | 0.747-0.789 |
| Healthy (7-8) | 1,620 (85.0) | 159 (8.3) | 74 (3.9) | 54 (2.8) |  |  | 0.833-0.866 |
| Good (9-10) | 241 (89.6) | 12 (4.5) | 11 (4.1) | 5 (1.9) |  |  | 0.859-0.932 |
|  |  |  |  |  |  |  |  |
| **Type of healthcare professional** |  |  |  |  | 52.778 | < 0.001 |  |
| Physician | 1,055 (76.3) | 171 (12.4) | 105 (7.6) | 51 (3.7) |  |  | 0.741-0.786 |
| Nurse/midwife | 1,490 (78.5) | 222 (11.7) | 117 (6.2) | 70 (3.7) |  |  | 0.766-0.803 |
| Pharmacist | 104 (88.9) | 7 (6.0) | 6 (5.1) | 0 (0.0) |  |  | 0.832-0.946 |
| Allied health professional (therapist/radiographer/assistant) | 332 (86.2) | 29 (7.5) | 15 (3.9) | 9 (2.3) |  |  | 0.828-0.897 |
| Administrative or clerical worker | 294 (87.0) | 21 (6.2) | 16 (4.7) | 7 (2.1) |  |  | 0.834-0.906 |
| Other | 132 (90.4) | 9 (6.2) | 4 (2.7) | 1 (0.7) |  |  | 0.856-0.952 |
| **Department** |  |  |  |  | 54.617 | < 0.001 |  |
| General medicine | 1,035 (76.6) | 171 (12.6) | 97 (7.2) | 49 (3.6) |  |  | 0.743-0.788 |
| General surgery | 892 (77.8) | 148 (12.9) | 77 (6.7) | 30 (2.6) |  |  | 0.754-0.802 |
| Medical auxiliary/ancillary | 578 (87.2) | 48 (7.2) | 29 (4.4) | 8 (1.2) |  |  | 0.846-0.897 |
| Other | 902 (81.6) | 92 (8.3) | 60 (5.4) | 51 (4.6) |  |  | 0.793-0.839 |
| **Seniority** |  |  |  |  | 49.878 | < 0.001 |  |
| Senior | 79 (73.1) | 15 (13.9) | 8 (7.4) | 6 (5.6) |  |  | 0.648-0.815 |
| Deputy senior | 457 (70.6) | 107 (16.5) | 56 (8.7) | 27 (4.2) |  |  | 0.671-0.741 |
| Intermediate | 1,124 (80.8) | 147 (10.6) | 85 (6.1) | 35 (2.5) |  |  | 0.787-0.829 |
| Junior | 1,418 (81.1) | 167 (9.5) | 100 (5.7) | 64 (3.7) |  |  | 0.792-0.829 |
| Not reported | 329 (88.4) | 23 (6.2) | 14 (3.8) | 6 (1.6) |  |  | 0.852-0.917 |
| **Current position** |  |  |  |  | 10.115 | 0.018 |  |
| Hospital manager | 67 (80.7) | 9 (10.8) | 4 (4.8) | 3 (3.6) |  |  | 0.722-0.892 |
| Department manager | 666 (75.9) | 115 (13.1) | 65 (7.4) | 32 (3.6) |  |  | 0.730-0.787 |
| Employee | 2,610 (80.7) | 331 (10.2) | 193 (6.0) | 102 (3.2) |  |  | 0.793-0.820 |
| Intern/student/trainee | 64 (91.4) | 4 (5.7) | 1 (1.4) | 1 (1.4) |  |  | 0.849-0.980 |
| **Working experience, yr** |  |  |  |  | 31.911 | < 0.001 |  |
| < 1 | 163 (89.1) | 8 (4.4) | 9 (4.9) | 3 (1.6) |  |  | 0.846-0.936 |
| 1-5 | 631 (85.0) | 58 (7.8) | 30 (4.0) | 23 (3.1) |  |  | 0.825-0.876 |
| 6-10 | 923 (78.8) | 131 (11.2) | 75 (6.4) | 42 (3.6) |  |  | 0.765-0.812 |
| > 10 | 1,690 (77.8) | 262 (12.1) | 149 (6.9) | 70 (3.2) |  |  | 0.761-0.796 |
| **Type of employee** |  |  |  |  | 13.785 | 0.003 |  |
| Employee under labor contract | 1,548 (77.7) | 252 (12.7) | 129 (6.5) | 62 (3.1) |  |  | 0.759-0.796 |
| Employee under contract | 1,777 (81.4) | 201 (9.2) | 131 (6.0) | 74 (3.4) |  |  | 0.798-0.830 |
| Temporary worker | 82 (88.2) | 6 (6.5) | 3 (3.2) | 2 (2.2) |  |  | 0.816-0.947 |
| **Night shift work** |  |  |  |  | 29.804 | < 0.001 |  |
| Yes | 2,098 (77.5) | 310 (11.5) | 196 (7.2) | 103 (3.8) |  |  | 0.759-0.791 |
| No | 1,309 (83.9) | 149 (9.6) | 67 (4.3) | 35 (2.2) |  |  | 0.821-0.857 |
| **Teaching duty** |  |  |  |  | 45.054 | < 0.001 |  |
| Yes | 1,612 (75.8) | 280 (13.2) | 158 (7.4) | 78 (3.7) |  |  | 0.739-0.776 |
| No | 1,795 (83.9) | 179 (8.4) | 105 (4.9) | 60 (2.8) |  |  | 0.824-0.855 |
| **Alignment between workload and income** |  |  |  |  | 100.943 | < 0.001 |  |
| Very poor | 232 (66.1) | 57 (16.2) | 34 (9.7) | 28 (8.0) |  |  | 0.611-0.710 |
| Poor | 920 (75.2) | 151 (12.3) | 100 (8.2) | 53 (4.3) |  |  | 0.727-0.776 |
| Normal | 1,338 (82.7) | 161 (10.0) | 81 (5.0) | 37 (2.3) |  |  | 0.809-0.846 |
| Aligned | 852 (85.6) | 81 (8.1) | 45 (4.5) | 17 (1.7) |  |  | 0.834-0.878 |
| Well | 65 (81.2) | 9 (11.2) | 3 (3.8) | 3 (3.8) |  |  | 0.727-0.898 |
|  |  |  |  |  |  |  |  |
| **Type of hospital** |  |  |  |  | 26.109 | < 0.001 |  |
| General | 2,865 (79.6) | 413 (11.5) | 219 (6.1) | 100 (2.8) |  |  | 0.783-0.810 |
| Specialized | 542 (80.9) | 46 (6.9) | 44 (6.6) | 38 (5.7) |  |  | 0.779-0.839 |
| **Level of hospital** |  |  |  |  | 30.742 | < 0.001 |  |
| Tertiary A | 1,508 (77.6) | 247 (12.7) | 133 (6.8) | 55 (2.8) |  |  | 0.758-0.795 |
| Tertiary B | 806 (80.4) | 106 (10.6) | 61 (6.1) | 29 (2.9) |  |  | 0.780-0.829 |
| Tertiary C | 6 (85.7) | 1 (14.3) | 0 (0.0) | 0 (0.0) |  |  | 0.598-1.116 |
| Secondary A | 795 (82.1) | 75 (7.7) | 53 (5.5) | 45 (4.6) |  |  | 0.797-0.845 |
| Secondary B | 94 (86.2) | 7 (6.4) | 6 (5.5) | 2 (1.8) |  |  | 0.798-0.927 |
| Secondary C | 5 (100.0) | 0 (0.0) | 0 (0.0) | 0 (0.0) |  |  | 1.000-1.000 |
| Health center/private hospital | 193 (82.8) | 23 (9.9) | 10 (4.3) | 7 (3.0) |  |  | 0.780-0.877 |
| **II. Emotional abuse** | | | | | | | |
| **Socio-demographic characteristics** |  |  |  |  |  |  |  |
| **Gender** |  |  |  |  |  |  |  |
| Female | 478 (43.5) | 204 (18.6) | 204 (18.6) | 213 (19.4) |  |  | 0.406-0.464 |
| Male | 1,495 (47.2) | 595 (18.8) | 556 (17.6) | 522 (16.5) |  |  | 0.455-0.489 |
| **Age, yr** |  |  |  |  | 30.576 | < 0.001 |  |
| < 30 | 638 (51.1) | 200 (16.0) | 208 (16.7) | 203 (16.3) |  |  | 0.483-0.539 |
| 30-40 | 813 (42.8) | 368 (19.4) | 360 (19.0) | 357 (18.8) |  |  | 0.406-0.451 |
| 40-50 | 400 (45.6) | 184 (21.0) | 160 (18.2) | 134 (15.3) |  |  | 0.423-0.489 |
| > 50 | 122 (50.4) | 47 (19.4) | 32 (13.2) | 41 (16.9) |  |  | 0.441-0.567 |
| **Marital status** |  |  |  |  | 18.234 | 0.006 |  |
| Single/living with partner | 401 (51.2) | 113 (14.4) | 129 (16.5) | 140 (17.9) |  |  | 0.477-0.547 |
| Married | 1,517 (45.3) | 663 (19.8) | 604 (18.0) | 568 (16.9) |  |  | 0.436-0.469 |
| Separated/divorced | 52 (43.0) | 19 (15.7) | 24 (19.8) | 26 (21.5) |  |  | 0.342-0.518 |
| Widowed | 3 (27.3) | 4 (36.4) | 3 (27.3) | 1 (9.1) |  |  | 0.010-0.536 |
| **Education** |  |  |  |  | 60.424 | < 0.001 |  |
| 2-year associate’s degree | 86 (67.2) | 14 (10.9) | 13 (10.2) | 15 (11.7) |  |  | 0.591-0.753 |
| 3-year bachelor’s degree | 610 (51.7) | 190 (16.1) | 195 (16.5) | 186 (15.7) |  |  | 0.488-0.545 |
| Undergraduate diploma | 1,116 (44.0) | 491 (19.3) | 474 (18.7) | 457 (18.0) |  |  | 0.420-0.459 |
| Master’s degree | 136 (38.1) | 90 (25.2) | 62 (17.4) | 69 (19.3) |  |  | 0.331-0.431 |
| Doctoral degree | 25 (39.7) | 14 (22.2) | 16 (25.4) | 8 (12.7) |  |  | 0.276-0.518 |
| **Average monthly income, RMB** |  |  |  |  | 83.737 | < 0.001 |  |
| < 2000 | 75 (58.1) | 16 (12.4) | 22 (17.1) | 16 (12.4) |  |  | 0.496-0.667 |
| 2000-4000 | 396 (56.4) | 123 (17.5) | 89 (12.7) | 94 (13.4) |  |  | 0.527-0.601 |
| 4000-6000 | 620 (49.7) | 210 (16.8) | 201 (16.1) | 217 (17.4) |  |  | 0.469-0.525 |
| 6000-8000 | 448 (42.2) | 211 (19.9) | 209 (19.7) | 193 (18.2) |  |  | 0.393-0.452 |
| > 8000 | 434 (38.5) | 239 (21.2) | 239 (21.2) | 215 (19.1) |  |  | 0.357-0.414 |
| **Health condition (rate 0-10)** |  |  |  |  | 252.336 | < 0.001 |  |
| Poor (0-2) | 15 (27.3) | 9 (16.4) | 9 (16.4) | 22 (40.0) |  |  | 0.155-0.390 |
| Bad (3-4) | 130 (28.1) | 81 (17.5) | 109 (23.5) | 143 (30.9) |  |  | 0.240-0.322 |
| Normal (5-6) | 616 (39.2) | 329 (20.9) | 314 (20.0) | 314 (20.0) |  |  | 0.367-0.416 |
| Healthy (7-8) | 1,032 (54.1) | 348 (18.2) | 297 (15.6) | 230 (12.1) |  |  | 0.519-0.564 |
| Good (9-10) | 180 (66.9) | 32 (11.9) | 31 (11.5) | 26 (9.7) |  |  | 0.613-0.725 |
|  |  |  |  |  |  |  |  |
| **Type of healthcare professional** |  |  |  |  | 147.052 | < 0.001 |  |
| Physician | 523 (37.8) | 316 (22.9) | 281 (20.3) | 262 (19.0) |  |  | 0.353-0.404 |
| Nurse/midwife | 851 (44.8) | 338 (17.8) | 364 (19.2) | 346 (18.2) |  |  | 0.426-0.470 |
| Pharmacist | 65 (55.6) | 23 (19.7) | 14 (12.0) | 15 (12.8) |  |  | 0.466-0.646 |
| Allied health professional (therapist/radiographer/assistant) | 215 (55.8) | 71 (18.4) | 45 (11.7) | 54 (14.0) |  |  | 0.509-0.608 |
| Administrative or clerical worker | 228 (67.5) | 33 (9.8) | 37 (10.9) | 40 (11.8) |  |  | 0.625-0.725 |
| Other | 91 (62.3) | 18 (12.3) | 19 (13.0) | 18 (12.3) |  |  | 0.545-0.702 |
| **Department** |  |  |  |  | 128.492 | < 0.001 |  |
| General medicine | 509 (37.6) | 302 (22.3) | 286 (21.2) | 255 (18.9) |  |  | 0.351-0.402 |
| General surgery | 476 (41.5) | 223 (19.4) | 229 (20.0) | 219 (19.1) |  |  | 0.386-0.444 |
| Medical auxiliary/ancillary | 350 (52.8) | 124 (18.7) | 96 (14.5) | 93 (14.0) |  |  | 0.490-0.566 |
| Other | 638 (57.7) | 150 (13.6) | 149 (13.5) | 168 (15.2) |  |  | 0.548-0.607 |
| **Seniority** |  |  |  |  | 111.451 | < 0.001 |  |
| Senior | 43 (39.8) | 38 (35.2) | 14 (13.0) | 13 (12.0) |  |  | 0.306-0.490 |
| Deputy senior | 233 (36.0) | 135 (20.9) | 146 (22.6) | 133 (20.6) |  |  | 0.323-0.397 |
| Intermediate | 599 (43.1) | 286 (20.6) | 255 (18.3) | 251 (18.0) |  |  | 0.405-0.457 |
| Junior | 858 (49.1) | 294 (16.8) | 302 (17.3) | 295 (16.9) |  |  | 0.467-0.514 |
| Not reported | 240 (64.5) | 46 (12.4) | 43 (11.6) | 43 (11.6) |  |  | 0.597-0.694 |
| **Current position** |  |  |  |  | 16.493 | 0.057 |  |
| Hospital manager | 42 (50.6) | 13 (15.7) | 19 (22.9) | 9 (10.8) |  |  | 0.398-0.614 |
| Department manager | 374 (42.6) | 165 (18.8) | 176 (20.0) | 163 (18.6) |  |  | 0.393-0.459 |
| Employee | 1,518 (46.9) | 605 (18.7) | 558 (17.2) | 555 (17.2) |  |  | 0.452-0.486 |
| Intern/student/trainee | 39 (55.7) | 16 (22.9) | 7 (10.0) | 8 (11.4) |  |  | 0.441-0.674 |
| **Working experience, yr** |  |  |  |  | 59.452 | < 0.001 |  |
| < 1 | 119 (65.0) | 27 (14.8) | 26 (14.2) | 11 (6.0) |  |  | 0.581-0.719 |
| 1-5 | 397 (53.5) | 117 (15.8) | 112 (15.1) | 116 (15.6) |  |  | 0.499-0.571 |
| 6-10 | 507 (43.3) | 215 (18.4) | 219 (18.7) | 230 (19.6) |  |  | 0.405-0.461 |
| > 10 | 950 (43.8) | 440 (20.3) | 403 (18.6) | 378 (17.4) |  |  | 0.417-0.458 |
| **Type of employee** |  |  |  |  | 18.353 | 0.005 |  |
| Employee under labor contract | 865 (43.4) | 403 (20.2) | 370 (18.6) | 353 (17.7) |  |  | 0.413-0.456 |
| Employee under contract | 1,057 (48.4) | 376 (17.2) | 376 (17.2) | 374 (17.1) |  |  | 0.463-0.505 |
| Temporary worker | 51 (54.8) | 20 (21.5) | 14 (15.1) | 8 (8.6) |  |  | 0.447-0.650 |
| **Night shift work** |  |  |  |  | 78.699 | < 0.001 |  |
| Yes | 1,116 (41.2) | 533 (19.7) | 534 (19.7) | 524 (19.4) |  |  | 0.394-0.431 |
| No | 857 (54.9) | 266 (17.1) | 226 (14.5) | 211 (13.5) |  |  | 0.525-0.574 |
| **Teaching duty** |  |  |  |  | 108.251 | < 0.001 |  |
| Yes | 818 (38.4) | 454 (21.3) | 415 (19.5) | 441 (20.7) |  |  | 0.364-0.405 |
| No | 1,155 (54.0) | 345 (16.1) | 345 (16.1) | 294 (13.7) |  |  | 0.519-0.561 |
| **Alignment between workload and income** |  |  |  |  | 222.574 | < 0.001 |  |
| Very poor | 110 (31.3) | 66 (18.8) | 62 (17.7) | 113 (32.2) |  |  | 0.265-0.362 |
| Poor | 437 (35.7) | 245 (20.0) | 263 (21.5) | 279 (22.8) |  |  | 0.330-0.384 |
| Normal | 787 (48.7) | 312 (19.3) | 274 (16.9) | 244 (15.1) |  |  | 0.462-0.511 |
| Aligned | 587 (59.0) | 167 (16.8) | 151 (15.2) | 90 (9.0) |  |  | 0.559-0.621 |
| Well | 52 (65.0) | 9 (11.2) | 10 (12.5) | 9 (11.2) |  |  | 0.545-0.755 |
|  |  |  |  |  |  |  |  |
| **Type of hospital** |  |  |  |  | 11.615 | 0.009 |  |
| General | 1,624 (45.1) | 687 (19.1) | 648 (18.0) | 638 (17.7) |  |  | 0.435-0.468 |
| Specialized | 349 (52.1) | 112 (16.7) | 112 (16.7) | 97 (14.5) |  |  | 0.483-0.559 |
| **Level of hospital** |  |  |  |  | 48.831 | < 0.001 |  |
| Tertiary A | 801 (41.2) | 387 (19.9) | 375 (19.3) | 380 (19.6) |  |  | 0.390-0.434 |
| Tertiary B | 473 (47.2) | 190 (19.0) | 172 (17.2) | 167 (16.7) |  |  | 0.441-0.503 |
| Tertiary C | 6 (85.7) | 0 (0.0) | 1 (14.3) | 0 (0.0) |  |  | 0.598-1.116 |
| Secondary A | 511 (52.8) | 163 (16.8) | 158 (16.3) | 136 (14.0) |  |  | 0.496-0.559 |
| Secondary B | 62 (56.9) | 15 (13.8) | 20 (18.3) | 12 (11.0) |  |  | 0.476-0.662 |
| Secondary C | 3 (60.0) | 2 (40.0) | 0 (0.0) | 0 (0.0) |  |  | 0.171-1.029 |
| Health center/private hospital | 117 (50.2) | 42 (18.0) | 34 (14.6) | 40 (17.2) |  |  | 0.438-0.566 |
| **III. Threats and intimidation** | | | | | | | |
| **Socio-demographic characteristics** |  |  |  |  |  |  |  |
| **Gender** |  |  |  |  | 44.992 | < 0.001 |  |
| Female | 686 (62.4) | 203 (18.5) | 114 (10.4) | 96 (8.7) |  |  | 0.596-0.653 |
| Male | 2,284 (72.1) | 476 (15.0) | 260 (8.2) | 148 (4.7) |  |  | 0.705-0.737 |
| **Age, yr** |  |  |  |  | 44.767 | < 0.001 |  |
| < 30 | 947 (75.8) | 156 (12.5) | 92 (7.4) | 54 (4.3) |  |  | 0.734-0.782 |
| 30-40 | 1,288 (67.9) | 303 (16.0) | 191 (10.1) | 116 (6.1) |  |  | 0.658-0.700 |
| 40-50 | 585 (66.6) | 168 (19.1) | 70 (8.0) | 55 (6.3) |  |  | 0.635-0.697 |
| > 50 | 150 (62.0) | 52 (21.5) | 21 (8.7) | 19 (7.9) |  |  | 0.559-0.681 |
| **Marital status** |  |  |  |  | 26.186 | < 0.001 |  |
| Single/living with partner | 601 (76.8) | 87 (11.1) | 53 (6.8) | 42 (5.4) |  |  | 0.738-0.797 |
| Married | 2,285 (68.2) | 566 (16.9) | 307 (9.2) | 194 (5.8) |  |  | 0.666-0.697 |
| Separated/divorced | 78 (64.5) | 23 (19.0) | 13 (10.7) | 7 (5.8) |  |  | 0.559-0.730 |
| Widowed | 6 (54.5) | 3 (27.3) | 1 (9.1) | 1 (9.1) |  |  | 0.251-0.840 |
| **Education** |  |  |  |  | 56.883 | < 0.001 |  |
| 2-year associate’s degree | 104 (81.2) | 13 (10.2) | 5 (3.9) | 6 (4.7) |  |  | 0.745-0.880 |
| 3-year bachelor’s degree | 895 (75.8) | 157 (13.3) | 78 (6.6) | 51 (4.3) |  |  | 0.733-0.782 |
| Undergraduate diploma | 1,722 (67.8) | 419 (16.5) | 238 (9.4) | 159 (6.3) |  |  | 0.660-0.697 |
| Master’s degree | 209 (58.5) | 77 (21.6) | 47 (13.2) | 24 (6.7) |  |  | 0.534-0.637 |
| Doctoral degree | 40 (63.5) | 13 (20.6) | 6 (9.5) | 4 (6.3) |  |  | 0.516-0.754 |
| **Average monthly income, RMB** |  |  |  |  | 91.307 | < 0.001 |  |
| < 2000 | 100 (77.5) | 18 (14.0) | 5 (3.9) | 6 (4.7) |  |  | 0.703-0.847 |
| 2000-4000 | 550 (78.3) | 91 (13.0) | 36 (5.1) | 25 (3.6) |  |  | 0.753-0.814 |
| 4000-6000 | 916 (73.4) | 157 (12.6) | 108 (8.7) | 67 (5.4) |  |  | 0.709-0.758 |
| 6000-8000 | 722 (68.0) | 172 (16.2) | 99 (9.3) | 68 (6.4) |  |  | 0.652-0.709 |
| > 8000 | 682 (60.5) | 241 (21.4) | 126 (11.2) | 78 (6.9) |  |  | 0.577-0.634 |
| **Health condition (rate 0-10)** |  |  |  |  | 162.983 | < 0.001 |  |
| Poor (0-2) | 23 (41.8) | 12 (21.8) | 10 (18.2) | 10 (18.2) |  |  | 0.288-0.549 |
| Bad (3-4) | 243 (52.5) | 100 (21.6) | 58 (12.5) | 62 (13.4) |  |  | 0.479-0.570 |
| Normal (5-6) | 1,018 (64.7) | 302 (19.2) | 170 (10.8) | 83 (5.3) |  |  | 0.624-0.671 |
| Healthy (7-8) | 1,466 (76.9) | 239 (12.5) | 124 (6.5) | 78 (4.1) |  |  | 0.750-0.788 |
| Good (9-10) | 220 (81.8) | 26 (9.7) | 12 (4.5) | 11 (4.1) |  |  | 0.772-0.864 |
|  |  |  |  |  |  |  |  |
| **Type of healthcare professional** |  |  |  |  | 104.887 | < 0.001 |  |
| Physician | 842 (60.9) | 287 (20.8) | 147 (10.6) | 106 (7.7) |  |  | 0.584-0.635 |
| Nurse/midwife | 1,342 (70.7) | 295 (15.5) | 168 (8.8) | 94 (4.9) |  |  | 0.686-0.727 |
| Pharmacist | 91 (77.8) | 12 (10.3) | 10 (8.5) | 4 (3.4) |  |  | 0.702-0.853 |
| Allied health professional (therapist/radiographer/assistant) | 305 (79.2) | 42 (10.9) | 23 (6.0) | 15 (3.9) |  |  | 0.752-0.833 |
| Administrative or clerical worker | 270 (79.9) | 32 (9.5) | 17 (5.0) | 19 (5.6) |  |  | 0.756-0.842 |
| Other | 120 (82.2) | 11 (7.5) | 9 (6.2) | 6 (4.1) |  |  | 0.760-0.884 |
| **Department** |  |  |  |  | 55.847 | < 0.001 |  |
| General medicine | 886 (65.5) | 244 (18.0) | 136 (10.1) | 86 (6.4) |  |  | 0.630-0.681 |
| General surgery | 749 (65.3) | 220 (19.2) | 110 (9.6) | 68 (5.9) |  |  | 0.625-0.681 |
| Medical auxiliary/ancillary | 504 (76.0) | 78 (11.8) | 54 (8.1) | 27 (4.1) |  |  | 0.728-0.793 |
| Other | 831 (75.2) | 137 (12.4) | 74 (6.7) | 63 (5.7) |  |  | 0.727-0.777 |
| **Seniority** |  |  |  |  | 140.429 | < 0.001 |  |
| Senior | 59 (54.6) | 30 (27.8) | 10 (9.3) | 9 (8.3) |  |  | 0.452-0.640 |
| Deputy senior | 358 (55.3) | 159 (24.6) | 75 (11.6) | 55 (8.5) |  |  | 0.515-0.592 |
| Intermediate | 941 (67.6) | 224 (16.1) | 137 (9.8) | 89 (6.4) |  |  | 0.652-0.701 |
| Junior | 1,297 (74.2) | 237 (13.6) | 137 (7.8) | 78 (4.5) |  |  | 0.721-0.762 |
| Not reported | 315 (84.7) | 29 (7.8) | 15 (4.0) | 13 (3.5) |  |  | 0.810-0.883 |
| **Current position** |  |  |  |  | 16.004 | 0.001 |  |
| Hospital manager | 48 (57.8) | 20 (24.1) | 13 (15.7) | 2 (2.4) |  |  | 0.472-0.685 |
| Department manager | 567 (64.6) | 152 (17.3) | 90 (10.3) | 69 (7.9) |  |  | 0.614-0.677 |
| Employee | 2,296 (71.0) | 501 (15.5) | 270 (8.3) | 169 (5.2) |  |  | 0.694-0.725 |
| Intern/student/trainee | 59 (84.3) | 6 (8.6) | 1 (1.4) | 4 (5.7) |  |  | 0.758-0.928 |
| **Working experience, yr** |  |  |  |  | 64.697 | < 0.001 |  |
| < 1 | 159 (86.9) | 17 (9.3) | 4 (2.2) | 3 (1.6) |  |  | 0.820-0.918 |
| 1-5 | 569 (76.7) | 86 (11.6) | 54 (7.3) | 33 (4.4) |  |  | 0.736-0.797 |
| 6-10 | 800 (68.3) | 182 (15.5) | 126 (10.8) | 63 (5.4) |  |  | 0.657-0.710 |
| > 10 | 1,442 (66.4) | 394 (18.1) | 190 (8.8) | 145 (6.7) |  |  | 0.644-0.684 |
| **Type of employee** |  |  |  |  | 35.163 | < 0.001 |  |
| Employee under labor contract | 1,303 (65.4) | 364 (18.3) | 190 (9.5) | 134 (6.7) |  |  | 0.634-0.675 |
| Employee under contract | 1,592 (72.9) | 306 (14.0) | 180 (8.2) | 105 (4.8) |  |  | 0.711-0.748 |
| Temporary worker | 75 (80.6) | 9 (9.7) | 4 (4.3) | 5 (5.4) |  |  | 0.726-0.887 |
| **Night shift work** |  |  |  |  | 45.435 | < 0.001 |  |
| Yes | 1,790 (66.1) | 472 (17.4) | 278 (10.3) | 167 (6.2) |  |  | 0.643-0.679 |
| No | 1,180 (75.6) | 207 (13.3) | 96 (6.2) | 77 (4.9) |  |  | 0.735-0.778 |
| **Teaching duty** |  |  |  |  | 100.409 | < 0.001 |  |
| Yes | 1,331 (62.5) | 411 (19.3) | 234 (11.0) | 152 (7.1) |  |  | 0.605-0.646 |
| No | 1,639 (76.6) | 268 (12.5) | 140 (6.5) | 92 (4.3) |  |  | 0.748-0.784 |
| **Alignment between workload and income** |  |  |  |  | 155.854 | < 0.001 |  |
| Very poor | 194 (55.3) | 65 (18.5) | 41 (11.7) | 51 (14.5) |  |  | 0.501-0.605 |
| Poor | 748 (61.1) | 241 (19.7) | 152 (12.4) | 83 (6.8) |  |  | 0.584-0.638 |
| Normal | 1,192 (73.7) | 233 (14.4) | 120 (7.4) | 72 (4.5) |  |  | 0.716-0.759 |
| Aligned | 774 (77.8) | 130 (13.1) | 58 (5.8) | 33 (3.3) |  |  | 0.752-0.804 |
| Well | 62 (77.5) | 10 (12.5) | 3 (3.8) | 5 (6.2) |  |  | 0.683-0.867 |
|  |  |  |  |  |  |  |  |
| **Type of hospital** |  |  |  |  | 6.723 | 0.081 |  |
| General | 2,476 (68.8) | 590 (16.4) | 320 (8.9) | 211 (5.9) |  |  | 0.673-0.703 |
| Specialized | 494 (73.7) | 89 (13.3) | 54 (8.1) | 33 (4.9) |  |  | 0.704-0.771 |
| **Level of hospital** |  |  |  |  | 62.171 | < 0.001 |  |
| Tertiary A | 1,251 (64.4) | 352 (18.1) | 207 (10.7) | 133 (6.8) |  |  | 0.623-0.665 |
| Tertiary B | 710 (70.9) | 153 (15.3) | 88 (8.8) | 51 (5.1) |  |  | 0.680-0.737 |
| Tertiary C | 6 (85.7) | 1 (14.3) | 0 (0.0) | 0 (0.0) |  |  | 0.598-1.116 |
| Secondary A | 735 (75.9) | 128 (13.2) | 60 (6.2) | 45 (4.6) |  |  | 0.732-0.786 |
| Secondary B | 92 (84.4) | 12 (11.0) | 4 (3.7) | 1 (0.9) |  |  | 0.776-0.912 |
| Secondary C | 2 (40.0) | 3 (60.0) | 0 (0.0) | 0 (0.0) |  |  | -0.029-0.829 |
| Health center/private hospital | 174 (74.7) | 30 (12.9) | 15 (6.4) | 14 (6.0) |  |  | 0.691-0.803 |
| **IV. Verbally sexual harassment** | | | | | | | |
| **Socio-demographic characteristics** |  |  |  |  |  |  |  |
| **Gender** |  |  |  |  | 38.950 | < 0.001 |  |
| Female | 925 (84.2) | 78 (7.1) | 62 (5.6) | 34 (3.1) |  |  | 0.820-0.863 |
| Male | 2,859 (90.2) | 178 (5.6) | 82 (2.6) | 49 (1.5) |  |  | 0.892-0.913 |
| **Age, yr** |  |  |  |  | 9.878 | 0.130 |  |
| < 30 | 1,127 (90.2) | 68 (5.4) | 36 (2.9) | 18 (1.4) |  |  | 0.886-0.919 |
| 30-40 | 1,676 (88.3) | 106 (5.6) | 70 (3.7) | 46 (2.4) |  |  | 0.869-0.897 |
| 40-50 | 768 (87.5) | 65 (7.4) | 29 (3.3) | 16 (1.8) |  |  | 0.853-0.897 |
| > 50 | 213 (88.0) | 17 (7.0) | 9 (3.7) | 3 (1.2) |  |  | 0.839-0.921 |
| **Marital status** |  |  |  |  | 1.558 | 0.669 |  |
| Single/living with partner | 703 (89.8) | 39 (5.0) | 25 (3.2) | 16 (2.0) |  |  | 0.877-0.919 |
| Married | 2,967 (88.5) | 204 (6.1) | 115 (3.4) | 66 (2.0) |  |  | 0.874-0.896 |
| Separated/divorced | 105 (86.8) | 11 (9.1) | 4 (3.3) | 1 (0.8) |  |  | 0.807-0.928 |
| Widowed | 9 (81.8) | 2 (18.2) | 0 (0.0) | 0 (0.0) |  |  | 0.590-1.046 |
| **Education** |  |  |  |  | 11.160 | 0.084 |  |
| 2-year associate’s degree | 113 (88.3) | 5 (3.9) | 9 (7.0) | 1 (0.8) |  |  | 0.827-0.939 |
| 3-year bachelor’s degree | 1,067 (90.3) | 65 (5.5) | 30 (2.5) | 19 (1.6) |  |  | 0.887-0.920 |
| Undergraduate diploma | 2,244 (88.4) | 152 (6.0) | 88 (3.5) | 54 (2.1) |  |  | 0.872-0.897 |
| Master’s degree | 302 (84.6) | 31 (8.7) | 16 (4.5) | 8 (2.2) |  |  | 0.808-0.883 |
| Doctoral degree | 58 (92.1) | 3 (4.8) | 1 (1.6) | 1 (1.6) |  |  | 0.854-0.987 |
| **Average monthly income, CNY** |  |  |  |  | 6.020 | 0.738 |  |
| < 2000 | 115 (89.1) | 8 (6.2) | 4 (3.1) | 2 (1.6) |  |  | 0.838-0.945 |
| 2000-4000 | 625 (89.0) | 42 (6.0) | 23 (3.3) | 12 (1.7) |  |  | 0.867-0.913 |
| 4000-6000 | 1,121 (89.8) | 67 (5.4) | 38 (3.0) | 22 (1.8) |  |  | 0.881-0.915 |
| 6000-8000 | 937 (88.3) | 64 (6.0) | 42 (4.0) | 18 (1.7) |  |  | 0.864-0.902 |
| > 8000 | 986 (87.5) | 75 (6.7) | 37 (3.3) | 29 (2.6) |  |  | 0.856-0.894 |
| **Health condition (rate 0-10)** |  |  |  |  | 40.665 | < 0.001 |  |
| Poor (0-2) | 41 (74.5) | 7 (12.7) | 4 (7.3) | 3 (5.5) |  |  | 0.630-0.861 |
| Bad (3-4) | 379 (81.9) | 42 (9.1) | 24 (5.2) | 18 (3.9) |  |  | 0.783-0.854 |
| Normal (5-6) | 1,374 (87.3) | 106 (6.7) | 62 (3.9) | 31 (2.0) |  |  | 0.857-0.890 |
| Healthy (7-8) | 1,739 (91.2) | 93 (4.9) | 49 (2.6) | 26 (1.4) |  |  | 0.899-0.925 |
| Good (9-10) | 251 (93.3) | 8 (3.0) | 5 (1.9) | 5 (1.9) |  |  | 0.903-0.963 |
| **Professional & Job-related Characteristics** |  |  |  |  |  |  |  |
| **Type of healthcare professional** |  |  |  |  | 39.124 | < 0.001 |  |
| Physician | 1,190 (86.1) | 103 (7.5) | 55 (4.0) | 34 (2.5) |  |  | 0.843-0.879 |
| Nurse/midwife | 1,669 (87.9) | 124 (6.5) | 69 (3.6) | 37 (1.9) |  |  | 0.864-0.894 |
| Pharmacist | 109 (93.2) | 3 (2.6) | 3 (2.6) | 2 (1.7) |  |  | 0.886-0.977 |
| Allied health professional (therapist/radiographer/assistant) | 362 (94.0) | 13 (3.4) | 5 (1.3) | 5 (1.3) |  |  | 0.917-0.964 |
| Administrative or clerical worker | 314 (92.9) | 11 (3.3) | 8 (2.4) | 5 (1.5) |  |  | 0.902-0.956 |
| Other | 140 (95.9) | 2 (1.4) | 4 (2.7) | 0 (0.0) |  |  | 0.927-0.991 |
| **Department** |  |  |  |  | 26.553 | 0.002 |  |
| General medicine | 1,179 (87.2) | 95 (7.0) | 49 (3.6) | 29 (2.1) |  |  | 0.854-0.890 |
| General surgery | 990 (86.3) | 79 (6.9) | 46 (4.0) | 32 (2.8) |  |  | 0.843-0.883 |
| Medical auxiliary/ancillary | 610 (92.0) | 33 (5.0) | 14 (2.1) | 6 (0.9) |  |  | 0.899-0.941 |
| Other | 1,005 (91.0) | 49 (4.4) | 35 (3.2) | 16 (1.4) |  |  | 0.893-0.926 |
| **Seniority** |  |  |  |  | 29.968 | < 0.001 |  |
| Senior | 94 (87.0) | 8 (7.4) | 4 (3.7) | 2 (1.9) |  |  | 0.807-0.934 |
| Deputy senior | 537 (83.0) | 61 (9.4) | 32 (4.9) | 17 (2.6) |  |  | 0.801-0.859 |
| Intermediate | 1,236 (88.9) | 82 (5.9) | 42 (3.0) | 31 (2.2) |  |  | 0.872-0.905 |
| Junior | 1,570 (89.8) | 92 (5.3) | 58 (3.3) | 29 (1.7) |  |  | 0.883-0.912 |
| Not reported | 347 (93.3) | 13 (3.5) | 8 (2.2) | 4 (1.1) |  |  | 0.907-0.958 |
| **Current position** |  |  |  |  | 5.477 | 0.140 |  |
| Hospital manager | 69 (83.1) | 6 (7.2) | 5 (6.0) | 3 (3.6) |  |  | 0.751-0.912 |
| Department manager | 760 (86.6) | 63 (7.2) | 38 (4.3) | 17 (1.9) |  |  | 0.843-0.888 |
| Employee | 2,888 (89.2) | 186 (5.7) | 100 (3.1) | 62 (1.9) |  |  | 0.882-0.903 |
| Intern/student/trainee | 67 (95.7) | 1 (1.4) | 1 (1.4) | 1 (1.4) |  |  | 0.910-1.005 |
| **Working experience, yr** |  |  |  |  | 21.412 | 0.002 |  |
| < 1 | 174 (95.1) | 5 (2.7) | 3 (1.6) | 1 (0.5) |  |  | 0.919-0.982 |
| 1-5 | 689 (92.9) | 26 (3.5) | 20 (2.7) | 7 (0.9) |  |  | 0.910-0.947 |
| 6-10 | 1,018 (86.9) | 83 (7.1) | 38 (3.2) | 32 (2.7) |  |  | 0.850-0.889 |
| > 10 | 1,903 (87.7) | 142 (6.5) | 83 (3.8) | 43 (2.0) |  |  | 0.863-0.890 |
| **Type of employee** |  |  |  |  | 3.861 | 0.277 |  |
| Employee under labor contract | 1,747 (87.7) | 128 (6.4) | 70 (3.5) | 46 (2.3) |  |  | 0.863-0.892 |
| Employee under contract | 1,950 (89.3) | 126 (5.8) | 72 (3.3) | 35 (1.6) |  |  | 0.880-0.906 |
| Temporary worker | 87 (93.5) | 2 (2.2) | 2 (2.2) | 2 (2.2) |  |  | 0.886-0.985 |
| **Night shift work** |  |  |  |  | 22.147 | < 0.001 |  |
| Yes | 2,356 (87.0) | 179 (6.6) | 107 (4.0) | 65 (2.4) |  |  | 0.858-0.883 |
| No | 1,428 (91.5) | 77 (4.9) | 37 (2.4) | 18 (1.2) |  |  | 0.902-0.929 |
| **Teaching duty** |  |  |  |  | 23.013 | < 0.001 |  |
| Yes | 1,839 (86.4) | 148 (7.0) | 92 (4.3) | 49 (2.3) |  |  | 0.850-0.879 |
| No | 1,945 (90.9) | 108 (5.0) | 52 (2.4) | 34 (1.6) |  |  | 0.897-0.921 |
| **Alignment between workload and income** |  |  |  |  | 73.025 | < 0.001 |  |
| Very poor | 281 (80.1) | 31 (8.8) | 20 (5.7) | 19 (5.4) |  |  | 0.759-0.842 |
| Poor | 1,044 (85.3) | 90 (7.4) | 58 (4.7) | 32 (2.6) |  |  | 0.833-0.873 |
| Normal | 1,465 (90.6) | 85 (5.3) | 46 (2.8) | 21 (1.3) |  |  | 0.892-0.920 |
| Aligned | 921 (92.6) | 46 (4.6) | 18 (1.8) | 10 (1.0) |  |  | 0.909-0.942 |
| Well | 73 (91.2) | 4 (5.0) | 2 (2.5) | 1 (1.2) |  |  | 0.851-0.974 |
| **Institutional Characteristics** |  |  |  |  |  |  |  |
| **Type of hospital** |  |  |  |  | 5.101 | 0.165 |  |
| General | 3,177 (88.3) | 221 (6.1) | 130 (3.6) | 69 (1.9) |  |  | 0.873-0.894 |
| Specialized | 607 (90.6) | 35 (5.2) | 14 (2.1) | 14 (2.1) |  |  | 0.884-0.928 |
| **Level of hospital** |  |  |  |  | 10.166 | 0.337 |  |
| Tertiary A | 1,697 (87.3) | 128 (6.6) | 75 (3.9) | 43 (2.2) |  |  | 0.859-0.888 |
| Tertiary B | 904 (90.2) | 58 (5.8) | 26 (2.6) | 14 (1.4) |  |  | 0.884-0.921 |
| Tertiary C | 6 (85.7) | 1 (14.3) | 0 (0.0) | 0 (0.0) |  |  | 0.598-1.116 |
| Secondary A | 868 (89.7) | 48 (5.0) | 34 (3.5) | 18 (1.9) |  |  | 0.878-0.916 |
| Secondary B | 96 (88.1) | 9 (8.3) | 2 (1.8) | 2 (1.8) |  |  | 0.820-0.942 |
| Secondary C | 5 (100.0) | 0 (0.0) | 0 (0.0) | 0 (0.0) |  |  | 1.000-1.000 |
| Health center/private hospital | 208 (89.3) | 12 (5.2) | 7 (3.0) | 6 (2.6) |  |  | 0.853-0.932 |
| **V. Physically sexual harassment** | | | | | | | |
| **Socio-demographic characteristics** |  |  |  |  |  |  |  |
| **Gender** |  |  |  |  | 38.950 | < 0.001 |  |
| Female | 925 (84.2) | 78 (7.1) | 62 (5.6) | 34 (3.1) |  |  | 0.820-0.863 |
| Male | 2,859 (90.2) | 178 (5.6) | 82 (2.6) | 49 (1.5) |  |  | 0.892-0.913 |
| **Age, yr** |  |  |  |  | 9.878 | 0.130 |  |
| < 30 | 1,127 (90.2) | 68 (5.4) | 36 (2.9) | 18 (1.4) |  |  | 0.886-0.919 |
| 30-40 | 1,676 (88.3) | 106 (5.6) | 70 (3.7) | 46 (2.4) |  |  | 0.869-0.897 |
| 40-50 | 768 (87.5) | 65 (7.4) | 29 (3.3) | 16 (1.8) |  |  | 0.853-0.897 |
| > 50 | 213 (88.0) | 17 (7.0) | 9 (3.7) | 3 (1.2) |  |  | 0.839-0.921 |
| **Marital status** |  |  |  |  | 1.558 | 0.669 |  |
| Single/living with partner | 703 (89.8) | 39 (5.0) | 25 (3.2) | 16 (2.0) |  |  | 0.877-0.919 |
| Married | 2,967 (88.5) | 204 (6.1) | 115 (3.4) | 66 (2.0) |  |  | 0.874-0.896 |
| Separated/divorced | 105 (86.8) | 11 (9.1) | 4 (3.3) | 1 (0.8) |  |  | 0.807-0.928 |
| Widowed | 9 (81.8) | 2 (18.2) | 0 (0.0) | 0 (0.0) |  |  | 0.590-1.046 |
| **Education** |  |  |  |  | 11.160 | 0.084 |  |
| 2-year associate’s degree | 113 (88.3) | 5 (3.9) | 9 (7.0) | 1 (0.8) |  |  | 0.827-0.939 |
| 3-year bachelor’s degree | 1,067 (90.3) | 65 (5.5) | 30 (2.5) | 19 (1.6) |  |  | 0.887-0.920 |
| Undergraduate diploma | 2,244 (88.4) | 152 (6.0) | 88 (3.5) | 54 (2.1) |  |  | 0.872-0.897 |
| Master’s degree | 302 (84.6) | 31 (8.7) | 16 (4.5) | 8 (2.2) |  |  | 0.808-0.883 |
| Doctoral degree | 58 (92.1) | 3 (4.8) | 1 (1.6) | 1 (1.6) |  |  | 0.854-0.987 |
| **Average monthly income, CNY** |  |  |  |  | 6.020 | 0.738 |  |
| < 2000 | 115 (89.1) | 8 (6.2) | 4 (3.1) | 2 (1.6) |  |  | 0.838-0.945 |
| 2000-4000 | 625 (89.0) | 42 (6.0) | 23 (3.3) | 12 (1.7) |  |  | 0.867-0.913 |
| 4000-6000 | 1,121 (89.8) | 67 (5.4) | 38 (3.0) | 22 (1.8) |  |  | 0.881-0.915 |
| 6000-8000 | 937 (88.3) | 64 (6.0) | 42 (4.0) | 18 (1.7) |  |  | 0.864-0.902 |
| > 8000 | 986 (87.5) | 75 (6.7) | 37 (3.3) | 29 (2.6) |  |  | 0.856-0.894 |
| **Health condition (rate 0-10)** |  |  |  |  | 40.665 | < 0.001 |  |
| Poor (0-2) | 41 (74.5) | 7 (12.7) | 4 (7.3) | 3 (5.5) |  |  | 0.630-0.861 |
| Bad (3-4) | 379 (81.9) | 42 (9.1) | 24 (5.2) | 18 (3.9) |  |  | 0.783-0.854 |
| Normal (5-6) | 1,374 (87.3) | 106 (6.7) | 62 (3.9) | 31 (2.0) |  |  | 0.857-0.890 |
| Healthy (7-8) | 1,739 (91.2) | 93 (4.9) | 49 (2.6) | 26 (1.4) |  |  | 0.899-0.925 |
| Good (9-10) | 251 (93.3) | 8 (3.0) | 5 (1.9) | 5 (1.9) |  |  | 0.903-0.963 |
|  |  |  |  |  |  |  |  |
| **Type of healthcare professional** |  |  |  |  | 39.124 | < 0.001 |  |
| Physician | 1,190 (86.1) | 103 (7.5) | 55 (4.0) | 34 (2.5) |  |  | 0.843-0.879 |
| Nurse/midwife | 1,669 (87.9) | 124 (6.5) | 69 (3.6) | 37 (1.9) |  |  | 0.864-0.894 |
| Pharmacist | 109 (93.2) | 3 (2.6) | 3 (2.6) | 2 (1.7) |  |  | 0.886-0.977 |
| Allied health professional (therapist/radiographer/assistant) | 362 (94.0) | 13 (3.4) | 5 (1.3) | 5 (1.3) |  |  | 0.917-0.964 |
| Administrative or clerical worker | 314 (92.9) | 11 (3.3) | 8 (2.4) | 5 (1.5) |  |  | 0.902-0.956 |
| Other | 140 (95.9) | 2 (1.4) | 4 (2.7) | 0 (0.0) |  |  | 0.927-0.991 |
| **Department** |  |  |  |  | 26.553 | 0.002 |  |
| General medicine | 1,179 (87.2) | 95 (7.0) | 49 (3.6) | 29 (2.1) |  |  | 0.854-0.890 |
| General surgery | 990 (86.3) | 79 (6.9) | 46 (4.0) | 32 (2.8) |  |  | 0.843-0.883 |
| Medical auxiliary/ancillary | 610 (92.0) | 33 (5.0) | 14 (2.1) | 6 (0.9) |  |  | 0.899-0.941 |
| Other | 1,005 (91.0) | 49 (4.4) | 35 (3.2) | 16 (1.4) |  |  | 0.893-0.926 |
| **Seniority** |  |  |  |  | 29.968 | < 0.001 |  |
| Senior | 94 (87.0) | 8 (7.4) | 4 (3.7) | 2 (1.9) |  |  | 0.807-0.934 |
| Deputy senior | 537 (83.0) | 61 (9.4) | 32 (4.9) | 17 (2.6) |  |  | 0.801-0.859 |
| Intermediate | 1,236 (88.9) | 82 (5.9) | 42 (3.0) | 31 (2.2) |  |  | 0.872-0.905 |
| Junior | 1,570 (89.8) | 92 (5.3) | 58 (3.3) | 29 (1.7) |  |  | 0.883-0.912 |
| Not reported | 347 (93.3) | 13 (3.5) | 8 (2.2) | 4 (1.1) |  |  | 0.907-0.958 |
| **Current position** |  |  |  |  | 5.477 | 0.140 |  |
| Hospital manager | 69 (83.1) | 6 (7.2) | 5 (6.0) | 3 (3.6) |  |  | 0.751-0.912 |
| Department manager | 760 (86.6) | 63 (7.2) | 38 (4.3) | 17 (1.9) |  |  | 0.843-0.888 |
| Employee | 2,888 (89.2) | 186 (5.7) | 100 (3.1) | 62 (1.9) |  |  | 0.882-0.903 |
| Intern/student/trainee | 67 (95.7) | 1 (1.4) | 1 (1.4) | 1 (1.4) |  |  | 0.910-1.005 |
| **Working experience, yr** |  |  |  |  | 21.412 | 0.002 |  |
| < 1 | 174 (95.1) | 5 (2.7) | 3 (1.6) | 1 (0.5) |  |  | 0.919-0.982 |
| 1-5 | 689 (92.9) | 26 (3.5) | 20 (2.7) | 7 (0.9) |  |  | 0.910-0.947 |
| 6-10 | 1,018 (86.9) | 83 (7.1) | 38 (3.2) | 32 (2.7) |  |  | 0.850-0.889 |
| > 10 | 1,903 (87.7) | 142 (6.5) | 83 (3.8) | 43 (2.0) |  |  | 0.863-0.890 |
| **Type of employee** |  |  |  |  | 3.861 | 0.277 |  |
| Employee under labor contract | 1,747 (87.7) | 128 (6.4) | 70 (3.5) | 46 (2.3) |  |  | 0.863-0.892 |
| Employee under contract | 1,950 (89.3) | 126 (5.8) | 72 (3.3) | 35 (1.6) |  |  | 0.880-0.906 |
| Temporary worker | 87 (93.5) | 2 (2.2) | 2 (2.2) | 2 (2.2) |  |  | 0.886-0.985 |
| **Night shift work** |  |  |  |  | 22.147 | < 0.001 |  |
| Yes | 2,356 (87.0) | 179 (6.6) | 107 (4.0) | 65 (2.4) |  |  | 0.858-0.883 |
| No | 1,428 (91.5) | 77 (4.9) | 37 (2.4) | 18 (1.2) |  |  | 0.902-0.929 |
| **Teaching duty** |  |  |  |  | 23.013 | < 0.001 |  |
| Yes | 1,839 (86.4) | 148 (7.0) | 92 (4.3) | 49 (2.3) |  |  | 0.850-0.879 |
| No | 1,945 (90.9) | 108 (5.0) | 52 (2.4) | 34 (1.6) |  |  | 0.897-0.921 |
| **Alignment between workload and income** |  |  |  |  | 73.025 | < 0.001 |  |
| Very poor | 281 (80.1) | 31 (8.8) | 20 (5.7) | 19 (5.4) |  |  | 0.759-0.842 |
| Poor | 1,044 (85.3) | 90 (7.4) | 58 (4.7) | 32 (2.6) |  |  | 0.833-0.873 |
| Normal | 1,465 (90.6) | 85 (5.3) | 46 (2.8) | 21 (1.3) |  |  | 0.892-0.920 |
| Aligned | 921 (92.6) | 46 (4.6) | 18 (1.8) | 10 (1.0) |  |  | 0.909-0.942 |
| Well | 73 (91.2) | 4 (5.0) | 2 (2.5) | 1 (1.2) |  |  | 0.851-0.974 |
| **Institutional Characteristics** |  |  |  |  |  |  |  |
| **Type of hospital** |  |  |  |  | 5.101 | 0.165 |  |
| General | 3,177 (88.3) | 221 (6.1) | 130 (3.6) | 69 (1.9) |  |  | 0.873-0.894 |
| Specialized | 607 (90.6) | 35 (5.2) | 14 (2.1) | 14 (2.1) |  |  | 0.884-0.928 |
| **Level of hospital** |  |  |  |  | 10.166 | 0.337 |  |
| Tertiary A | 1,697 (87.3) | 128 (6.6) | 75 (3.9) | 43 (2.2) |  |  | 0.859-0.888 |
| Tertiary B | 904 (90.2) | 58 (5.8) | 26 (2.6) | 14 (1.4) |  |  | 0.884-0.921 |
| Tertiary C | 6 (85.7) | 1 (14.3) | 0 (0.0) | 0 (0.0) |  |  | 0.598-1.116 |
| Secondary A | 868 (89.7) | 48 (5.0) | 34 (3.5) | 18 (1.9) |  |  | 0.878-0.916 |
| Secondary B | 96 (88.1) | 9 (8.3) | 2 (1.8) | 2 (1.8) |  |  | 0.820-0.942 |
| Secondary C | 5 (100.0) | 0 (0.0) | 0 (0.0) | 0 (0.0) |  |  | 1.000-1.000 |
| Health center/private hospital | 208 (89.3) | 12 (5.2) | 7 (3.0) | 6 (2.6) |  |  | 0.853-0.932 |

Note:

Physical assault (such as spitting, biting, hitting, pushing, etc.); emotional abuse (such as insults, humiliation, quarrelling, etc.); threats and intimidation (such as verbal, written, physical, or weapon threats); verbal sexual harassment (such as repeatedly discussing sexual privacy that one does not wish to mention); physical sexual harassment (such as unwanted touching or other forms).

# **Supplementary Table 2. Factors Associated with Workplace Violence Frequency: Ordered Logistic Regression Results.**

1. **WPV (0=never, 1=once, 2=2 - 3 times; 3=>4 times)**

|  | **WPV** | | | | |
| --- | --- | --- | --- | --- | --- |
|  | **β**  **(coefficient)** | **SE**  **(standard coefficient)** | **OR**  **(odds ratio)** | **95% CI** | **p value** |
| **Demographic Factors** | | | | | |
| **Gender** (ref: Male) |  |  |  |  |  |
| Female | -0.377 | 0.08 | 0.686*** | 0.587 - 0.802 | < 0.0001 |
| **Age** (per level increase) | -0.158 | 0.059 | 0.854*** | 0.760 - 0.960 | 0.008 |
| **Marriage** (ref: Unmarried) |  |  |  |  |  |
| Married | -0.067 | 0.096 | 0.935 | 0.776 - 1.128 | 0.4861 |
| **Education level** (per level increase) | -0.049 | 0.055 | 0.952 | 0.854 - 1.061 | 0.3766 |
| **Professional Characteristics** | | | | | |
| **Professional role** (ref: Doctor) |  |  |  |  |  |
| Other healthcare workers | 0.439 | 0.093 | 1.551*** | 1.292 - 1.862 | < 0.0001 |
| **Department** (ref: General medicine) |  |  |  |  |  |
| General surgery | -0.125 | 0.076 | 0.882 | 0.76 - 1.023 | 0.0977 |
| **Seniority** (per level decrease) | -0.161 | 0.054 | 0.851*** | 0.766 - 0.946 | 0.0027 |
| **Position** (per level decrease) | -0.302 | 0.077 | 0.739*** | 0.636 - 0.860 | 0.0001 |
| **Working experience** (per level increase) | 0.047 | 0.056 | 1.048 | 0.940 - 1.169 | 0.3959 |
| **Night shift** (ref: Yes) |  |  |  |  |  |
| No | -0.305 | 0.07 | 0.737*** | 0.643 - 0.845 | < 0.0001 |
| **Employment type** (ref: Formal) |  |  |  |  |  |
| Contract/Temporary | 0.039 | 0.075 | 1.04 | 0.897 - 1.206 | 0.6023 |
| **Teaching duty** (ref: Yes) |  |  |  |  |  |
| No | -0.233 | 0.071 | 0.792*** | 0.689 - 0.910 | <0.001 |
| **Type of hospital** (ref: General) |  |  |  |  |  |
| Specialty | -0.141 | 0.088 | 0.868 | 0.732 - 1.031 | 0.108 |
| **Level of hospital** (per level decrease) | -0.05 | 0.022 | 0.951** | 0.911 - 0.992 | 0.0193 |
| **Economic & Well-being Factors** | | | | | |
| **Average monthly income** (per level increase) | 0.195 | 0.039 | 1.215*** | 1.126 - 1.311 | < 0.0001 |
| Alignment between workload and income (per level decrease) | -0.19 | 0.039 | 0.827*** | 0.766 - 0.893 | < 0.0001 |
| Health condition (per level decrease) | -0.256 | 0.041 | 0.774*** | 0.715 - 0.838 | < 0.0001 |
| **Professional Values** |  |  |  |  |  |
| Intrinsic value (per point increase) | 0.041 | 0.012 | 1.042*** | 1.018 - 1.066 | 0.0006 |
| External value (per point increase) | -0.026 | 0.013 | 0.974 | 0.950 - 1.000 | 0.0539 |
| Social value (per point increase) | -0.042 | 0.01 | 0.959*** | 0.940 - 0.978 | < 0.0001 |
| Altruism value (per point increase) | 0.05 | 0.021 | 1.051** | 1.008 - 1.096 | 0.0195 |
| Leisure value (per point increase) | -0.05 | 0.007 | 0.951*** | 0.938 - 0.965 | < 0.0001 |
| **Threshold Parameters** | | | | | |
| Cut 1: 0 times │ 1 time | -0.31 | 0.031 |  |  | 0 |
| Cut 2: 1 time │ 2-3 times | -0.69 | 0.041 |  |  | 0 |
| Cut 3: 2-3 times │ ≥4 times | -0.046 | 0.031 |  |  | 0.1324 |

Exponentiated coefficients, Standard errors in parentheses

^*^ *p* < 0.1, ^**^ *p* < 0.05, ^***^ *p* < 0.01

1. **Physical assault (0=never, 1=once, 2=2 - 3 times; 3=>4 times)**

|  | **WPV** | | | | |
| --- | --- | --- | --- | --- | --- |
|  | **β**  **(coefficient)** | **SE**  **(standard coefficient)** | **OR**  **(odds ratio)** | **95% CI** | **p value** |
| **Demographic Factors** | | | | | |
| **Gender** (ref: Male) |  |  |  |  |  |
| Female | -0.731 | 0.107 | 0.481*** | 0.390 - 0.594 | < 0.0001 |
| **Age** (per level increase) | -0.143 | 0.081 | 0.867 | 0.739 - 1.017 | 0.0792 |
| **Marriage** (ref: Unmarried) |  |  |  |  |  |
| Married | -0.173 | 0.131 | 0.841 | 0.651 - 1.087 | 0.1861 |
| **Education level** (per level increase) | -0.112 | 0.075 | 0.894 | 0.772 - 1.035 | 0.1337 |
| **Professional Characteristics** | | | | | |
| **Professional role** (ref: Doctor) |  |  |  |  |  |
| Other healthcare workers | 0.557 | 0.129 | 1.745*** | 1.356 - 2.246 | < 0.0001 |
| **Department** (ref: General medicine) |  |  |  |  |  |
| General surgery | -0.166 | 0.099 | 0.847 | 0.697 - 1.028 | 0.0928 |
| **Seniority** (per level decrease) | -0.202 | 0.073 | 0.817*** | 0.708 - 0.943 | 0.0058 |
| **Position** (per level decrease) | -0.281 | 0.101 | 0.755*** | 0.620 - 0.920 | 0.0054 |
| **Working experience** (per level increase) | 0.124 | 0.077 | 1.132 | 0.973 - 1.318 | 0.1088 |
| **Night shift** (ref: Yes) |  |  |  |  |  |
| No | No | -0.337 | 0.714* | 0.589 - 0.865 | 0.098 |
| **Employment type** (ref: Formal) |  |  |  |  |  |
| Contract/Temporary | -0.012 | 0.103 | 0.988 | 0.807 - 1.21 | 0.9053 |
| **Teaching duty** (ref: Yes) |  |  |  |  |  |
| No | -0.285 | 0.098 | 0.752*** | 0.621 - 0.911 | 0.0036 |
| **Type of hospital** (ref: General) |  |  |  |  |  |
| Specialty | 0.192 | 0.119 | 1.212 | 0.959 - 1.532 | 0.108 |
| **Level of hospital** (per level decrease) | -0.066 | 0.03 | 0.936* | 0.882 - 0.994 | 0.0298 |
| **Economic & Well-being Factors** | | | | | |
| **Average monthly income** (per level increase) | 0.038 | 0.052 | 1.039 | 0.938 - 1.150 | 0.4698 |
| Alignment between workload and income (per level decrease) | -0.156 | 0.052 | 0.856*** | 0.773 - 0.947 | 0.0026 |
| Health condition (per level decrease) | -0.303 | 0.052 | 0.739*** | 0.667 - 0.818 | < 0.0001 |
| **Professional Values** |  |  |  |  |  |
| Intrinsic value (per point increase) | 0.002 | 0.015 | 1.002 | 0.972 - 1.033 | 0.8766 |
| External value (per point increase) | 0.002 | 0.018 | 1.002 | 0.968 - 1.037 | 0.9147 |
| Social value (per point increase) | -0.016 | 0.013 | 0.984 | 0.959 - 1.011 | 0.2461 |
| Altruism value (per point increase) | -0.01 | 0.027 | 0.990 | 0.938 - 1.045 | 0.7173 |
| Leisure value (per point increase) | -0.03 | 0.01 | 0.970*** | 0.953 - 0.989 | 0.0019 |
| **Threshold Parameters** | | | | | |
| Cut 1: 0 times │ 1 time | 1.388 | 0.039 |  |  | 0 |
| Cut 2: 1 time │ 2-3 times | -0.111 | 0.046 |  |  | 0.0144 |
| Cut 3: 2-3 times │ ≥4 times | 0.129 | 0.063 |  |  | 0.0407 |

1. **Emotional abuse (0=never, 1=once, 2=2 - 3 times; 3=>4 times)**

|  | **WPV** | | | | |
| --- | --- | --- | --- | --- | --- |
|  | **β**  **(coefficient)** | **SE**  **(standard coefficient)** | **OR**  **(odds ratio)** | **95% CI** | **p value** |
| **Demographic Factors** | | | | | |
| **Gender** (ref: Male) |  |  |  |  |  |
| Female | -0.247 | 0.08 | 0.781*** | 0.668 - 0.914 | 0.002 |
| **Age** (per level increase) | -0.185 | 0.06 | 0.831*** | 0.739 - 0.935 | 0.0021 |
| **Marriage** (ref: Unmarried) |  |  |  |  |  |
| Married | -0.099 | 0.097 | 0.906 | 0.748 - 1.096 | 0.3081 |
| **Education level** (per level increase) | -0.051 | 0.055 | 0.950 | 0.853 - 1.060 | 0.36 |
| **Professional Characteristics** | | | | | |
| **Professional role** (ref: Doctor) |  |  |  |  |  |
| Other healthcare workers | 0.474 | 0.094 | 1.606*** | 1.335 - 1.931 | < 0.0001 |
| **Department** (ref: General medicine) |  |  |  |  |  |
| General surgery | -0.133 | 0.076 | 0.875 | 0.755 - 1.015 | 0.0785 |
| **Seniority** (per level decrease) | -0.088 | 0.054 | 0.916 | 0.823 - 1.019 | 0.108 |
| **Position** (per level decrease) | -0.245 | 0.077 | 0.783*** | 0.673 - 0.911 | 0.0015 |
| **Working experience** (per level increase) | 0.065 | 0.056 | 1.067 | 0.955 - 1.191 | 0.2507 |
| **Night shift** (ref: Yes) |  |  |  |  |  |
| No | -0.263 | 0.071 | 0.769*** | 0.669 - 0.882 | 0.0002 |
| **Employment type** (ref: Formal) |  |  |  |  |  |
| Contract/Temporary | 0.037 | 0.076 | 1.038 | 0.894 - 1.205 | 0.6229 |
| **Teaching duty** (ref: Yes) |  |  |  |  |  |
| No | -0.213 | 0.072 | 0.808*** | 0.702 - 0.929 | 0.0028 |
| **Type of hospital** (ref: General) |  |  |  |  |  |
| Specialty | -0.18 | 0.089 | 0.835* | 0.702 - 0.994 | 0.042 |
| **Level of hospital** (per level decrease) | -0.032 | 0.022 | 0.969 | 0.928 - 1.011 | 0.1443 |
| **Economic & Well-being Factors** | | | | | |
| **Average monthly income** (per level increase) | 0.25 | 0.039 | 1.284*** | 1.189 - 1.386 | < 0.0001 |
| Alignment between workload and income (per level decrease) | -0.174 | 0.039 | 0.840*** | 0.779 - 0.907 | < 0.0001 |
| Health condition (per level decrease) | -0.23 | 0.04 | 0.795*** | 0.734 - 0.860 | < 0.0001 |
| **Professional Values** |  |  |  |  |  |
| Intrinsic value (per point increase) | 0.053 | 0.012 | 1.054*** | 1.030 - 1.080 | < 0.0001 |
| External value (per point increase) | -0.048 | 0.013 | 0.953*** | 0.928 - 0.978 | 0.0004 |
| Social value (per point increase) | -0.043 | 0.01 | 0.958*** | 0.938 - 0.977 | < 0.0001 |
| Altruism value (per point increase) | 0.077 | 0.021 | 1.080*** | 1.035 - 1.126 | 0.0004 |
| Leisure value (per point increase) | -0.052 | 0.007 | 0.949*** | 0.936 - 0.963 | < 0.0001 |
| **Threshold Parameters** | | | | | |
| Cut 1: 0 times │ 1 time | -0.151 | 0.031 |  |  | 0 |
| Cut 2: 1 time │ 2-3 times | -0.263 | 0.033 |  |  | 0 |
| Cut 3: 2-3 times │ ≥4 times | -0.048 | 0.034 |  |  | 0.1617 |

1. **Threats intimidation (0=never, 1=once, 2=2 - 3 times; 3=>4 times)**

|  | **WPV** | | | | |
| --- | --- | --- | --- | --- | --- |
|  | **β**  **(coefficient)** | **SE**  **(standard coefficient)** | **OR**  **(odds ratio)** | **95% CI** | **p value** |
| **Demographic Factors** | | | | | |
| **Gender** (ref: Male) |  |  |  |  |  |
| Female | -0.449 | 0.091 | 0.638*** | 0.534 - 0.764 | < 0.0001 |
| **Age** (per level increase) | -0.024 | 0.07 | 0.976 | 0.851 - 1.12 | 0.7285 |
| **Marriage** (ref: Unmarried) |  |  |  |  |  |
| Married | 0.084 | 0.118 | 1.088 | 0.863 - 1.37 | 0.478 |
| **Education level** (per level increase) | -0.018 | 0.065 | 0.982 | 0.865 - 1.115 | 0.785 |
| **Professional Characteristics** | | | | | |
| **Professional role** (ref: Doctor) |  |  |  |  |  |
| Other healthcare workers | 0.326 | 0.11 | 1.385 | 1.118 - 1.719 | 0.0029 |
| **Department** (ref: General medicine) |  |  |  |  |  |
| General surgery | -0.08 | 0.087 | 0.923 | 0.779 - 1.094 | 0.357 |
| **Seniority** (per level decrease) | -0.237 | 0.063 | 0.789*** | 0.697 - 0.893 | 0.0002 |
| **Position** (per level decrease) | -0.324 | 0.087 | 0.723*** | 0.61 - 0.858 | 0.0002 |
| **Working experience** (per level increase) | -0.066 | 0.067 | 0.936 | 0.821 - 1.068 | 0.3266 |
| **Night shift** (ref: Yes) |  |  |  |  |  |
| No | -0.368 | 0.084 | 0.692*** | 0.586 - 0.816 | < 0.0001 |
| **Employment type** (ref: Formal) |  |  |  |  |  |
| Contract/Temporary | 0.066 | 0.089 | 1.068 | 0.897 - 1.273 | 0.4573 |
| **Teaching duty** (ref: Yes) |  |  |  |  |  |
| No | -0.284 | 0.084 | 0.753*** | 0.638 - 0.888 | 0.0007 |
| **Type of hospital** (ref: General) |  |  |  |  |  |
| Specialty | -0.027 | 0.105 | 0.973 | 0.792 - 1.196 | 0.7963 |
| **Level of hospital** (per level decrease) | -0.081 | 0.027 | 0.922*** | 0.875 - 0.972 | 0.0024 |
| **Economic & Well-being Factors** | | | | | |
| **Average monthly income** (per level increase) | 0.13 | 0.045 | 1.139*** | 1.042 - 1.244 | 0.0043 |
| Alignment between workload and income (per level decrease) | -0.201 | 0.045 | 0.818*** | 0.749 - 0.893 | < 0.0001 |
| Health condition (per level decrease) | -0.284 | 0.046 | 0.753*** | 0.687 - 0.824 | < 0.0001 |
| **Professional Values** |  |  |  |  |  |
| Intrinsic value (per point increase) | 0.032 | 0.014 | 1.033*** | 1.005 - 1.060 | 0.0199 |
| External value (per point increase) | -0.006 | 0.015 | 0.994 | 0.965 - 1.025 | 0.7056 |
| Social value (per point increase) | -0.024 | 0.012 | 0.976** | 0.954 - 0.999 | 0.0394 |
| Altruism value (per point increase) | 0.024 | 0.024 | 1.024 | 0.977 - 1.074 | 0.3195 |
| Leisure value (per point increase) | -0.044 | 0.008 | 0.957*** | 0.941 - 0.973 | < 0.0001 |
| **Threshold Parameters** | | | | | |
| Cut 1: 0 times │ 1 time | 0.835 | 0.034 |  |  | 0 |
| Cut 2: 1 time │ 2-3 times | -0.048 | 0.036 |  |  | 0.1923 |
| Cut 3: 2-3 times │ ≥4 times | 0.031 | 0.052 |  |  | 0.5502 |

1. **Verbal sexual harassment (0=never, 1=once, 2=2 - 3 times; 3=>4 times)**

|  | **WPV** | | | | |
| --- | --- | --- | --- | --- | --- |
|  | **β**  **(coefficient)** | **SE**  **(standard coefficient)** | **OR**  **(odds ratio)** | **95% CI** | **p value** |
| **Demographic Factors** | | | | | |
| **Gender** (ref: Male) |  |  |  |  |  |
| Female | -0.81 | 0.135 | 0.445*** | 0.341 - 0.580 | < 0.0001 |
| **Age** (per level increase) | -0.105 | 0.102 | 0.900 | 0.737 - 1.100 | 0.3057 |
| **Marriage** (ref: Unmarried) |  |  |  |  |  |
| Married | -0.256 | 0.168 | 0.774 | 0.557 - 1.076 | 0.1273 |
| **Education level** (per level increase) | -0.055 | 0.095 | 0.946 | 0.786 - 1.139 | 0.559 |
| **Professional Characteristics** | | | | | |
| **Professional role** (ref: Doctor) |  |  |  |  |  |
| Other healthcare workers | 0.493 | 0.163 | 1.637 | 1.189 - 2.255 | 0.0025 |
| **Department** (ref: General medicine) |  |  |  |  |  |
| General surgery | 0.002 | 0.122 | 1.002 | 0.789 - 1.274 | 0.984 |
| **Seniority** (per level decrease) | -0.19 | 0.093 | 0.827** | 0.689 - 0.991 | 0.040 |
| **Position** (per level decrease) | -0.375 | 0.125 | 0.687*** | 0.537 - 0.878 | 0.0027 |
| **Working experience** (per level increase) | 0.236 | 0.099 | 1.266** | 1.043 - 1.538 | 0.0173 |
| **Night shift** (ref: Yes) |  |  |  |  |  |
| No | -0.354 | 0.125 | 0.702*** | 0.550 - 0.897 | 0.0046 |
| **Employment type** (ref: Formal) |  |  |  |  |  |
| Contract/Temporary | 0.126 | 0.13 | 1.134 | 0.879 - 1.465 | 0.3323 |
| **Teaching duty** (ref: Yes) |  |  |  |  |  |
| No | -0.222 | 0.123 | 0.801 | 0.630 - 1.018 | 0.0699 |
| **Type of hospital** (ref: General) |  |  |  |  |  |
| Specialty | -0.099 | 0.156 | 0.906 | 0.668 - 1.23 | 0.5268 |
| **Level of hospital** (per level decrease) | -0.011 | 0.037 | 0.989 | 0.920 - 1.065 | 0.778 |
| **Economic & Well-being Factors** | | | | | |
| **Average monthly income** (per level increase) | -0.068 | 0.065 | 0.934 | 0.823 - 1.061 | 0.2972 |
| Alignment between workload and income (per level decrease) | -0.277 | 0.065 | 0.758*** | 0.668 - 0.860 | < 0.0001 |
| Health condition (per level decrease) | -0.218 | 0.065 | 0.804*** | 0.708 - 0.913 | 0.0008 |
| **Professional Values** |  |  |  |  |  |
| Intrinsic value (per point increase) | -0.011 | 0.019 | 0.989 | 0.953 - 1.028 | 0.5838 |
| External value (per point increase) | 0.061 | 0.022 | 1.063*** | 1.018 - 1.111 | 0.0058 |
| Social value (per point increase) | -0.018 | 0.017 | 0.982 | 0.950 - 1.015 | 0.2847 |
| Altruism value (per point increase) | -0.048 | 0.035 | 0.953 | 0.891 - 1.020 | 0.165 |
| Leisure value (per point increase) | -0.024 | 0.012 | 0.976** | 0.954 - 0.999 | 0.0411 |
| **Threshold Parameters** | | | | | |
| Cut 1: 0 times │ 1 time | 2.082 | 0.049 |  |  | 0 |
| Cut 2: 1 time │ 2-3 times | -0.192 | 0.062 |  |  | 0.002 |
| Cut 3: 2-3 times │ ≥4 times | 0.044 | 0.086 |  |  | 0.6077 |

1. **Physical sexual harassment (0=never, 1=once, 2=2 - 3 times; 3=>4 times)**

|  | **WPV** | | | | |
| --- | --- | --- | --- | --- | --- |
|  | **β**  **(coefficient)** | **SE**  **(standard coefficient)** | **OR**  **(odds ratio)** | **95% CI** | **p value** |
| **Demographic Factors** | | | | | |
| **Gender** (ref: Male) |  |  |  |  |  |
| Female | -0.5 | 0.171 | 0.607*** | 0.434 - 0.849 | 0.0035 |
| **Age** (per level increase) | -0.204 | 0.13 | 0.815 | 0.631 - 1.052 | 0.117 |
| **Marriage** (ref: Unmarried) |  |  |  |  |  |
| Married | -0.143 | 0.2 | 0.867 | 0.586 - 1.284 | 0.4765 |
| **Education level** (per level increase) | -0.112 | 0.119 | 0.894 | 0.708 - 1.129 | 0.3481 |
| **Professional Characteristics** | | | | | |
| **Professional role** (ref: Doctor) |  |  |  |  |  |
| Other healthcare workers | 0.517 | 0.202 | 1.677** | 1.129 - 2.488 | 0.0104 |
| **Department** (ref: General medicine) |  |  |  |  |  |
| General surgery | 0.004 | 0.15 | 1.004 | 0.748 - 1.349 | 0.9774 |
| **Seniority** (per level decrease) | -0.273 | 0.118 | 0.761** | 0.603 - 0.959 | 0.0208 |
| **Position** (per level decrease) | -0.181 | 0.16 | 0.834 | 0.609 - 1.143 | 0.2595 |
| **Working experience** (per level increase) | 0.173 | 0.121 | 1.189 | 0.938 - 1.506 | 0.1531 |
| **Night shift** (ref: Yes) |  |  |  |  |  |
| No | -0.309 | 0.156 | 0.734** | 0.541 - 0.997 | 0.0481 |
| **Employment type** (ref: Formal) |  |  |  |  |  |
| Contract/Temporary | 0.337 | 0.163 | 1.401** | 1.019 - 1.927 | 0.0382 |
| **Teaching duty** (ref: Yes) |  |  |  |  |  |
| No | -0.16 | 0.151 | 0.852 | 0.633 - 1.146 | 0.2898 |
| **Type of hospital** (ref: General) |  |  |  |  |  |
| Specialty | 0.055 | 0.185 | 1.057 | 0.735 - 1.518 | 0.7671 |
| **Level of hospital** (per level decrease) | 0.022 | 0.046 | 1.022 | 0.934 - 1.119 | 0.6348 |
| **Economic & Well-being Factors** | | | | | |
| **Average monthly income** (per level increase) | 0.096 | 0.08 | 1.101 | 0.94 - 1.289 | 0.2332 |
| Alignment between workload and income (per level decrease) | -0.198 | 0.08 | 0.820** | 0.701 - 0.960 | 0.0134 |
| Health condition (per level decrease) | -0.286 | 0.081 | 0.751*** | 0.641 - 0.880 | 0.0004 |
| **Professional Values** |  |  |  |  |  |
| Intrinsic value (per point increase) | -0.031 | 0.024 | 0.969 | 0.926 - 1.016 | 0.2003 |
| External value (per point increase) | 0.063 | 0.028 | 1.065** | 1.008 - 1.125 | 0.0247 |
| Social value (per point increase) | -0.018 | 0.021 | 0.982 | 0.942 - 1.024 | 0.3921 |
| Altruism value (per point increase) | -0.066 | 0.043 | 0.936 | 0.860 - 1.019 | 0.1272 |
| Leisure value (per point increase) | -0.011 | 0.015 | 0.989 | 0.960 - 1.018 | 0.4484 |
| **Threshold Parameters** | | | | | |
| Cut 1: 0 times │ 1 time | 2.604 | 0.061 |  |  | 0 |
| Cut 2: 1 time │ 2-3 times | -0.013 | 0.076 |  |  | 0.8623 |
| Cut 3: 2-3 times │ ≥4 times | 0.048 | 0.121 |  |  | 0.6884 |
